# Review on solving the forward problem in EEG source analysis

**DOI:** 10.1186/1743-0003-4-46

**Published:** 2007-11-30

**Authors:** Hans Hallez, Bart Vanrumste, Roberta Grech, Joseph Muscat, Wim De Clercq, Anneleen Vergult, Yves D'Asseler, Kenneth P Camilleri, Simon G Fabri, Sabine Van Huffel, Ignace Lemahieu

**Affiliations:** 1 ELIS-MEDISIP, Ghent University, Ghent, Belgium; 2 ESAT, K.U.Leuven, Leuven, Belgium; 3 Katholieke Hogeschool Kempen, Geel, Belgium; 4 Department of Mathematics, University of Malta Junior College, Malta; 5 Faculty of Engineering, University of Malta, Malta; 6 Department of Mathematics, University of Malta, Malta

## Abstract

**Background:**

The aim of electroencephalogram (EEG) source localization is to find the brain areas responsible for EEG waves of interest. It consists of solving forward and inverse problems. The forward problem is solved by starting from a given electrical source and calculating the potentials at the electrodes. These evaluations are necessary to solve the inverse problem which is defined as finding brain sources which are responsible for the measured potentials at the EEG electrodes.

**Methods:**

While other reviews give an extensive summary of the both forward and inverse problem, this review article focuses on different aspects of solving the forward problem and it is intended for newcomers in this research field.

**Results:**

It starts with focusing on the generators of the EEG: the post-synaptic potentials in the apical dendrites of pyramidal neurons. These cells generate an extracellular current which can be modeled by Poisson's differential equation, and Neumann and Dirichlet boundary conditions. The compartments in which these currents flow can be anisotropic (e.g. skull and white matter). In a three-shell spherical head model an analytical expression exists to solve the forward problem. During the last two decades researchers have tried to solve Poisson's equation in a realistically shaped head model obtained from 3D medical images, which requires numerical methods. The following methods are compared with each other: the boundary element method (BEM), the finite element method (FEM) and the finite difference method (FDM). In the last two methods anisotropic conducting compartments can conveniently be introduced. Then the focus will be set on the use of reciprocity in EEG source localization. It is introduced to speed up the forward calculations which are here performed for each electrode position rather than for each dipole position. Solving Poisson's equation utilizing FEM and FDM corresponds to solving a large sparse linear system. Iterative methods are required to solve these sparse linear systems. The following iterative methods are discussed: successive over-relaxation, conjugate gradients method and algebraic multigrid method.

**Conclusion:**

Solving the forward problem has been well documented in the past decades. In the past simplified spherical head models are used, whereas nowadays a combination of imaging modalities are used to accurately describe the geometry of the head model. Efforts have been done on realistically describing the shape of the head model, as well as the heterogenity of the tissue types and realistically determining the conductivity. However, the determination and validation of the in vivo conductivity values is still an important topic in this field. In addition, more studies have to be done on the influence of all the parameters of the head model and of the numerical techniques on the solution of the forward problem.

## Introduction

Since the 1930s electrical activity of the brain has been measured by surface electrodes connected to the scalp [1]. Potential differences between these electrodes were then plotted as a function of time in a so-called electroencephalogram (EEG). The information extracted from these brain waves was, and still is instrumental in the diagnoses of neurological diseases [2], mainly epilepsy. Since the 1960s the EEG was also used to measure event-related potentials (ERPs). Here brain waves were triggered by a stimulus. These stimuli could be of visual, auditory and somatosensory nature. Different ERP protocols are now routinely used in a clinical neurophysiology lab. Researchers nowadays are still searching for new ERP protocols which may be able to distinguish between ERPs of patients with a certain condition and ERPs of normal subjects. This could be instrumental in disorders, such as psychiatric and developmental disorders, where there is often a lack of biological objective measures.

During the last two decades, increasing computational power has given researchers the tools to go a step further and try to find the underlying sources which generate the EEG. This activity is called EEG source localization. It consists of solving a forward and inverse problem. Solving the forward problem starts from a given electrical source configuration representing active neurons in the head. Then the potentials at the electrodes are calculated for this configuration. The inverse problem attempts to find the electrical source which generates a measured EEG. By solving the inverse problem, repeated solutions of the forward problem for different source configurations are needed. A review on solving the inverse problem is given in [3].

In this review article several aspects of solving the forward problem in EEG source localization will be discussed. It is intended for researchers new in the field to get insight in the state-of-the-art techniques to solve the forward problem in EEG source analysis. It also provides an extensive list of references to the work of other researchers.

First, the physical context of EEG source localization will be elaborated on and then the derivation of Poisson's equation with its boundary conditions. An analytical expression is then given for a three-shell spherical head model. Along with realistic head models, obtained from medical images, numerical methods are then introduced that are necessary to solve the forward problem. Several numerical techniques, the Boundary Element Method (BEM), the Finite Element Method (FEM) and the Finite Difference Method (FDM), will be discussed. Also anisotropic conductivities which can be found in the white matter compartment and skull, will be handled.

The reciprocity theorem used to speed up the calculations, is discussed. The electric field that results at the dipole location within the brain due to current injection and withdrawal at the surface electrode sites is first calculated. The forward transfer-coefficients are obtained from the scalar product of this electric field and the dipole moment. Calculations are thus performed for each electrode position rather than for each dipole position. This speeds up the time necessary to do the forward calculations since the number of electrodes is much smaller than the number of dipoles that need to be calculated.

The number of unknowns in the FEM and FDM can easily exceed the million and thus lead to large but sparse linear systems. As the number of unknowns is too large to solve the system in a direct manner, iterative solvers need to be used. Some popular iterative solvers are discussed such as successive over-relaxation (SOR), conjugate gradient method (CGM) and algebraic multigrid methods (AMG).

## The physics of EEG

In this section the physiology of the EEG will be shortly described. In our opinion, it is important to know the underlying mechanisms of the EEG. Moreover, forward modeling also involves a good model for the generators of the EEG. The mechanisms of the neuronal actionpotentials, excitatory post-synaptic potentials and inhibitory post-synaptic potentials are very complex. In this section we want to give a very comprehensive overview of the underlying neurophysiology.

### Neurophysiology

The brain consists of about 10^10 ^nerve cells or neurons. The shape and size of the neurons vary but they all possess the same anatomical subdivision. The soma or cell body contains the nucleus of the cell. The dendrites, arising from the soma and repeatedly branching, are specialized in receiving inputs from other nerve cells. Via the axon, impulses are sent to other neurons. The axon's end is divided into branches which form synapses with other neurons. The synapse is a specialized interface between two nerve cells. The synapse consists of a cleft between a presynaptic and postsynaptic neuron. At the end of the branches originating from the axon, the presynaptic neuron contains small rounded swellings which contain the neurotransmitter substance. Further readings on the anatomy of the brain can be found in [4] and [5].

One neuron generates a small amount of electrical activity. This small amount cannot be picked up by surface electrodes, as it is overwhelmed by other electrical activity from neighbouring neuron groups. When a large group of neurons is simultaneously active, the electrical activity is large enough to be picked up by the electrodes at the surface and thus generating the EEG. The electrical activity can be modeled as a current dipole. The current flow causes an electric field and also a potential field inside the human head. The electric field and potential field spreads to the surface of the head and an electrode at a certain point can measure the potential [2].

At rest the intracellular environment of a neuron is negatively polarized at approximately -70 mV compared with the extracellular environment. The potential difference is due to an unequal distribution of Na^+^, K^+ ^and Cl^- ^ions across the cell membrane. This unequal distribution is maintained by the Na^+ ^and K^+ ^ion pumps located in the cell membrane. The Goldman-Hodgkin-Katz equation describes this resting potential and this potential has been verified by experimental results [2,6,7].

The neuron's task is to process and transmit signals. This is done by an alternating chain of electrical and chemical signals. Active neurons secrete a neurotransmitter, which is a chemical substance, at the synaptical side. The synapses are mainly localized at the dendrites and the cell body of the postsynaptic cell. A postsynaptic neuron has a large number of receptors on its membrane that are sensitive for this neurotrans-mitter. The neurotransmitter in contact with the receptors changes the permeability of the membrane for charged ions. Two kinds of neurotransmitters exist. On the one hand there is a neurotransmitter which lets signals proliferate. These molecules cause an influx of positive ions. Hence depolarization of the intracellular space takes place. A depolarization means that the potential difference between the intra- and extracellular environment decreases. Instead of -70 mV the potential difference becomes -40 mV. This depolarization is also called an excitatory postsynaptic potential (EPSP). On the other hand there are neurotransmitters that stop the proliferation of signals. These molecules will cause an outflow of positive ions. Hence a hyperpolarization can be detected in the intracellular volume. A hyperpolarization means that the potential difference between the intra- and extracellular environment increases. This potential change is also called an inhibitory postsynaptic potential (IPSP). There are a large number of synapses from different presynaptic neurons in contact with one postsynaptic neuron. At the cell body all the EPSP and IPSP signals are integrated. When a net depolarization of the intracellular compartment at the cell body reaches a certain threshold, an action potential is generated. An action potential then propagates along the axon to other neurons [2,6,7].

**Figure 1:**
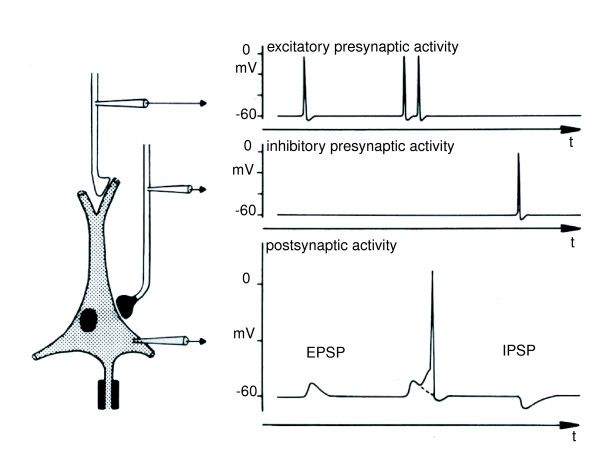
**Excitatory and inhibitory post synaptic potentials**. An illustration of the action potentials and post synaptic potentials measured at different locations at the neuron. On the left a neuron is displayed and three probes are drawn at the location where the potential is measured. The above picture on the right shows the incoming exitatory action potentials measured at the probe at the top, at the probe in the middle the incoming inhibitory action potential is measured and shown. The neuron processes the incoming potentials: the excitatory action potentials are transformed into excitatory post synaptic potentials, the inhibitory action potentials are transformed into inhibitory post synaptic potentials. When two excitatory post synaptic potentials occur in a small time frame, the neuron fires. This is shown at the bottom figure. The dotted line shows the EPSP, in case there was no second excitatory action potential following. From [2].

Figure 1 illustrates the excitatory and inhibitory postsynaptic potentials. It also shows the generation of an action potential. Further readings on the electrophysiology of neurons can be found in [2,6].

### The generators of the EEG

The electrodes used in scalp EEG are large and remote. They only detect summed activities of a large number of neurons which are synchronously electrically active. The action potentials can be large in amplitude (70–110 mV) but they have a small time course (0.3 ms). A synchronous firing of action potentials of neighboring neurons is unlikely. The postsynaptic potentials are the generators of the extracellular potential field which can be recorded with an EEG. Their time course is larger (10–20 ms). This enables summed activity of neighboring neurons. However their amplitude is smaller (0.1–10 mV) [3,8].

Apart from having more or less synchronous activity, the neurons need to be regularly arranged to have a measurable scalp EEG signal. The spatial properties of the neurons must be so that they amplify each other's extracellular potential fields. The neighboring pyramidal cells are organized so that the axes of their dendrite tree are parallel with each other and normal to the cortical surface. Hence, these cells are suggested to be the generators of the EEG.

**Figure 2:**
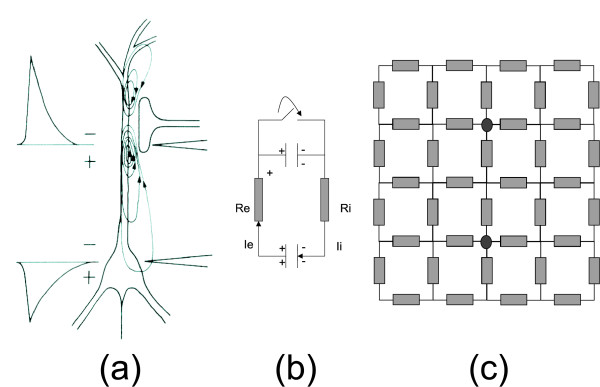
**equivalent circuit for a neuron**. An excitatory post synaptic potential, an simplified equivalent circuit for a neuron, and a resistive network for the extracellular environment. A neuron with an excitatory synapse at the apical dendrite is presented in (a). From [2]. A simplified equivalent circuit is depicted in (b). The extracellular environment can be represented with a resistive network as illustrated in (c).

The following is focused on excitatory synapses and EPSP, located at the apical dendrites of a pyramidal cell. The neurotransmitter in the excitatory synapses causes an influx of positive ions at the postsynaptic membrane as illustrated in figure 2(a) and depolarizes the local cell membrane. This causes a lack of extracellular positive ions at the apical dendrites of the postsynaptic neuron. A redistribution of positively charged ions also takes place at the intracellular side. These ions flow from the apical dendrite to the cell body and depolarize the membrane potentials at the cell body. Subsequently positive charged ions become available at the extracellular side at the cell body and basal dendrites.

A migration of positively charged ions from the cell body and the basal dendrites to the apical dendrite occurs, which is illustrated in figure 2(a) with current lines. This configuration generates extracellular potentials. Other membrane activities start to compensate for the massive intrusion of the positively charged ions at the apical dendrite, however these mechanisms are beyond the scope of this work and can be found elsewhere [2,9,10].

A simplified equivalent electric circuit is presented in figure 2(b) to illustrate the initial activity of an EPSP. At rest, the potential difference between the intra- and extracellular compartments can be represented by charged capacitors. One capacitor models the potential difference at the apical dendrites side while a second capacitor models the potential difference at the cell body and basal dendrite side. The potential difference over the capacitors is 60 mV. The neurotransmitter causes a massive intrusion of positively charged ions at the postsynaptic membrane at the apical dendrite side. In the equivalent circuit, this is modeled by a switch that is closed. The capacitor at the cell body side discharges causing a current flow over the extracellular resistor *R*_*e *_and the intracellular resistor *R*_*i*_. The repolarization of the cell membrane at the apical side or the initiation of the action potential are not modeled with this simple equivalent electrical circuit.

The capacitors and the switch, in figure 2(b), represent a model of the electrical source at the initial phase of the depolarization of the neuron. They could also be replaced by a time dependent current source, however this representation is not ideal. The capacitor representation, for the initial phase of depolarization, fits closer the occurring physical phenomena. The impedance of the tissue in the human head has, for the frequencies contained in the EEG, no capacitive nor inductive component and is hence pure resistive. More advanced equivalent electrical circuits can be found elsewhere [10]. The fact that a current flows through the extracellular resistor indicates that potential differences in the extracellular space can be measured.

A simplified electrical model for this active cell consists of two current monopoles: a current sink at the apical dendrite side which removes positively charged ions from the extracellular environment, and a current source at the cell body side which injects positively charged ions in the extracellular environment. The extracellular resistance *R*_*e *_can be decomposed in the volume conductor model in which the active neuron is embedded, as illustrated in figure 2(c). For further reading on the generation of the EEG one can refer to [11] and [9].

## Poisson's equation, boundary conditions and dipoles

In the previous sections we saw that the generators of the EEG are the synaptic potentials along the apical dendrites of the pyramidal cells of the grey matter cortex. It is important to notice that the EEG reflects the electrical activity of a subgroup of neurons, especially pyramidal neuron cells, where the apical dendrite is systematically oriented orthogonal to the brain surface. Certain types of neurons are not systematically oriented orthogonal to the brain surface. Therefore, the potential fields of the synaptic currents at different dendrites of neurons van cancel each other out. In that case the neuronal activity is not visible at the surface. Moreover, that actionpotentials, propagating along the axons, have no influence on the EEG. Their short timespan (2 *ms*) make the chance of generating simultaneous actionpotentials very small [6,12]. In this section, a mathematical approach on the generation of the forward problem is given.

### Quasi-static conditions

It is shown in [13] that no charge can be piled up in the conducting extracellular volume for the frequency range of the signals measured in the EEG. At one moment in time all the fields are triggered by the active electric source. Hence, no time delay effects are introduced. All fields and currents behave as if they were stationary at each instance. These conditions are also called quasi-static conditions. They are not static because the neural activity changes with time. But the changes are slow compared to the propagation effects.

### Applying the divergence operator to the current density

Poisson's equation gives a relationship between the potentials at any position in a volume conductor and the applied current sources. The mathematical derivation of Poisson's equation via Maxwell's equations, can be found in various textbooks on electromagnetism [6,10,14]. Poisson's equation is derived with the divergence operator. In this way the emphasis is, in our opinion, more on the physical aspect of the problem. Furthermore, the concepts introduced in [10,14], such as current source and current sink, are used when applying the divergence operator.

#### Definition

The current density is a vector field and can be represented by **J**(*x*, *y*, *z*). The unit of the current density is *A/m*^2^. The divergence of a vector field **J **is defined as follows:

∇⋅J=lim⁡G→01G∮∂GJdS
 MathType@MTEF@5@5@+=feaafiart1ev1aaatCvAUfKttLearuWrP9MDH5MBPbIqV92AaeXatLxBI9gBaebbnrfifHhDYfgasaacPC6xNi=xI8qiVKYPFjYdHaVhbbf9v8qqaqFr0xc9vqFj0dXdbba91qpepeI8k8fiI+fsY=rqGqVepae9pg0db9vqaiVgFr0xfr=xfr=xc9adbaqaaeGacaGaaiaabeqaaeqabiWaaaGcbaGaey4bIeTaeyyXICncbeGae8NsaOKaeyypa0ZaaCbeaeaacyGGSbaBcqGGPbqAcqGGTbqBaSqaaiabdEeahjabgkziUkabicdaWaqabaqcfa4aaSaaaeaacqaIXaqmaeaacqWGhbWraaGcdaWdvaqaaiab=PeakjabdsgaKjab=nfatbWcbaGaeyOaIyRaem4raCeabeqdcqWIr4E0cqGHRiI8aaaa@4793@

The integral over a closed surface ∂*G *represents a flux or a current. This integral is positive when a net current leaves the volume *G *and is negative when a net current enters the volume *G*. The vector *d***S **for a surface element of ∂*G *with area *dS *and outward normal **e**_*n*_, can also be written as **e**_*n*_*dS*. The unit of ∇·**J **is *A/m*^3 ^and is often called the current source density which in [15] is symbolized with *I*_*m*_. Generally one can write:

∇·**J **= *I*_*m*_.

#### Applying the divergence operator to the extracellular current density

First a small volume in the extracellular space, which encloses a current source and current sink, is investigated. The current flowing into the infinitely small volume, must be equal to the current leaving that volume. This is due to the fact that no charge can be piled up in the extracellular space. The surface integral of equation (1) is then zero, hence ∇·**J **= 0.

In the second case a volume enclosed by the current sink with position parameters **r**_1_(*x*_1_, *y*_1_, *z*_1_) is assumed. The current sink represents the removal of positively charged ions at the apical dendrite of the pyramidal cell. The integral of equation (1) remains equal to -*I *while the volume *G *in the denominator becomes infinitesimally small. This gives a singularity for the current source density. This singularity can be written as a delta function: -*Iδ*(**r **- **r**_1_). The negative sign indicates that current is removed from the extracellular volume. The delta function indicates that current is removed at one point in space.

**Figure 3:**
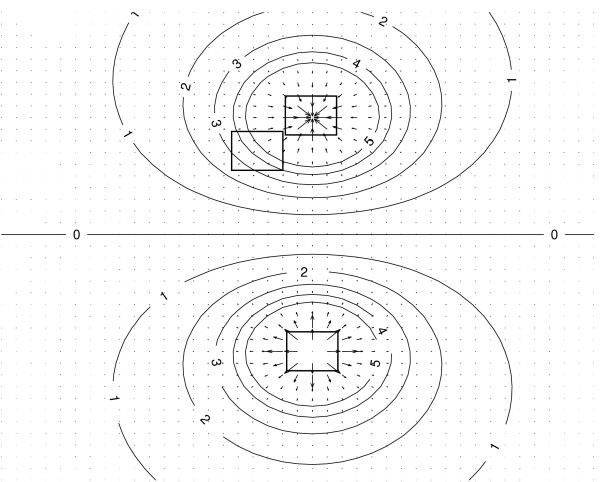
**The current density and equipotential lines in the vicinity of a dipole**. The current density and equipotential lines in the vicinity of a current source and current sink is depicted. Equipotential lines are also given. Boxes are illustrated which represent the volumes *G*.

For the third case a small volume around the current source at position **r**_2_(*x*_2_, *y*_2_, *z*_2_) is constructed. The current source represents the injection of positively charged ions at the cell body of the pyramidal cell. The current source density equals *Iδ*(**r **- **r**_2_). Figure 3 represents the current density vectors for a current source and current sink configuration. Furthermore, three boxes are presented corresponding with the three cases discussed above.

Uniting the three cases given above, one obtains:

∇·**J **= *Iδ*(**r **- **r**_2_) - *Iδ*(**r **- **r**_1_).

### Ohm's law, the potential field and anisotropic/isotropic conductivities

The relationship between the current density **J **in *A/m*^2 ^and the electric field **E **in *V/m *is given by Ohm's law:

**J **= *σ***E**,

with *σ*(**r**) ∈ ℝ^3×3 ^being the position dependent conductivity tensor given by:

σ=[σ11σ12σ13σ12σ22σ23σ13σ23σ33],
 MathType@MTEF@5@5@+=feaafiart1ev1aaatCvAUfKttLearuWrP9MDH5MBPbIqV92AaeXatLxBI9gBaebbnrfifHhDYfgasaacPC6xNi=xI8qiVKYPFjYdHaVhbbf9v8qqaqFr0xc9vqFj0dXdbba91qpepeI8k8fiI+fsY=rqGqVepae9pg0db9vqaiVgFr0xfr=xfr=xc9adbaqaaeGacaGaaiaabeqaaeqabiWaaaGcbaacciGae83WdmNaeyypa0ZaamWaaeaafaqabeWadaaabaGae83Wdm3aaSbaaSqaaiabigdaXiabigdaXaqabaaakeaacqWFdpWCdaWgaaWcbaGaeGymaeJaeGOmaidabeaaaOqaaiab=n8aZnaaBaaaleaacqaIXaqmcqaIZaWmaeqaaaGcbaGae83Wdm3aaSbaaSqaaiabigdaXiabikdaYaqabaaakeaacqWFdpWCdaWgaaWcbaGaeGOmaiJaeGOmaidabeaaaOqaaiab=n8aZnaaBaaaleaacqaIYaGmcqaIZaWmaeqaaaGcbaGae83Wdm3aaSbaaSqaaiabigdaXiabiodaZaqabaaakeaacqWFdpWCdaWgaaWcbaGaeGOmaiJaeG4mamdabeaaaOqaaiab=n8aZnaaBaaaleaacqaIZaWmcqaIZaWmaeqaaaaaaOGaay5waiaaw2faaiabcYcaSaaa@5472@

and with units *A/*(*Vm*) = *S/m*. There are tissues in the human head that have an anisotropic conductivity. This means that the conductivity is not equal in every direction and that the electric field can induce a current density component perpendicular to it with the appropriate *σ *in equation (4).

**Figure 4:**
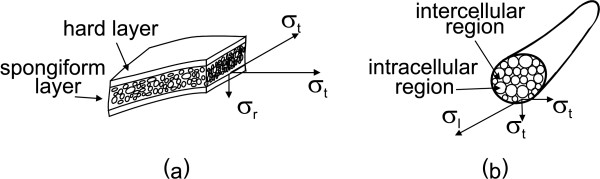
**Anisotropic conductivity of the brain tissues**. The anisotropic properties of the conductivity of skull and white matter tissues. The anisotropic properties of the conductivity of skull and white matter tissues. (a) The skull consists of 3 layers: a spongiform layer between two hard layers. The conductivity tangentially to the skull surface is 10 times larger than the radial conductivity. (b) White matter consist of axons, grouped in bundles. The conductivity along the nerve bundle is 9 times larger than perpendicular to the nerve bundle.

At the skull, for example, the conductivity tangential to the surface is 10 times [16] the conductivity perpendicular to the surface (see figure 4(a)). The rationale for this is that the skull consists of 3 layers: a spongiform layer between two hard layers. Water, and also ionized particles, can move easily through the spongiform layer, but not through the hard layers [17]. Wolters et al. state that skull anisotropy has a smearing effect on the forward potential computation. The deeper a source lies, the more it is surrounded by anisotropic tissue, the larger the influence of the anisotropy on the resulting electric field. Therefore, the presence of anisotropic conducting tissues compromises the forward potential computation and as a consequence, the inverse problem [18].

White matter consists of different nerve bundles (groups of axons) connecting cortical grey matter (mainly dendrites and cell bodies). The nerve bundles consist of nerve fibres or axons (see figure 4(b)). Water and ionized particles can move more easily along the nerve bundle than perpendicular to the nerve bundle. Therefore, the conductivity along the nerve bundle is measured to be 9 times higher than perpendicular to it [19,20]. The nerve bundle direction can be estimated by a recent magnetic resonance technique: diffusion tensor magnetic resonance imaging (DT-MRI) [21]. This technique provides directional information on the diffusion of water. It is assumed that the conductivity is the highest in the direction in which the water diffuses most easily [22]. Authors [23-25] have showed that anisotropic conducting compartments should be incorporated in volume conductor models of the head whenever possible.

In the grey matter, scalp and cerebro-spinal fluid (CSF) the conductivity is equal in all directions. Thus the place dependent conductivity tensor becomes a place dependent scalar *σ*, a so-called isotropic conducting tissue. The conductivity of CSF is quite accurately known to be 1.79 S/m [26]. In the following we will focus on the conductivity of the skull and soft tissues. Some typical values of conductivities can be found in table 1.

**Table 1:** The reference values of the absolute and relative conductivity of the compartments incorporated in the human head.

compartments	Geddes & Baker (1967)	Oostendorp (2000)	Gonçalves (2003)	Guttierrez (2004)	Lai (2005)
scalp	0.43	0.22	0.33	0.749	0.33
skull	0.006 – 0.015	0.015	0.0081	0.012	0.0132
cerebro-spinal fluid	-	-	-	1.79	-
brain	0.12 – 0.48	0.22	0.33	0.313	0.33
*σ*_*scalp*_/*σ*_*skull*_	80	15	20–50	26	25

The skull conductivity has been subject to debate among researchers. In vivo measurements are very different from in vitro measurements. On top of that, the measurements are very patient specific. In [27], it was stated that the skull conductivity has a large influence on the forward problem.

It was believed that the conductivity ratio between skull and soft tissue (scalp and brain) was on average 80 [20]. Oostendorp et al. used a technique with realistic head models by which they passed a small current by means of 2 electrodes placed on the scalp. A potential distribution is then generated on the scalp. Because the potential values and the current source and sink are known, only the conductivities are unknown in the head model and equation (4) can be solved toward *σ*. Using this technique they could estimate the skull-to-soft tissue conductivity ratio to be 15 instead of 80 [28]. At the same time, Ferree et al. did a similar study using spherical head models. Here, skull-to-soft tissue conductivity was calculated as 25. It was shown in [29] that using a ratio of 80 instead of 16, could yield EEG source localization errors of an average of 3 cm up to 5 cm.

One can repeat the previous experiment for a lot of different electrode pairs and an image of the conductivity can be obtained. This technique is called electromagnetic impedance tomography or EIT. In short, EIT is an inverse problem, by which the conductivities are estimated. Using this technique, the skull-to-soft tissue conductivity ratio was estimated to be around 20–25 [30,31]. However in [30], it was shown that the skull-to-soft tissue ratio could differ from patient to patient with a factor 2.4. In [32], maximum likelihood and maximum a posteriori techniques are used to simultaneously estimate the different conductivities. There they estimated the skull-to-soft tissue ratio to be 26.

Another study came to similar results using a different technique. In Lai et al., the authors used intracranial and scalp electrodes to get an estimation of the skull-to-soft tissue ratio conductivity. From the scalp measures they estimated the cortical activity by means of a cortical imaging technique. The conductivity ratio was adjusted so that the intracranial measurements were consistent with the result of the imaging from the scalp technique. They resulted in a ratio of 25 with a standard deviation of 7. One has to note however that the study was performed on pediatric patients which had the age of between 8 and 12. Their skull tissue normally contains a larger amount of ions and water and so may have a higher conductivity than the adults calcified cranial bones [33]. In a more experimental setting, the authors of [34] performed conductivity measures on the skull itself in patients undergoing epilepsy surgery. Here the authors estimated the skull conductivity to be between 0.032 and 0.080 S/m, which comes down to a soft-tissue to skull conductivity of 10 to 40.

### Poisson's equation

The scalar potential field *V*, having volt as unit, is now introduced. This is possible due to Faraday's law being zero under quasi-static conditions (∇ × **E **= **0**) [35]. The link between the potential field and the electric field is given utilizing the gradient operator,

**E **= -∇*V*.

The vector ∇*V *at a point gives the direction in which the scalar field *V*, having volt as its unit, most rapidly increases. The minus sign in equation (6) indicates that the electric field is oriented from an area with a high potential to an area with a low potential. Figure 3 also illustrates some equipotential lines generated by a current source and current a sink.

When equation (2), equation (4) and equation (6) are combined, Poisson's differential equation is obtained in general form:

∇·(*σ*∇(*V*)) = -*I*_*m*_.

For the problem at hand, equation (3), equation (4) and equation (6) are combined yielding:

∇·(*σ*∇(*V*)) = -*Iδ*(**r **- **r**_2_) + *Iδ*(**r **- **r**_1_).

In the Cartesian coordinate system equation (8) becomes for isotropic conductivities:

∂∂x(σ∂V∂x)+∂∂y(σ∂V∂y)+∂∂z(σ∂V∂z)=−Iδ(x−x2)δ(y−y2)δ(z−z2)+Iδ(x−x1)δ(y−y1)δ(z−z1)
 MathType@MTEF@5@5@+=feaafiart1ev1aaatCvAUfKttLearuWrP9MDH5MBPbIqV92AaeXatLxBI9gBaebbnrfifHhDYfgasaacPC6xNi=xI8qiVKYPFjYdHaVhbbf9v8qqaqFr0xc9vqFj0dXdbba91qpepeI8k8fiI+fsY=rqGqVepae9pg0db9vqaiVgFr0xfr=xfr=xc9adbaqaaeGacaGaaiaabeqaaeqabiWaaaGcbaqbaeaabiGaaaqaaKqbaoaalaaabaGaeyOaIylabaGaeyOaIyRaemiEaGhaaOGaeiikaGccciGae83Wdmxcfa4aaSaaaeaacqGHciITcqWGwbGvaeaacqGHciITcqWG4baEaaGccqGGPaqkcqGHRaWkjuaGdaWcaaqaaiabgkGi2cqaaiabgkGi2kabdMha5baakiabcIcaOiab=n8aZLqbaoaalaaabaGaeyOaIyRaemOvayfabaGaeyOaIyRaemyEaKhaaOGaeiykaKIaey4kaSscfa4aaSaaaeaacqGHciITaeaacqGHciITcqWG6bGEaaGccqGGOaakcqWFdpWCjuaGdaWcaaqaaiabgkGi2kabdAfawbqaaiabgkGi2kabdQha6baakiabcMcaPiabg2da9aqaaiabgkHiTiabdMeajjab=r7aKjabcIcaOiabdIha4jabgkHiTiabdIha4naaBaaaleaacqaIYaGmaeqaaOGaeiykaKIae8hTdqMaeiikaGIaemyEaKNaeyOeI0IaemyEaK3aaSbaaSqaaiabikdaYaqabaGccqGGPaqkcqWF0oazcqGGOaakcqWG6bGEcqGHsislcqWG6bGEdaWgaaWcbaGaeGOmaidabeaakiabcMcaPaqaaaqaaiabgUcaRiabdMeajjab=r7aKjabcIcaOiabdIha4jabgkHiTiabdIha4naaBaaaleaacqaIXaqmaeqaaOGaeiykaKIae8hTdqMaeiikaGIaemyEaKNaeyOeI0IaemyEaK3aaSbaaSqaaiabigdaXaqabaGccqGGPaqkcqWF0oazcqGGOaakcqWG6bGEcqGHsislcqWG6bGEdaWgaaWcbaGaeGymaedabeaakiabcMcaPaaaaaa@90BC@

and for anisotropic conductivities:

σ11∂2V∂x2+σ22∂2V∂y2+σ33∂2V∂z2+2(σ12∂2V∂x∂y+σ13∂2V∂x∂z+σ23∂2V∂y∂z)+(∂σ11∂x+∂σ12∂y+∂σ13∂z)∂V∂x+(∂σ12∂x+∂σ22∂y+∂σ23∂z)∂V∂y+(∂σ13∂x+∂σ23∂y+∂σ33∂z)∂V∂z=−Iδ(x−x2)δ(y−y2)δ(z−z2)+Iδ(x−x1)δ(y−y1)δ(z−z1).
 MathType@MTEF@5@5@+=feaafiart1ev1aaatCvAUfKttLearuWrP9MDH5MBPbIqV92AaeXatLxBI9gBaebbnrfifHhDYfgasaacPC6xNi=xI8qiVKYPFjYdHaVhbbf9v8qqaqFr0xc9vqFj0dXdbba91qpepeI8k8fiI+fsY=rqGqVepae9pg0db9vqaiVgFr0xfr=xfr=xc9adbaqaaeGacaGaaiaabeqaaeqabiWaaaGcbaqbaeqabmqaaaqaaGGaciab=n8aZnaaBaaaleaacqaIXaqmcqaIXaqmaeqaaKqbaoaalaaabaGaeyOaIy7aaWbaaeqabaGaeGOmaidaaiabdAfawbqaaiabgkGi2kabdIha4naaCaaabeqaaiabikdaYaaaaaGccqGHRaWkcqWFdpWCdaWgaaWcbaGaeGOmaiJaeGOmaidabeaajuaGdaWcaaqaaiabgkGi2oaaCaaabeqaaiabikdaYaaacqWGwbGvaeaacqGHciITcqWG5bqEdaahaaqabeaacqaIYaGmaaaaaOGaey4kaSIae83Wdm3aaSbaaSqaaiabiodaZiabiodaZaqabaqcfa4aaSaaaeaacqGHciITdaahaaqabeaacqaIYaGmaaGaemOvayfabaGaeyOaIyRaemOEaO3aaWbaaeqabaGaeGOmaidaaaaakiabgUcaRiabikdaYmaabmaabaGae83Wdm3aaSbaaSqaaiabigdaXiabikdaYaqabaqcfa4aaSaaaeaacqGHciITdaahaaqabeaacqaIYaGmaaGaemOvayfabaGaeyOaIyRaemiEaGNaeyOaIyRaemyEaKhaaOGaey4kaSIae83Wdm3aaSbaaSqaaiabigdaXiabiodaZaqabaqcfa4aaSaaaeaacqGHciITdaahaaqabeaacqaIYaGmaaGaemOvayfabaGaeyOaIyRaemiEaGNaeyOaIyRaemOEaOhaaOGaey4kaSIae83Wdm3aaSbaaSqaaiabikdaYiabiodaZaqabaqcfa4aaSaaaeaacqGHciITdaahaaqabeaacqaIYaGmaaGaemOvayfabaGaeyOaIyRaemyEaKNaeyOaIyRaemOEaOhaaaGccaGLOaGaayzkaaaabaGaey4kaSYaaeWaaeaajuaGdaWcaaqaaiabgkGi2kab=n8aZnaaBaaabaGaeGymaeJaeGymaedabeaaaeaacqGHciITcqWG4baEaaGccqGHRaWkjuaGdaWcaaqaaiabgkGi2kab=n8aZnaaBaaabaGaeGymaeJaeGOmaidabeaaaeaacqGHciITcqWG5bqEaaGccqGHRaWkjuaGdaWcaaqaaiabgkGi2kab=n8aZnaaBaaabaGaeGymaeJaeG4mamdabeaaaeaacqGHciITcqWG6bGEaaaakiaawIcacaGLPaaajuaGdaWcaaqaaiabgkGi2kabdAfawbqaaiabgkGi2kabdIha4baakiabgUcaRmaabmaabaqcfa4aaSaaaeaacqGHciITcqWFdpWCdaWgaaqaaiabigdaXiabikdaYaqabaaabaGaeyOaIyRaemiEaGhaaOGaey4kaSscfa4aaSaaaeaacqGHciITcqWFdpWCdaWgaaqaaiabikdaYiabikdaYaqabaaabaGaeyOaIyRaemyEaKhaaOGaey4kaSscfa4aaSaaaeaacqGHciITcqWFdpWCdaWgaaqaaiabikdaYiabiodaZaqabaaabaGaeyOaIyRaemOEaOhaaaGccaGLOaGaayzkaaqcfa4aaSaaaeaacqGHciITcqWGwbGvaeaacqGHciITcqWG5bqEaaGccqGHRaWkdaqadaqaaKqbaoaalaaabaGaeyOaIyRae83Wdm3aaSbaaeaacqaIXaqmcqaIZaWmaeqaaaqaaiabgkGi2kabdIha4baakiabgUcaRKqbaoaalaaabaGaeyOaIyRae83Wdm3aaSbaaeaacqaIYaGmcqaIZaWmaeqaaaqaaiabgkGi2kabdMha5baakiabgUcaRKqbaoaalaaabaGaeyOaIyRae83Wdm3aaSbaaeaacqaIZaWmcqaIZaWmaeqaaaqaaiabgkGi2kabdQha6baaaOGaayjkaiaawMcaaKqbaoaalaaabaGaeyOaIyRaemOvayfabaGaeyOaIyRaemOEaOhaaOGaeyypa0dabaGaeyOeI0IaemysaKKae8hTdqMaeiikaGIaemiEaGNaeyOeI0IaemiEaG3aaSbaaSqaaiabikdaYaqabaGccqGGPaqkcqWF0oazcqGGOaakcqWG5bqEcqGHsislcqWG5bqEdaWgaaWcbaGaeGOmaidabeaakiabcMcaPiab=r7aKjabcIcaOiabdQha6jabgkHiTiabdQha6naaBaaaleaacqaIYaGmaeqaaOGaeiykaKIaey4kaSIaemysaKKae8hTdqMaeiikaGIaemiEaGNaeyOeI0IaemiEaG3aaSbaaSqaaiabigdaXaqabaGccqGGPaqkcqWF0oazcqGGOaakcqWG5bqEcqGHsislcqWG5bqEdaWgaaWcbaGaeGymaedabeaakiabcMcaPiab=r7aKjabcIcaOiabdQha6jabgkHiTiabdQha6naaBaaaleaacqaIXaqmaeqaaOGaeiykaKIaeiOla4caaaaa@2676@

The potentials *V *are calculated with equations (8), (9) or (10) for a given current source density *I*_*m*_, in a volume conductor model, e.g. in our application, the human head. Compartments in which all conductivities are equal, are called homogeneous conducting compartments.

### Boundary conditions

**Figure 5:**
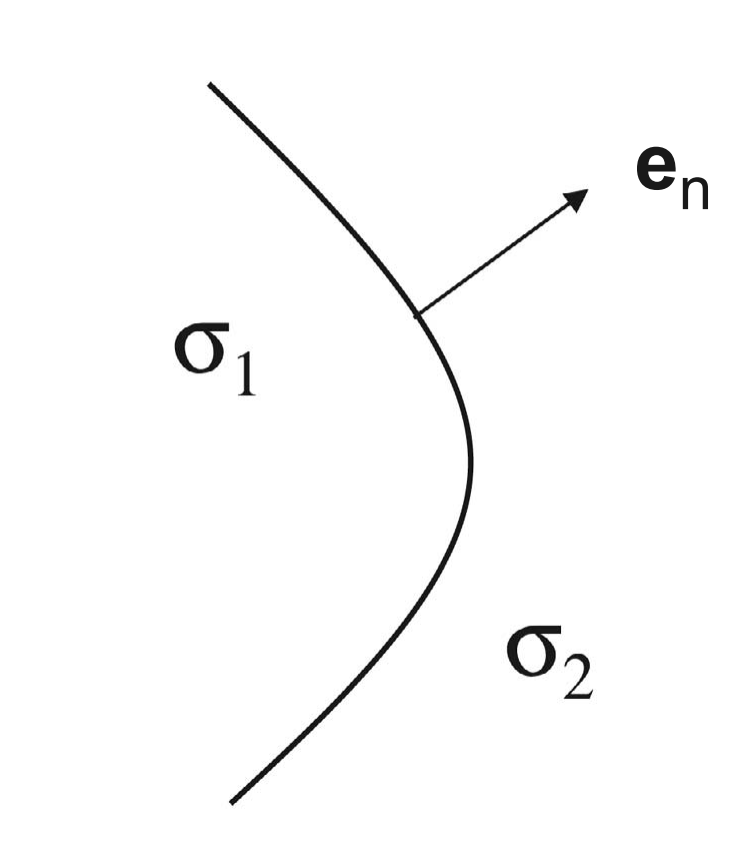
**The boundary between two compartments**. The boundary between two compartments. The boundary between two compartments, with conductivity *σ*_1 _and *σ*_2_. The normal vector **e**_*n *_to the interface is also shown.

At the interface between two compartments, two boundary conditions are found. Figure 5 illustrates such an interface. A first condition is based on the inability to pile up charge at the interface. All charge leaving one compartment through the interface must enter the other compartment. In other words, all current (charge per second) leaving a compartment with conductivity *σ*_1 _through the interface enters the neighboring compartment with conductivity *σ*_2_:

J1⋅en=J2⋅en,(σ1∇V1)⋅en=(σ2∇V2)⋅en,
 MathType@MTEF@5@5@+=feaafiart1ev1aaatCvAUfKttLearuWrP9MDH5MBPbIqV92AaeXatLxBI9gBaebbnrfifHhDYfgasaacPC6xNi=xI8qiVKYPFjYdHaVhbbf9v8qqaqFr0xc9vqFj0dXdbba91qpepeI8k8fiI+fsY=rqGqVepae9pg0db9vqaiVgFr0xfr=xfr=xc9adbaqaaeGacaGaaiaabeqaaeqabiWaaaGcbaqbaeWabiqaaaqaaGqabiab=PeaknaaBaaaleaacqWFXaqmaeqaaOGaeyyXICTae8xzau2aaSbaaSqaaiabd6gaUbqabaGccqGH9aqpcqWFkbGsdaWgaaWcbaGae8NmaidabeaakiabgwSixlab=vgaLnaaBaaaleaacqWGUbGBaeqaaOGaeiilaWcabaGaeiikaGccciGae43Wdm3aaSbaaSqaaiabigdaXaqabaGccqGHhis0cqWGwbGvdaWgaaWcbaGaeGymaedabeaakiabcMcaPiabgwSixlab=vgaLnaaBaaaleaacqWGUbGBaeqaaOGaeyypa0JaeiikaGIae43Wdm3aaSbaaSqaaiabikdaYaqabaGccqGHhis0cqWGwbGvdaWgaaWcbaGaeGOmaidabeaakiabcMcaPiabgwSixlab=vgaLnaaBaaaleaacqWGUbGBaeqaaOGaeiilaWcaaaaa@5A40@

where **e**_*n *_is the normal component on the interface.

In particular no current can be injected into the air outside the human head due to the very low conductivity of the air. Therefore the current density at the surface of the head reads:

J1⋅en=0,(σ1⋅∇V1)⋅en=0.
 MathType@MTEF@5@5@+=feaafiart1ev1aaatCvAUfKttLearuWrP9MDH5MBPbIqV92AaeXatLxBI9gBaebbnrfifHhDYfgasaacPC6xNi=xI8qiVKYPFjYdHaVhbbf9v8qqaqFr0xc9vqFj0dXdbba91qpepeI8k8fiI+fsY=rqGqVepae9pg0db9vqaiVgFr0xfr=xfr=xc9adbaqaaeGacaGaaiaabeqaaeqabiWaaaGcbaqbaeWabiqaaaqaaGqabiab=PeaknaaBaaaleaacqWFXaqmaeqaaOGaeyyXICTae8xzau2aaSbaaSqaaiabd6gaUbqabaGccqGH9aqpcqaIWaamcqGGSaalaeaacqGGOaakiiGacqGFdpWCdaWgaaWcbaGaeGymaedabeaakiabgwSixlabgEGirlabdAfawnaaBaaaleaacqaIXaqmaeqaaOGaeiykaKIaeyyXICTae8xzau2aaSbaaSqaaiabd6gaUbqabaGccqGH9aqpcqaIWaamcqGGUaGlaaaaaa@4950@

Equations (11) and (12) are called the Neumann boundary condition and the homogeneous Neumann boundary condition, respectively.

The second boundary condition only holds for interfaces not connected with air. By crossing the interface the potential cannot have discontinuities,

*V*_1 _= *V*_2_.

This equation represents the Dirichlet boundary condition.

### The current dipole

**Figure 6:**
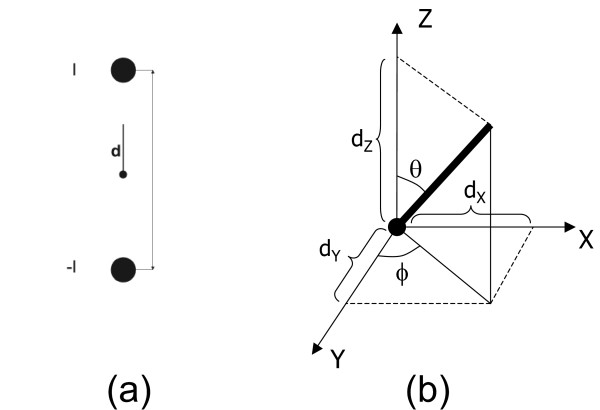
**The dipole parameters**. (a) The dipole parameters for a given current source and current sink configuration. (b) The dipole as a vector consisting of 6 parameters. 3 parameters are needed for the location of the dipole. 3 other parameters are needed for the vector components of the dipole. These vector components can also be transformed into spherical components: an azimuth, elevation and magnitude of the dipole.

Current source and current sink inject and remove the same amount of current *I *and they represent an active pyramidal cell at microscopic level. They can be modeled as a current dipole as illustrated in figure 6(a). The position parameter **r**_*dip *_of the dipole is typically chosen half way between the two monopoles.

The dipole moment **d **is defined by a unit vector **e**_**d **_(which is directed from the current sink to the current source) and a magnitude given by *d *= ||**d**|| = *I*·*p*, with *p *the distance between the two monopoles. Hence one can write:

**d **= *I*·*p***e**_**d**_.

It is often so that a dipole is decomposed in three dipoles located at the same position of the original dipole and each oriented along one of the Cartesian axes. The magnitude of each of these dipoles is equal to the orthogonal projection on the respective axis as illustrated in figure 6(b). one can write:

**d **= *d*_*x*_**e**_*x *_+ *d*_*y*_**e**_*y *_+ *d*_*z*_**e**_*z*_,

with **e**_*x*_, **e**_*y *_and **e**_*z *_being the unit vectors along the three axes. Furthermore, *d*_*x*_, *d*_*y *_and *d*_*z *_are often called the dipole components. Notice that Poisson's equation (8) is linear. Due to a dipole at a position **r**_*dip *_and dipole moment **d**, a potential *V *at an arbitrary scalp measurement point **r **can be decomposed in:

*V*(**r**, **r**_*dip*_, **d**) = *d*_*x*_*V*(**r**, **r**_*dip*_, **e**_*x*_) + *d*_*y*_*V*(**r**, **r**_*dip*_, **e**_*y*_) + *d*_*z*_*V*(**r**, **r**_*dip*_, **e**_*z*_).

A large group of pyramidal cells need to be more or less synchronously active in a cortical patch to have a measurable EEG signal. All these cells are furthermore oriented with their longitudinal axis orthogonal to the cortical surface. Due to this arrangement the superposition of the individual electrical activity of the neurons results in an amplification of the potential distribution. A large group of electrically active pyramidal cells in a small patch of cortex can be represented as one equivalent dipole on macroscopic level [36,37]. It is very difficult to estimate the extent of the active area of the cortex as the potential distribution on the scalp is almost identical to that of an equivalent dipole [38].

#### General algebraic formulation of the forward problem

In symbolic terms, the EEG forward problem is that of finding, in a reasonable time, the scalp potential *g*(**r**, **r**_*dip*_, **d**) at an electrode positioned on the scalp at **r **due to a single dipole with dipole moment **d **= *d***e**_**d **_(with magnitude *d *and orientation **e**_**d**_), positioned at **r**_*dip*_. This amounts to solving Poisson's equation to find the potentials *V*(**r**) on the scalp for different configurations of **r**_*dip *_and **d**. For multiple dipole sources, the electrode potential would be V(r)=∑ig(r,rdipi,di)=∑ig(r,rdipi,edi)di
 MathType@MTEF@5@5@+=feaafiart1ev1aaatCvAUfKttLearuWrP9MDH5MBPbIqV92AaeXatLxBI9gBaebbnrfifHhDYfgasaacPC6xNi=xH8viVGI8Gi=hEeeu0xXdbba9frFj0xb9qqpG0dXdb9aspeI8k8fiI+fsY=rqGqVepae9pg0db9vqaiVgFr0xfr=xfr=xc9adbaqaaeGacaGaaiaabeqaaeqabiWaaaGcbaGaemOvayLaeiikaGccbeGae8NCaiNaeiykaKIaeyypa0ZaaabuaeaacqWGNbWzcqGGOaakcqWFYbGCcqGGSaalcqWFYbGCdaWgaaWcbaGaemizaqMaemyAaKMaemiCaa3aaSbaaWqaaiabdMgaPbqabaaaleqaaOGaeiilaWIae8hzaq2aaSbaaSqaaiabdMgaPbqabaGccqGGPaqkcqGH9aqpaSqaaiabdMgaPbqab0GaeyyeIuoakmaaqafabaGaem4zaCMaeiikaGIae8NCaiNaeiilaWIae8NCai3aaSbaaSqaaiabdsgaKjabdMgaPjabdchaWnaaBaaameaacqWGPbqAaeqaaaWcbeaakiabcYcaSiab=vgaLnaaBaaaleaacqWFKbazdaWgaaadbaGaemyAaKgabeaaaSqabaGccqGGPaqkcqWGKbazdaWgaaWcbaGaemyAaKgabeaaaeaacqWGPbqAaeqaniabggHiLdaaaa@5E41@. In practice, one calculates a potential between an electrode and a reference (which can be another electrode or an average reference).

For *N *electrodes and *p *dipoles:

V=[V(r1)⋮V(rN)]=[g(r1,rdip1,ed1)⋯g(r1,rdipp,edp)⋮⋱⋮g(rN,rdip1,ed1)⋯g(rN,rdipp,edp)][d1⋮dp]=G({rj,rdipi,edi})[d1⋮dp]
 MathType@MTEF@5@5@+=feaafiart1ev1aaatCvAUfKttLearuWrP9MDH5MBPbIqV92AaeXatLxBI9gBaebbnrfifHhDYfgasaacPC6xNi=xI8qiVKYPFjYdHaVhbbf9v8qqaqFr0xc9vqFj0dXdbba91qpepeI8k8fiI+fsY=rqGqVepae9pg0db9vqaiVgFr0xfr=xfr=xc9adbaqaaeGacaGaaiaabeqaaeqabiWaaaGcbaacbeGae8NvayLaeyypa0ZaamWaaeaafaqabeWabaaabaGaemOvayLaeiikaGIae8NCai3aaSbaaSqaaiabigdaXaqabaGccqGGPaqkaeaacqWIUlstaeaacqWGwbGvcqGGOaakcqWFYbGCdaWgaaWcbaGaemOta4eabeaakiabcMcaPaaaaiaawUfacaGLDbaacqGH9aqpdaWadaqaauaabeqadmaaaeaacqWGNbWzcqGGOaakcqWFYbGCdaWgaaWcbaGaeGymaedabeaakiabcYcaSiab=jhaYnaaBaaaleaacqWGKbazcqWGPbqAcqWGWbaCdaWgaaadbaGaeGymaedabeaaaSqabaGccqGGSaalcqWFLbqzdaWgaaWcbaGae8hzaq2aaSbaaWqaaiabigdaXaqabaaaleqaaOGaeiykaKcabaGaeS47IWeabaGaem4zaCMaeiikaGIae8NCai3aaSbaaSqaaiabigdaXaqabaGccqGGSaalcqWFYbGCdaWgaaWcbaGaemizaqMaemyAaKMaemiCaa3aaSbaaWqaaiabdchaWbqabaaaleqaaOGaeiilaWIae8xzau2aaSbaaSqaaiab=rgaKnaaBaaameaacqWGWbaCaeqaaaWcbeaakiabcMcaPaqaaiabl6UinbqaaiablgVipbqaaiabl6UinbqaaiabdEgaNjabcIcaOiab=jhaYnaaBaaaleaacqWGobGtaeqaaOGaeiilaWIae8NCai3aaSbaaSqaaiabdsgaKjabdMgaPjabdchaWnaaBaaameaacqaIXaqmaeqaaaWcbeaakiabcYcaSiab=vgaLnaaBaaaleaacqWFKbazdaWgaaadbaGaeGymaedabeaaaSqabaGccqGGPaqkaeaacqWIVlctaeaacqWGNbWzcqGGOaakcqWFYbGCdaWgaaWcbaGaemOta4eabeaakiabcYcaSiab=jhaYnaaBaaaleaacqWGKbazcqWGPbqAcqWGWbaCdaWgaaadbaGaemiCaahabeaaaSqabaGccqGGSaalcqWFLbqzdaWgaaWcbaGae8hzaq2aaSbaaWqaaiabdchaWbqabaaaleqaaOGaeiykaKcaaaGaay5waiaaw2faamaadmaabaqbaeqabmqaaaqaaiabdsgaKnaaBaaaleaacqaIXaqmaeqaaaGcbaGaeSO7I0eabaGaemizaq2aaSbaaSqaaiabdchaWbqabaaaaaGccaGLBbGaayzxaaGaeyypa0Jae83raCKaeiikaGIaei4EaSNae8NCai3aaSbaaSqaaiabdQgaQbqabaGccqGGSaalcqWFYbGCdaWgaaWcbaGaemizaqMaemyAaKMaemiCaa3aaSbaaWqaaiabdMgaPbqabaaaleqaaOGaeiilaWIae8xzau2aaSbaaSqaaiab=rgaKnaaBaaameaacqWGPbqAaeqaaaWcbeaakiabc2ha9jabcMcaPmaadmaabaqbaeqabmqaaaqaaiabdsgaKnaaBaaaleaacqaIXaqmaeqaaaGcbaGaeSO7I0eabaGaemizaq2aaSbaaSqaaiabdchaWbqabaaaaaGccaGLBbGaayzxaaaaaa@BF11@

where *i *= 1,...,*p *and *j *= 1,...,*N*. Here **V **is a column vector.

For *N *electrodes, *p *dipoles and *T *discrete time samples:

V=[V(r1,1)⋯V(r1,T)⋮⋱⋮V(rN,1)⋯V(rN,T)]=G({rj,rdipi,edi})[d1,1⋯d1,T⋮⋱⋮dp,1⋯dp,T]=G({rj,rdipi,edi})D
 MathType@MTEF@5@5@+=feaafiart1ev1aaatCvAUfKttLearuWrP9MDH5MBPbIqV92AaeXatLxBI9gBaebbnrfifHhDYfgasaacPC6xNi=xI8qiVKYPFjYdHaVhbbf9v8qqaqFr0xc9vqFj0dXdbba91qpepeI8k8fiI+fsY=rqGqVepae9pg0db9vqaiVgFr0xfr=xfr=xc9adbaqaaeGacaGaaiaabeqaaeqabiWaaaGcbaqbaeqabiqaaaqaaGqabiab=zfawjabg2da9maadmaabaqbaeqabmWaaaqaaiabdAfawjabcIcaOiab=jhaYnaaBaaaleaacqaIXaqmaeqaaOGaeiilaWIaeGymaeJaeiykaKcabaGaeS47IWeabaGaemOvayLaeiikaGIae8NCai3aaSbaaSqaaiabigdaXaqabaGccqGGSaalcqWGubavcqGGPaqkaeaacqWIUlstaeaacqWIXlYtaeaacqWIUlstaeaacqWGwbGvcqGGOaakcqWFYbGCdaWgaaWcbaGaemOta4eabeaakiabcYcaSiabigdaXiabcMcaPaqaaiabl+UimbqaaiabdAfawjabcIcaOiab=jhaYnaaBaaaleaacqWGobGtaeqaaOGaeiilaWIaemivaqLaeiykaKcaaaGaay5waiaaw2faaaqaaiabg2da9iab=DeahjabcIcaOiabcUha7jab=jhaYnaaBaaaleaacqWGQbGAaeqaaOGaeiilaWIae8NCai3aaSbaaSqaaiabdsgaKjabdMgaPjabdchaWnaaBaaameaacqWGPbqAaeqaaaWcbeaakiabcYcaSiab=vgaLnaaBaaaleaacqWFKbazdaWgaaadbaGaemyAaKgabeaaaSqabaGccqGG9bqFcqGGPaqkdaWadaqaauaabeqadmaaaeaacqWGKbazdaWgaaWcbaGaeGymaeJaeiilaWIaeGymaedabeaaaOqaaiabl+UimbqaaiabdsgaKnaaBaaaleaacqaIXaqmcqGGSaalcqWGubavaeqaaaGcbaGaeSO7I0eabaGaeSy8I8eabaGaeSO7I0eabaGaemizaq2aaSbaaSqaaiabdchaWjabcYcaSiabigdaXaqabaaakeaacqWIVlctaeaacqWGKbazdaWgaaWcbaGaemiCaaNaeiilaWIaemivaqfabeaaaaaakiaawUfacaGLDbaacqGH9aqpcqWFhbWrcqGGOaakcqGG7bWEcqWFYbGCdaWgaaWcbaGaemOAaOgabeaakiabcYcaSiab=jhaYnaaBaaaleaacqWGKbazcqWGPbqAcqWGWbaCdaWgaaadbaGaemyAaKgabeaaaSqabaGccqGGSaalcqWFLbqzdaWgaaWcbaGae8hzaq2aaSbaaWqaaiabdMgaPbqabaaaleqaaOGaeiyFa0NaeiykaKIae8hraqeaaaaa@A5DE@

where **V **is now the matrix of data measurements, **G **is the gain matrix and **D **is the matrix of dipole magnitudes at different time instants.

More generally, a noise or perturbation matrix **n **is added,

**V **= **GD **+ **n**.

In general for simulations and to measure noise sensitivity, noise distribution is a gaussian distribution with zero mean and variable standard deviation. However in reality, the noise is coloured and the distribution of the frequency depends on a lot of factors: patient, measurement setup, pathology,....

#### A general multipole expansion of the source model

Solving the inverse problem using multiple dipole model requires the estimation of a large number of parameters, 6 for each dipole. Given the use of a limited number of EEG electrodes, the problem becomes underdetermined. In this case, regularization techniques have to be applied, but this leads to oversmoothed source estimations. On the other hand, the use of a limited number of dipoles (one, two or three) leads to very simplified sources, which are very often ambiguous and cause errors due to simplified modelling. The dipole model as a source is a good model for focal brain activity.

A multipole expansion is an alternative (first introduced by [39]), which is based on a spherical harmonic expansion of the volume source, which is not necessarily focal. It provides the added model flexibility needed to account for a wide range of physiologically plausible sources, while at the same time keeping the number of estimation parameters sufficiently low. In fact, The zeroth-order and first-order terms in the expansion are called the monopole and dipole moment, respectively. A quadrupole is a higher order term and is generated by two equal and oppositely oriented dipoles whose moments tend to infinity as they are brought infinitesimally close to each other. An octapole consists of two quadrupoles brought infinitesimally close to each other and so on. It can be shown that if the volume *G *containing the active sources *I*_*sv*_(**r***'*) is limited in extent, the solution to Poisson's equation for the potential *V *may be expanded in terms of a multipole series:

*V *= *V*_*monopole *_+ *V*_*dipole *_+ *V*_*quadrupole *_+ *V*_*octapole *_+ *V*_*hexadecpole *_+ ...

where *V*_*quadrupole *_is the potential field caused by the quadrupole. In practice, a truncated multipole series is used up to a quadrupole, because the contribution to the electrode potentials by a octapole or higher order sources rapidly decreases when the distance between electrode and source is increasing. The use of quadrupoles can sound plausible in the following case: A traveling action potential causes a depolarization wave through the axon, followed by a repolarization wave. These two phenomenon produce two opposite oriented dipoles very close to each other [40]. In sulci, pyramidal cells are oriented toward each other, which makes the use of quadrupole also reasonable. However, the skull causes a strong attenuation of the electrical field created by the source. Therefore, even a quadrupole has low contribution to the electrode potentials of the EEG, created by the volume current in the extracellular region. In EEG and ECG multiple dipoles of dipole layers are preferred over a multipole. Multipoles are popular in magnetoencephalographic (MEG) source localization, because of its low sensitivity to the skull conductivity [6,10,41,42].

## Solving the forward problem

### Dipole field in an infinite homogeneous isotropic conductor

**Figure 7:**
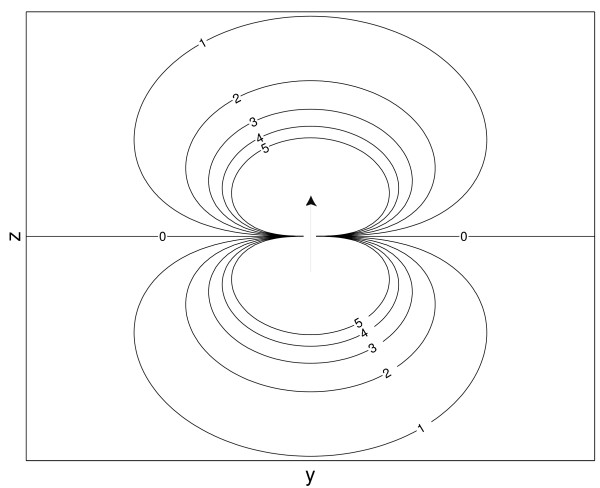
**The equipotential lines of a dipole**. The equipotential lines of a dipole oriented along the *z*-axis. The numbers correspond to the level of intensity of the potential field generated of the dipole. The zero line divides the dipole field into two parts: a positive one and a negative one.

The potential field generated by a current dipole with dipole moment **d **= *d***e**_**d **_at a position **r**_*dip *_in an infinite conductor with conductivity *σ*, is introduced. The potential field is given by:

V(r,rdip,d)=d⋅(r−rdip)4πσ||r−rdip||3,
 MathType@MTEF@5@5@+=feaafiart1ev1aaatCvAUfKttLearuWrP9MDH5MBPbIqV92AaeXatLxBI9gBaebbnrfifHhDYfgasaacPC6xNi=xI8qiVKYPFjYdHaVhbbf9v8qqaqFr0xc9vqFj0dXdbba91qpepeI8k8fiI+fsY=rqGqVepae9pg0db9vqaiVgFr0xfr=xfr=xc9adbaqaaeGacaGaaiaabeqaaeqabiWaaaGcbaGaemOvayLaeiikaGccbeGae8NCaiNaeiilaWIae8NCai3aaSbaaSqaaiabdsgaKjabdMgaPjabdchaWbqabaGccqGGSaalcqWFKbazcqGGPaqkcqGH9aqpjuaGdaWcaaqaaiab=rgaKjabgwSixlabcIcaOiab=jhaYjabgkHiTiab=jhaYnaaBaaabaGaemizaqMaemyAaKMaemiCaahabeaacqGGPaqkaeaacqaI0aaniiGacqGFapaCcqGFdpWCcqGG8baFcqGG8baFcqWFYbGCcqGHsislcqWFYbGCdaWgaaqaaiabdsgaKjabdMgaPjabdchaWbqabaGaeiiFaWNaeiiFaW3aaWbaaeqabaGaeG4mamdaaaaakiabcYcaSaaa@5C89@

with **r **being the position where the potential is calculated. Assume that the dipole is located in the origin of the Cartesian coordinate system and oriented along the *z*-axis. Then it can be written:

V(r,0,dez)=dcos⁡θ4πσr2,
 MathType@MTEF@5@5@+=feaafiart1ev1aaatCvAUfKttLearuWrP9MDH5MBPbIqV92AaeXatLxBI9gBaebbnrfifHhDYfgasaacPC6xNi=xI8qiVKYPFjYdHaVhbbf9v8qqaqFr0xc9vqFj0dXdbba91qpepeI8k8fiI+fsY=rqGqVepae9pg0db9vqaiVgFr0xfr=xfr=xc9adbaqaaeGacaGaaiaabeqaaeqabiWaaaGcbaGaemOvayLaeiikaGccbeGae8NCaiNaeiilaWIae8hmaaJaeiilaWIaemizaqMae8xzau2aaSbaaSqaaiabdQha6bqabaGccqGGPaqkcqGH9aqpjuaGdaWcaaqaaiabdsgaKjGbcogaJjabc+gaVjabcohaZHGaciab+H7aXbqaaiabisda0iab+b8aWjab+n8aZjabdkhaYnaaCaaabeqaaiabikdaYaaaaaGccqGGSaalaaa@481D@

where *θ *represents the angle between the *z*-axis and **r **and *r *= ||**r**||. An illustration of the electrical potential field caused by dipole is shown in figure 7.

Equation (20) shows that a dipole field attenuates with 1/*r*^2^. It is significant to remark that *V*, from equation (19), added with an arbitrary constant, is also a solution of Poisson's equation. A reference potential must be chosen. One can choose to set one electrode to zero or one can opt for average referenced potentials. The latter result in electrode potentials that have a zero mean.

### The spherical head model

**Figure 8:**
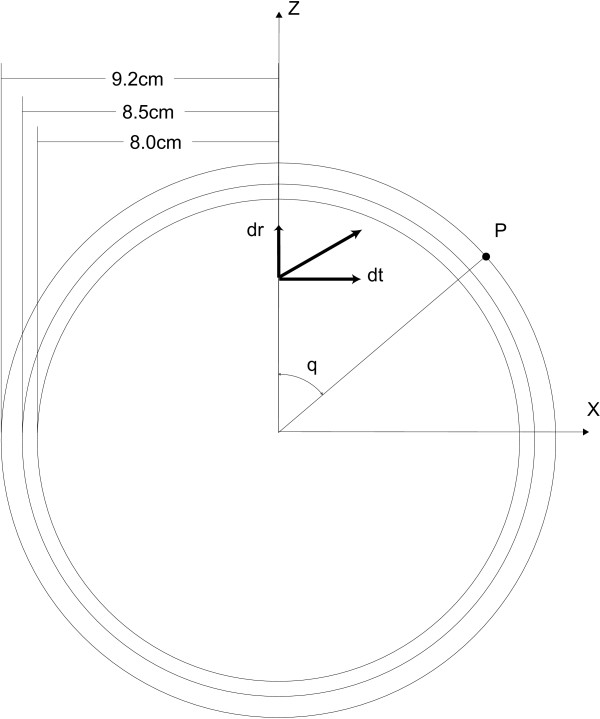
**The three-shell concentric spherical head model**. The dipole is located on the *z*-axis and the potential is measured at scalp point *P *located in the *xz*-plane.

The first volume conductor models of the human head consisted of a homogeneous sphere [43]. However it was soon noticed that the skull tissue had a conductivity which was significantly lower than the conductivity of scalp and brain tissue. Therefore the volume conductor model of the head needed further refinement and a three-shell concentric spherical head model was introduced. In this model, the inner sphere represents the brain, the intermediate layer represents the skull and the outer layer represents the scalp. For this geometry a semi-analytical solution of Poisson's equation exists. The derivation is based on [44,45]. Consider a dipole located on the *z*-axis and a scalp point *P*, located in the *xz*-plane, as illustrated in figure 8. The dipole components located in the *xz*-plane i.e. *d*_*r *_the radial component and *d*_*t *_the tangential component, are also shown in figure 8. The component orthogonal to the *xz*-plane, does not contribute to the potential at scalp point *P *due to the fact that the zero potential plane of this component traverses *P*. The potential *V *at scalp point *P *for the proposed dipole is given by:

V=14πSR2∑i=1∞X(2i+1)3gi(i+1)ibi−1[idrPi(cos⁡θ)+dtPi1(cos⁡θ)],
 MathType@MTEF@5@5@+=feaafiart1ev1aaatCvAUfKttLearuWrP9MDH5MBPbIqV92AaeXatLxBI9gBaebbnrfifHhDYfgasaacPC6xNi=xI8qiVKYPFjYdHaVhbbf9v8qqaqFr0xc9vqFj0dXdbba91qpepeI8k8fiI+fsY=rqGqVepae9pg0db9vqaiVgFr0xfr=xfr=xc9adbaqaaeGacaGaaiaabeqaaeqabiWaaaGcbaGaemOvayLaeyypa0tcfa4aaSaaaeaacqaIXaqmaeaacqaI0aaniiGacqWFapaCcqWGtbWucqWGsbGudaahaaqabeaacqaIYaGmaaaaaOWaaabCaeaajuaGdaWcaaqaaiabdIfayjabcIcaOiabikdaYiabdMgaPjabgUcaRiabigdaXiabcMcaPmaaCaaabeqaaiabiodaZaaaaeaacqWGNbWzdaWgaaqaaiabdMgaPbqabaGaeiikaGIaemyAaKMaey4kaSIaeGymaeJaeiykaKIaemyAaKgaaOGaemOyai2aaWbaaSqabeaacqWGPbqAcqGHsislcqaIXaqmaaGccqGGBbWwcqWGPbqAcqWGKbazdaWgaaWcbaGaemOCaihabeaakiabdcfaqnaaBaaaleaacqWGPbqAaeqaaOGaeiikaGIagi4yamMaei4Ba8Maei4CamNae8hUdeNaeiykaKIaey4kaSIaemizaq2aaSbaaSqaaiabdsha0bqabaGccqWGqbaudaqhaaWcbaGaemyAaKgabaGaeGymaedaaOGaeiikaGIagi4yamMaei4Ba8Maei4CamNae8hUdeNaeiykaKIaeiyxa0LaeiilaWcaleaacqWGPbqAcqGH9aqpcqaIXaqmaeaacqGHEisPa0GaeyyeIuoaaaa@74AF@

with *g*_*i *_given by:

gi=[(i+1)X+i][iXi+1+1]+(1−X)[(i+1)X+i](f1i1−f2i1)−i(1−X)2(f1/f2)i1.
 MathType@MTEF@5@5@+=feaafiart1ev1aaatCvAUfKttLearuWrP9MDH5MBPbIqV92AaeXatLxBI9gBaebbnrfifHhDYfgasaacPC6xNi=xI8qiVKYPFjYdHaVhbbf9v8qqaqFr0xc9vqFj0dXdbba91qpepeI8k8fiI+fsY=rqGqVepae9pg0db9vqaiVgFr0xfr=xfr=xc9adbaqaaeGacaGaaiaabeqaaeqabiWaaaGcbaGaem4zaC2aaSbaaSqaaiabdMgaPbqabaGccqGH9aqpcqGGBbWwcqGGOaakcqWGPbqAcqGHRaWkcqaIXaqmcqGGPaqkcqWGybawcqGHRaWkcqWGPbqAcqGGDbqxcqGGBbWwjuaGdaWcaaqaaiabdMgaPjabdIfaybqaaiabdMgaPjabgUcaRiabigdaXaaakiabgUcaRiabigdaXiabc2faDjabgUcaRiabcIcaOiabigdaXiabgkHiTiabdIfayjabcMcaPiabcUfaBjabcIcaOiabdMgaPjabgUcaRiabigdaXiabcMcaPiabdIfayjabgUcaRiabdMgaPjabc2faDjabcIcaOiabdAgaMnaaDaaaleaacqaIXaqmaeaacqWGPbqAdaWgaaadbaGaeGymaedabeaaaaGccqGHsislcqWGMbGzdaqhaaWcbaGaeGOmaidabaGaemyAaK2aaSbaaWqaaiabigdaXaqabaaaaOGaeiykaKIaeyOeI0IaemyAaKMaeiikaGIaeGymaeJaeyOeI0IaemiwaGLaeiykaKYaaWbaaSqabeaacqaIYaGmaaGccqGGOaakcqWGMbGzdaWgaaWcbaGaeGymaedabeaakiabc+caViabdAgaMnaaBaaaleaacqaIYaGmaeqaaOGaeiykaKYaaWbaaSqabeaacqWGPbqAdaWgaaadbaGaeGymaedabeaaaaGccqGGUaGlaaa@7603@

Where:

*d*_*r *_is the radial component (3 × 1-vector in meters),

*d*_*t *_is the tangential component (3 × 1-vector in meters),

*R *is the radius of the outer shell (meters),

*S *is the conductivity of scalp and brain tissue (Siemens/meter),

*X *is the ratio between the skull and soft tissue conductivity (unitless),

*b *is the relative distance of the dipole from the centre (unitless),

*θ *is the polar angle of the surface point see figure 8 (radians),

*P*_*i*_(·) is the Legendre polynomial,

Pi1(⋅)
 MathType@MTEF@5@5@+=feaafiart1ev1aaatCvAUfKttLearuWrP9MDH5MBPbIqV92AaeXatLxBI9gBaebbnrfifHhDYfgasaacPC6xNi=xH8viVGI8Gi=hEeeu0xXdbba9frFj0xb9qqpG0dXdb9aspeI8k8fiI+fsY=rqGqVepae9pg0db9vqaiVgFr0xfr=xfr=xc9adbaqaaeGacaGaaiaabeqaaeqabiWaaaGcbaGaemiuaa1aa0baaSqaaiabdMgaPbqaaiabigdaXaaakiabcIcaOiabgwSixlabcMcaPaaa@337A@ is the associated Legendre polynomial,

*i *is an index,

*i*_1 _equals 2*i *+ 1,

*r*_1 _is the radius of the inner shell (in meters),

*r*_2 _is the radius of the middle shell (in meters),

*f*_1 _equals *r*_1_/*R *(unitless) and

*f*_2 _equals *r*_2_/*R *(unitless).

Equation (21) gives the scalp potentials generated by a dipole located on the *z*-axis, with zero dipole moment in the *y *direction. To find the scalp potentials generated by an arbitrary dipole, the coordinate system has to be rotated accordingly. Typical radii of the outer boundaries of the brain, skull and scalp compartments are equal to 8 cm, 8.5 cm and 9.2 cm, respectively [46]. An illustration of a typical spherical head model is shown in figure 8. These radii can be altered to fit a sphere more to the human head. The infinite series of equation (21) is often truncated. If the first 40 terms are used, the maximum scalp potential obtained with the truncated series, deviates less than 0.1% from the case where 100 terms are applied, for dipoles with a radial position smaller than 95% of the maximum brain radius.

There are also semi-analytical solutions available for layered spheroidal anisotropic volume conductors [47-49]. Here the conductivity in the tangential direction can be chosen differently than in the radial direction of the sphere. Analytic solutions also exist for prolate and oblate spheroids or eccentric spheres [50-52].

Variants of the three-shell spherical head model, such as the Berg approximation [53], in which a single-sphere model is used to approximate a three- (or four-) layer sphere, have also been used to improve further the computational efficiency of multi-layer spherical models.

Recently however, it is becoming more apparent that the actual geometry of the head [54-56] together with the varying thickness and curvatures of the skull [57,58], affects the solutions appreciably. So-called real head models are becoming much more common in the literature, in conjunction with either boundary-element, finite-element, or finite-difference methods. However, the computational requirements for a realistic head model are higher than that for a multi-layer sphere.

An approach which is situated between the spherical head model approaches and realistic ones is the sensor-fitted sphere approach [59]. Here a multilayer sphere is fitted to each sensor located on the surface of a realistic head model.

### The boundary element method

The boundary element method (BEM) is a numerical technique for calculating the surface potentials generated by current sources located in a piecewise homogeneous volume conductor. Although it restricts us to use only isotropic conductivities, it is still widely used because of its low computational needs. The method originated in the field of electrocardiography in the late sixties and made its entrance in the field of EEG source localization in the late eighties [60]. As the name implies, this method is capable of providing a solution to a volume problem by calculating the potential values at the interfaces and boundary of the volume induced by a given current source (e.g. a dipole). The interfaces separate regions of differing conductivity within the volume, while the boundary is the outer surface seperating the non-conducting air with the conducting volume.

In practice, a head model is built from surfaces, each encapsulating a particular tissue. Typically, head models consist of 3 surfaces: brain-skull interface, skull-scalp interface and the outer surface. The regions between the interfaces are assumed to be homogeneous and isotropic conducting. To obtain a solution in such a piecewise homogenous volume, each interface is tesselated with small boundary elements.

The integral equations describing the potential *V*(**r**) at any point **r **in a piecewise volume conductor *V *were described in [61-63]:

V(r)=2σ0σk−+σk+V0(r)+12π∑j=1Rσj−_σj+σk−+σk+∫r′∈SjV(r′)r′−r||r′−r||3dSj,
 MathType@MTEF@5@5@+=feaafiart1ev1aaatCvAUfKttLearuWrP9MDH5MBPbIqV92AaeXatLxBI9gBaebbnrfifHhDYfgasaacPC6xNi=xI8qiVKYPFjYdHaVhbbf9v8qqaqFr0xc9vqFj0dXdbba91qpepeI8k8fiI+fsY=rqGqVepae9pg0db9vqaiVgFr0xfr=xfr=xc9adbaqaaeGacaGaaiaabeqaaeqabiWaaaGcbaGaemOvayLaeiikaGccbeGae8NCaiNaeiykaKIaeyypa0tcfa4aaSaaaeaacqaIYaGmiiGacqGFdpWCdaWgaaqaaiabicdaWaqabaaabaGae43Wdm3aa0baaeaacqWGRbWAaeaacqGHsislaaGaey4kaSIae43Wdm3aa0baaeaacqWGRbWAaeaacqGHRaWkaaaaaOGaemOvay1aaSbaaSqaaiabicdaWaqabaGccqGGOaakcqWFYbGCcqGGPaqkcqGHRaWkjuaGdaWcaaqaaiabigdaXaqaaiabikdaYiab+b8aWbaakmaaqahabaqcfa4aaSaaaeaacqGFdpWCdaqhaaqaaiabdQgaQbqaaiabgkHiTaaacqGGFbWxcqGFdpWCdaqhaaqaaiabdQgaQbqaaiabgUcaRaaaaeaacqGFdpWCdaqhaaqaaiabdUgaRbqaaiabgkHiTaaacqGHRaWkcqGFdpWCdaqhaaqaaiabdUgaRbqaaiabgUcaRaaaaaGcdaWdraqaaiabdAfawjabcIcaOiqb=jhaYzaafaGaeiykaKscfa4aaSaaaeaacuWFYbGCgaqbaiabgkHiTiab=jhaYbqaaiabcYha8jabcYha8jqb=jhaYzaafaGaeyOeI0Iae8NCaiNaeiiFaWNaeiiFaW3aaWbaaeqabaGaeG4mamdaaaaakiabdsgaKjab=nfatnaaBaaaleaacqWFQbGAaeqaaaqaaiqb=jhaYzaafaGaeyicI4Saem4uam1aaSbaaWqaaiabdQgaQbqabaaaleqaniabgUIiYdaaleaacqWGQbGAcqGH9aqpcqaIXaqmaeaacqWGsbGua0GaeyyeIuoakiabcYcaSaaa@8432@

where *σ*_0 _corresponds to the medium in which the dipole source is located (the brain compartment) and *V*_0_(**r**) is the potential at **r **for an infinite medium with conductivity *σ*_0 _as in equation (19). σj−
 MathType@MTEF@5@5@+=feaafiart1ev1aaatCvAUfKttLearuWrP9MDH5MBPbIqV92AaeXatLxBI9gBaebbnrfifHhDYfgasaacPC6xNi=xH8viVGI8Gi=hEeeu0xXdbba9frFj0xb9qqpG0dXdb9aspeI8k8fiI+fsY=rqGqVepae9pg0db9vqaiVgFr0xfr=xfr=xc9adbaqaaeGacaGaaiaabeqaaeqabiWaaaGcbaacciGae83Wdm3aa0baaSqaaiabdQgaQbqaaiabgkHiTaaaaaa@3014@ and σj+
 MathType@MTEF@5@5@+=feaafiart1ev1aaatCvAUfKttLearuWrP9MDH5MBPbIqV92AaeXatLxBI9gBaebbnrfifHhDYfgasaacPC6xNi=xH8viVGI8Gi=hEeeu0xXdbba9frFj0xb9qqpG0dXdb9aspeI8k8fiI+fsY=rqGqVepae9pg0db9vqaiVgFr0xfr=xfr=xc9adbaqaaeGacaGaaiaabeqaaeqabiWaaaGcbaacciGae83Wdm3aa0baaSqaaiabdQgaQbqaaiabgUcaRaaaaaa@3009@ are the conductivities of the, respectively, inner and outer compartments divided by the interface *S*_*j*_. *d***S **is a vector oriented orthogonal to a surface element and ||*d***S**|| the area of that surface element.

**Figure 9:**
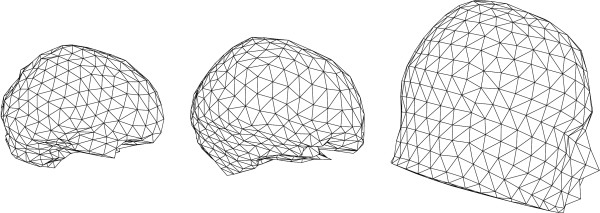
**Example mesh of the human head used in BEM**. Triangulated surfaces of the brain, skull and scalp compartment used in BEM. The surfaces indicate the different interfaces of the human head: air-scalp, scalp-skull and skull-brain.

Each interface *S*_*j *_is digitized in NSj
 MathType@MTEF@5@5@+=feaafiart1ev1aaatCvAUfKttLearuWrP9MDH5MBPbIqV92AaeXatLxBI9gBaebbnrfifHhDYfgasaacPC6xNi=xH8viVGI8Gi=hEeeu0xXdbba9frFj0xb9qqpG0dXdb9aspeI8k8fiI+fsY=rqGqVepae9pg0db9vqaiVgFr0xfr=xfr=xc9adbaqaaeGacaGaaiaabeqaaeqabiWaaaGcbaGaemOta40aaSbaaSqaaiabdofatnaaBaaameaacqWGQbGAaeqaaaWcbeaaaaa@2FE8@ triangles, (see figure 9) and in each triangle centre the potentials are calculated using equation (23). The integral over the surface *S*_*j *_is transformed into a summation of integrals over traingles on that surface. The potential values on surface *S*_*j *_can be written as

V(r)=2σ0σr−+σr+V0(r)+12π∑k=1Rσk−_σk+σr−+σr+∑j=1NSk∫ΔSk,jV(r′)r′−r||r′−r||3dSk,
 MathType@MTEF@5@5@+=feaafiart1ev1aaatCvAUfKttLearuWrP9MDH5MBPbIqV92AaeXatLxBI9gBaebbnrfifHhDYfgasaacPC6xNi=xI8qiVKYPFjYdHaVhbbf9v8qqaqFr0xc9vqFj0dXdbba91qpepeI8k8fiI+fsY=rqGqVepae9pg0db9vqaiVgFr0xfr=xfr=xc9adbaqaaeGacaGaaiaabeqaaeqabiWaaaGcbaGaemOvayLaeiikaGccbeGae8NCaiNaeiykaKIaeyypa0tcfa4aaSaaaeaacqaIYaGmiiGacqGFdpWCdaWgaaqaaiabicdaWaqabaaabaGae43Wdm3aa0baaeaacqWGYbGCaeaacqGHsislaaGaey4kaSIae43Wdm3aa0baaeaacqWGYbGCaeaacqGHRaWkaaaaaOGaemOvay1aaSbaaSqaaiabicdaWaqabaGccqGGOaakcqWFYbGCcqGGPaqkcqGHRaWkjuaGdaWcaaqaaiabigdaXaqaaiabikdaYiab+b8aWbaakmaaqahabaqcfa4aaSaaaeaacqGFdpWCdaqhaaqaaiabdUgaRbqaaiabgkHiTaaacqGGFbWxcqGFdpWCdaqhaaqaaiabdUgaRbqaaiabgUcaRaaaaeaacqGFdpWCdaqhaaqaaiabdkhaYbqaaiabgkHiTaaacqGHRaWkcqGFdpWCdaqhaaqaaiabdkhaYbqaaiabgUcaRaaaaaGcdaaeWbqaamaapebabaGaemOvayLaeiikaGIaf8NCaiNbauaacqGGPaqkjuaGdaWcaaqaaiqb=jhaYzaafaGaeyOeI0Iae8NCaihabaGaeiiFaWNaeiiFaWNaf8NCaiNbauaacqGHsislcqWFYbGCcqGG8baFcqGG8baFdaahaaqabeaacqaIZaWmaaaaaOGaemizaqMae83uam1aaSbaaSqaaiab=TgaRbqabaaabaGaeuiLdq0aaSbaaWqaaiabdofatnaaBaaabaGaem4AaSgabeaacqGGSaalcqWGQbGAaeqaaaWcbeqdcqGHRiI8aaWcbaGaemOAaOMaeyypa0JaeGymaedabaGaemOta40aaSbaaWqaaiabdofatnaaBaaabaGaem4AaSgabeaaaeqaaaqdcqGHris5aaWcbaGaem4AaSMaeyypa0JaeGymaedabaGaemOuaifaniabggHiLdGccqGGSaalaaa@8ED5@

where the integral is over ΔSj,k
 MathType@MTEF@5@5@+=feaafiart1ev1aaatCvAUfKttLearuWrP9MDH5MBPbIqV92AaeXatLxBI9gBaebbnrfifHhDYfgasaacPC6xNi=xH8viVGI8Gi=hEeeu0xXdbba9frFj0xb9qqpG0dXdb9aspeI8k8fiI+fsY=rqGqVepae9pg0db9vqaiVgFr0xfr=xfr=xc9adbaqaaeGacaGaaiaabeqaaeqabiWaaaGcbaGaeuiLdq0aaSbaaSqaaiabdofatnaaBaaameaacqWGQbGAaeqaaSGaeiilaWIaem4AaSgabeaaaaa@3268@, the *j*-th triangle on the surface *S*_*j*_, R is the number of interfaces in the volume. An exact solution of the integral is generally not possible, therefore an approximated solution V˜k(r)
 MathType@MTEF@5@5@+=feaafiart1ev1aaatCvAUfKttLearuWrP9MDH5MBPbIqV92AaeXatLxBI9gBaebbnrfifHhDYfgasaacPC6xNi=xH8viVGI8Gi=hEeeu0xXdbba9frFj0xb9qqpG0dXdb9aspeI8k8fiI+fsY=rqGqVepae9pg0db9vqaiVgFr0xfr=xfr=xc9adbaqaaeGacaGaaiaabeqaaeqabiWaaaGcbaGafmOvayLbaGaadaahaaWcbeqaaiabdUgaRbaakiabcIcaOGqabiab=jhaYjabcMcaPaaa@31D2@ on surface *S*_*k *_may be defined as a linear combination of NSk
 MathType@MTEF@5@5@+=feaafiart1ev1aaatCvAUfKttLearuWrP9MDH5MBPbIqV92AaeXatLxBI9gBaebbnrfifHhDYfgasaacPC6xNi=xH8viVGI8Gi=hEeeu0xXdbba9frFj0xb9qqpG0dXdb9aspeI8k8fiI+fsY=rqGqVepae9pg0db9vqaiVgFr0xfr=xfr=xc9adbaqaaeGacaGaaiaabeqaaeqabiWaaaGcbaGaemOta40aaSbaaSqaaiabdofatnaaBaaameaacqWGRbWAaeqaaaWcbeaaaaa@2FEA@ simple basis functions

Vk˜(r)=∑i=1NSkVikhi(r).
 MathType@MTEF@5@5@+=feaafiart1ev1aaatCvAUfKttLearuWrP9MDH5MBPbIqV92AaeXatLxBI9gBaebbnrfifHhDYfgasaacPC6xNi=xI8qiVKYPFjYdHaVhbbf9v8qqaqFr0xc9vqFj0dXdbba91qpepeI8k8fiI+fsY=rqGqVepae9pg0db9vqaiVgFr0xfr=xfr=xc9adbaqaaeGacaGaaiaabeqaaeqabiWaaaGcbaWaaacaaeaacqWGwbGvdaahaaWcbeqaaiabdUgaRbaaaOGaay5adaGaeiikaGccbeGae8NCaiNaeiykaKIaeyypa0ZaaabCaeaacqWGwbGvdaqhaaWcbaGaemyAaKgabaGaem4AaSgaaOGaemiAaG2aaSbaaSqaaiabdMgaPbqabaGccqGGOaakcqWFYbGCcqGGPaqkcqGGUaGlaSqaaiabdMgaPjabg2da9iabigdaXaqaaiabd6eaonaaBaaameaacqWGtbWudaWgaaqaaiabdUgaRbqabaaabeaaa0GaeyyeIuoaaaa@487C@

The coefficients Vik
 MathType@MTEF@5@5@+=feaafiart1ev1aaatCvAUfKttLearuWrP9MDH5MBPbIqV92AaeXatLxBI9gBaebbnrfifHhDYfgasaacPC6xNi=xH8viVGI8Gi=hEeeu0xXdbba9frFj0xb9qqpG0dXdb9aspeI8k8fiI+fsY=rqGqVepae9pg0db9vqaiVgFr0xfr=xfr=xc9adbaqaaeGacaGaaiaabeqaaeqabiWaaaGcbaGaemOvay1aa0baaSqaaiabdMgaPbqaaiabdUgaRbaaaaa@2FEF@ represent unknowns on surface *S*_*k *_whose values are determined by constraining V˜(x)
 MathType@MTEF@5@5@+=feaafiart1ev1aaatCvAUfKttLearuWrP9MDH5MBPbIqV92AaeXatLxBI9gBaebbnrfifHhDYfgasaacPC6xNi=xH8viVGI8Gi=hEeeu0xXdbba9frFj0xb9qqpG0dXdb9aspeI8k8fiI+fsY=rqGqVepae9pg0db9vqaiVgFr0xfr=xfr=xc9adbaqaaeGacaGaaiaabeqaaeqabiWaaaGcbaGafmOvayLbaGaacqGGOaakieqacqWF4baEcqGGPaqkaaa@3048@ to satisfy (24) at discrete points, also known as collocation points. Moreover, equation (24) can be rewritten as

V(r)=2σ0σr−+σr+V0(r)+12π∑k=1Rσk−_σk+σr−+σr+∑j=1NSk∑i=1NSkVik∫ΔSk,jhi(r)r′−r||r′−r||3dSk.
 MathType@MTEF@5@5@+=feaafiart1ev1aaatCvAUfKttLearuWrP9MDH5MBPbIqV92AaeXatLxBI9gBaebbnrfifHhDYfgasaacPC6xNi=xI8qiVKYPFjYdHaVhbbf9v8qqaqFr0xc9vqFj0dXdbba91qpepeI8k8fiI+fsY=rqGqVepae9pg0db9vqaiVgFr0xfr=xfr=xc9adbaqaaeGacaGaaiaabeqaaeqabiWaaaGcbaGaemOvayLaeiikaGccbeGae8NCaiNaeiykaKIaeyypa0tcfa4aaSaaaeaacqaIYaGmiiGacqGFdpWCdaWgaaqaaiabicdaWaqabaaabaGae43Wdm3aa0baaeaacqWGYbGCaeaacqGHsislaaGaey4kaSIae43Wdm3aa0baaeaacqWGYbGCaeaacqGHRaWkaaaaaOGaemOvay1aaSbaaSqaaiabicdaWaqabaGccqGGOaakcqWFYbGCcqGGPaqkcqGHRaWkjuaGdaWcaaqaaiabigdaXaqaaiabikdaYiab+b8aWbaakmaaqahabaqcfa4aaSaaaeaacqGFdpWCdaqhaaqaaiabdUgaRbqaaiabgkHiTaaacqGGFbWxcqGFdpWCdaqhaaqaaiabdUgaRbqaaiabgUcaRaaaaeaacqGFdpWCdaqhaaqaaiabdkhaYbqaaiabgkHiTaaacqGHRaWkcqGFdpWCdaqhaaqaaiabdkhaYbqaaiabgUcaRaaaaaGcdaaeWbqaamaaqahabaGaemOvay1aa0baaSqaaiabdMgaPbqaaiabdUgaRbaaaeaacqWGPbqAcqGH9aqpcqaIXaqmaeaacqWGobGtdaWgaaadbaGaem4uam1aaSbaaeaacqWGRbWAaeqaaaqabaaaniabggHiLdGcdaWdraqaaiabdIgaOnaaBaaaleaacqWGPbqAaeqaaOGaeiikaGIae8NCaiNaeiykaKscfa4aaSaaaeaacuWFYbGCgaqbaiabgkHiTiab=jhaYbqaaiabcYha8jabcYha8jqb=jhaYzaafaGaeyOeI0Iae8NCaiNaeiiFaWNaeiiFaW3aaWbaaeqabaGaeG4mamdaaaaakiabdsgaKjab=nfatnaaBaaaleaacqWFRbWAaeqaaaqaaiabfs5aenaaBaaameaacqWGtbWudaWgaaqaaiabdUgaRbqabaGaeiilaWIaemOAaOgabeaaaSqab0Gaey4kIipaaSqaaiabdQgaQjabg2da9iabigdaXaqaaiabd6eaonaaBaaameaacqWGtbWudaWgaaqaaiabdUgaRbqabaaabeaaa0GaeyyeIuoaaSqaaiabdUgaRjabg2da9iabigdaXaqaaiabdkfasbqdcqGHris5aOGaeiOla4caaa@9E31@

This equation can be transformed into a set of linear equations:

**V **= **BV **+ **V**_0_,

where **V **and **V**_0 _are column vectors denoting at every node the wanted potential value and the potential value in an infinite homogeneous medium due to a source, respectively. **B **is a matrix generated from the integrals, which depends on the geometry of the surfaces and the conductivities of each region.

Determination of the elements of the matrix **B **is computationally intensive and there exist different approaches for their computation. The integral in equation (23) is also often called the solid angle [62,64,65]. The basis functions *h*_*i*_(**r**) can be defined in several ways. The "constant-potential" approach for triangular elements uses basis functions defined by

hi(r)={1r∈Δi0r∉Δi
 MathType@MTEF@5@5@+=feaafiart1ev1aaatCvAUfKttLearuWrP9MDH5MBPbIqV92AaeXatLxBI9gBaebbnrfifHhDYfgasaacPC6xNi=xI8qiVKYPFjYdHaVhbbf9v8qqaqFr0xc9vqFj0dXdbba91qpepeI8k8fiI+fsY=rqGqVepae9pg0db9vqaiVgFr0xfr=xfr=xc9adbaqaaeGacaGaaiaabeqaaeqabiWaaaGcbaGaemiAaG2aaSbaaSqaaiabdMgaPbqabaGccqGGOaakieqacqWFYbGCcqGGPaqkcqGH9aqpdaGabaqaauaabeqaciaaaeaacqaIXaqmaeaacqWFYbGCcqGHiiIZcqqHuoardaWgaaWcbaGaemyAaKgabeaaaOqaaiabicdaWaqaaiab=jhaYjabgMGiplabfs5aenaaBaaaleaacqWGPbqAaeqaaaaaaOGaay5Eaaaaaa@4208@

where Δ_*i *_denotes the *i*th planar triangle on the tesselated surface. The collocation points are typically the centroids of the surface elements and the unknown potentials **V **are the potentials at each triangle [66]. The "linear potential" approach uses basis functions defined by

hi(r)={[r rj rk][ri rj rk]r∈Δi(jk)0r∉Δi(jk)
 MathType@MTEF@5@5@+=feaafiart1ev1aaatCvAUfKttLearuWrP9MDH5MBPbIqV92AaeXatLxBI9gBaebbnrfifHhDYfgasaacPC6xNi=xI8qiVKYPFjYdHaVhbbf9v8qqaqFr0xc9vqFj0dXdbba91qpepeI8k8fiI+fsY=rqGqVepae9pg0db9vqaiVgFr0xfr=xfr=xc9adbaqaaeGacaGaaiaabeqaaeqabiWaaaGcbaGaemiAaG2aaSbaaSqaaiabdMgaPbqabaGccqGGOaakieqacqWFYbGCcqGGPaqkcqGH9aqpdaGabaqaauaabaqaciaaaKqbagaadaWcaaqaaiabcUfaBjab=jhaYjabbccaGiab=jhaYnaaBaaabaGaemOAaOgabeaacqqGGaaicqWFYbGCdaWgaaqaaiabdUgaRbqabaGaeiyxa0fabaGaei4waSLae8NCai3aaSbaaeaacqWGPbqAaeqaaiabbccaGiab=jhaYnaaBaaabaGaemOAaOgabeaacqqGGaaicqWFYbGCdaWgaaqaaiabdUgaRbqabaGaeiyxa0faaaGcbaGae8NCaiNaeyicI4SaeuiLdq0aaSbaaSqaaiabdMgaPjabcIcaOiabdQgaQjabdUgaRjabcMcaPaqabaaakeaacqaIWaamaeaacqWFYbGCcqGHjiYZcqqHuoardaWgaaWcbaGaemyAaKMaeiikaGIaemOAaOMaem4AaSMaeiykaKcabeaaaaaakiaawUhaaaaa@62A5@

where **r**_*i*_, **r**_*j*_, **r**_*k *_are the nodes of the triangle and the triple scalar product is defined as [**r**_*i *_**r**_*j *_**r**_*k*_] = *det*(**r**_*i*_, **r**_*j*_, **r**_*k*_). The notation Δ_*i*(*jk*) _is used to indicate any triangle for which one vertex is defined by the vector **r**_*i*_, the remaining two vertices denoted as **r**_*j *_and **r**_*k*_. The function *h*_*i*_(**r**) attains a value of unity at the *i*th vertex and drops linearly to zeros at the opposite edge of all triangles to which **r**_*i *_is a vertex. In this case, the collocation points are the vertices of the elements [66]. The approaches can be expanded into higher-order elements [67]. Gençer and Tanzer investigated quadratic and cubic element types and concluded that these gave superior results to models with linear elements [68].

Barnard et al. [64] showed that the potentials in equation (27) are only defined up to an additive constant. Hence, equation (27) has no unique solution. This ambiguity can be removed by deflation, which means that **B **must be replaced by

C=B−1NeeT,
 MathType@MTEF@5@5@+=feaafiart1ev1aaatCvAUfKttLearuWrP9MDH5MBPbIqV92AaeXatLxBI9gBaebbnrfifHhDYfgasaacPC6xNi=xI8qiVKYPFjYdHaVhbbf9v8qqaqFr0xc9vqFj0dXdbba91qpepeI8k8fiI+fsY=rqGqVepae9pg0db9vqaiVgFr0xfr=xfr=xc9adbaqaaeGacaGaaiaabeqaaeqabiWaaaGcbaacbeGae83qamKaeyypa0Jae8NqaiKaeyOeI0scfa4aaSaaaeaacqaIXaqmaeaacqWGobGtaaGccqWFLbqzcqWFLbqzdaahaaWcbeqaaiabdsfaubaakiabcYcaSaaa@37D5@

where **e **is a vector with all its *N *(the total number of unknowns) components equal to one. The deflated equation

**V **= **CV **+ **V**_0_,

possesses a unique solution which is also a solution to the orignal equation (27). If **I **denotes the *N *× *N *identity matrix and **A **represents **I **- **C **then

**V **= **A**^-1^**V**_0_.

This equation can be solved using direct or iterative solvers. Direct solvers are especially usefull when the matrix **A **is relatively small because of a coarse grid. If one wants to use a fine grid, then iterative methods should be used. The use of multiple deflations during the iterations can significantly increase the convergence time to the solution of equation (31) [69].

A typical head model for solving the forward problem involves 3 layers: the brain, the skull and the scalp. The conductivity of the skull is lower than the conductivity of brain and scalp. If *β *is defined as the ratio of the skull conductivity to the brain conductivity Meijs et al. showed that an accurate solution of equation (23) is difficult to obtain for small *β *(*β *< 0.1). The large difference between the conductivities will cause an amplification of the numerical errors in the calculation. To solve this problem, the Isolated Problem Approach (IPA) can be used (also called Isolated Skull Approach), which was introduced by Hämäläinen and Sarvas [70]. Assume the labeling of the compartments as *C*_*scalp*_, *C*_*skull *_and *C*_*brain *_and *S*_*scalp *_as the outer interface, *S*_*skull *_as the interface between *C*_*skull *_and *C*_*brain *_and *S*_*brain *_as the interface between *C*_*brain *_and *C*_*skull*_. The IPA uses the following decomposition of the potential values:

*V*(**r**) = *V'*(**r**) + *V''*(**r**)

where *V'' *is the potential on surface *S*_*brain *_when the head is a homogeneous brain region, thus omitting the skull and scalp compartments. *V' *is the correction term. When V is written like above, equation (32) can be written as

V′+V′′=A−1V0V′=A−1(V0−AV′′)V′=A−1V′0.
 MathType@MTEF@5@5@+=feaafiart1ev1aaatCvAUfKttLearuWrP9MDH5MBPbIqV92AaeXatLxBI9gBaebbnrfifHhDYfgasaacPC6xNi=xI8qiVKYPFjYdHaVhbbf9v8qqaqFr0xc9vqFj0dXdbba91qpepeI8k8fiI+fsY=rqGqVepae9pg0db9vqaiVgFr0xfr=xfr=xc9adbaqaaeGacaGaaiaabeqaaeqabiWaaaGcbaqbaeWabmqaaaqaaGqabiqb=zfawzaafaGaey4kaSIaf8NvayLbauGbauaacqGH9aqpcqWFbbqqdaahaaWcbeqaaiabgkHiTiabigdaXaaakiab=zfawnaaBaaaleaacqaIWaamaeqaaaGcbaGaf8NvayLbauaacqGH9aqpcqWFbbqqdaahaaWcbeqaaiabgkHiTiabigdaXaaakiabcIcaOiab=zfawnaaBaaaleaacqaIWaamaeqaaOGaeyOeI0Iae8xqaeKaf8NvayLbauGbauaacqGGPaqkaeaacuWFwbGvgaqbaiabg2da9iab=feabnaaCaaaleqabaGaeyOeI0IaeGymaedaaOGaf8NvayLbauaadaWgaaWcbaGaeGimaadabeaakiabc6caUaaaaaa@4B5D@

Because *V'' *is zero on the interfaces *S*_*scalp *_and *S*_*skull*_, *V' *contains the potential values on the outer surface. The IPA is based on the more accurate solution of the right-hand side term V′0
 MathType@MTEF@5@5@+=feaafiart1ev1aaatCvAUfKttLearuWrP9MDH5MBPbIqV92AaeXatLxBI9gBaebbnrfifHhDYfgasaacPC6xNi=xH8viVGI8Gi=hEeeu0xXdbba9frFj0xb9qqpG0dXdb9aspeI8k8fiI+fsY=rqGqVepae9pg0db9vqaiVgFr0xfr=xfr=xc9adbaqaaeGacaGaaiaabeqaaeqabiWaaaGcbaacbeGaf8NvayLbauaadaWgaaWcbaGaeGimaadabeaaaaa@2E34@. An accurate solution can be obtained by setting V′0
 MathType@MTEF@5@5@+=feaafiart1ev1aaatCvAUfKttLearuWrP9MDH5MBPbIqV92AaeXatLxBI9gBaebbnrfifHhDYfgasaacPC6xNi=xH8viVGI8Gi=hEeeu0xXdbba9frFj0xb9qqpG0dXdb9aspeI8k8fiI+fsY=rqGqVepae9pg0db9vqaiVgFr0xfr=xfr=xc9adbaqaaeGacaGaaiaabeqaaeqabiWaaaGcbaacbeGaf8NvayLbauaadaWgaaWcbaGaeGimaadabeaaaaa@2E34@ to the following

V′0=(V′01V′02V′03)=(βV01βV02βV03−2ββ+1V′′03),
 MathType@MTEF@5@5@+=feaafiart1ev1aaatCvAUfKttLearuWrP9MDH5MBPbIqV92AaeXatLxBI9gBaebbnrfifHhDYfgasaacPC6xNi=xI8qiVKYPFjYdHaVhbbf9v8qqaqFr0xc9vqFj0dXdbba91qpepeI8k8fiI+fsY=rqGqVepae9pg0db9vqaiVgFr0xfr=xfr=xc9adbaqaaeGacaGaaiaabeqaaeqabiWaaaGcbaacbeGaf8NvayLbauaadaWgaaWcbaGaeGimaadabeaakiabg2da9maabmaabaqbaeqabmqaaaqaaiqb=zfawzaafaWaa0baaSqaaGqaaiab+bdaWaqaaiab+fdaXaaaaOqaaiqb=zfawzaafaWaa0baaSqaaiab+bdaWaqaaiab+jdaYaaaaOqaaiqb=zfawzaafaWaa0baaSqaaiab+bdaWaqaaiab+ndaZaaaaaaakiaawIcacaGLPaaacqGH9aqpdaqadaqaauaabeqadeaaaeaaiiGacqqFYoGycqWFwbGvdaqhaaWcbaGae4hmaadabaGae4xmaedaaaGcbaGae0NSdiMae8Nvay1aa0baaSqaaiab+bdaWaqaaiab+jdaYaaaaOqaaiab9j7aIjab=zfawnaaDaaaleaacqGFWaamaeaacqGFZaWmaaGccqGHsisljuaGdaWcaaqaaiabikdaYiab9j7aIbqaaiab9j7aIjabgUcaRiabigdaXaaakiqb=zfawzaafyaafaWaa0baaSqaaiab+bdaWaqaaiab+ndaZaaaaaaakiaawIcacaGLPaaacqGGSaalaaa@57C1@

where V01
 MathType@MTEF@5@5@+=feaafiart1ev1aaatCvAUfKttLearuWrP9MDH5MBPbIqV92AaeXatLxBI9gBaebbnrfifHhDYfgasaacPC6xNi=xH8viVGI8Gi=hEeeu0xXdbba9frFj0xb9qqpG0dXdb9aspeI8k8fiI+fsY=rqGqVepae9pg0db9vqaiVgFr0xfr=xfr=xc9adbaqaaeGacaGaaiaabeqaaeqabiWaaaGcbaacbeGae8Nvay1aa0baaSqaaiabicdaWaqaaiabigdaXaaaaaa@2F19@, V02
 MathType@MTEF@5@5@+=feaafiart1ev1aaatCvAUfKttLearuWrP9MDH5MBPbIqV92AaeXatLxBI9gBaebbnrfifHhDYfgasaacPC6xNi=xH8viVGI8Gi=hEeeu0xXdbba9frFj0xb9qqpG0dXdb9aspeI8k8fiI+fsY=rqGqVepae9pg0db9vqaiVgFr0xfr=xfr=xc9adbaqaaeGacaGaaiaabeqaaeqabiWaaaGcbaacbeGae8Nvay1aa0baaSqaaiabicdaWaqaaiabikdaYaaaaaa@2F1B@ and V03
 MathType@MTEF@5@5@+=feaafiart1ev1aaatCvAUfKttLearuWrP9MDH5MBPbIqV92AaeXatLxBI9gBaebbnrfifHhDYfgasaacPC6xNi=xH8viVGI8Gi=hEeeu0xXdbba9frFj0xb9qqpG0dXdb9aspeI8k8fiI+fsY=rqGqVepae9pg0db9vqaiVgFr0xfr=xfr=xc9adbaqaaeGacaGaaiaabeqaaeqabiWaaaGcbaacbeGae8Nvay1aa0baaSqaaiabicdaWaqaaiabiodaZaaaaaa@2F1D@ are the potentials at respectively the brain-skull surface, the skull-scalp surface and the outer surface. This imposes that V′′03
 MathType@MTEF@5@5@+=feaafiart1ev1aaatCvAUfKttLearuWrP9MDH5MBPbIqV92AaeXatLxBI9gBaebbnrfifHhDYfgasaacPC6xNi=xH8viVGI8Gi=hEeeu0xXdbba9frFj0xb9qqpG0dXdb9aspeI8k8fiI+fsY=rqGqVepae9pg0db9vqaiVgFr0xfr=xfr=xc9adbaqaaeGacaGaaiaabeqaaeqabiWaaaGcbaacbeGaf8NvayLbauGbauaadaqhaaWcbaGaeGimaadabaGaeG4mamdaaaaa@2F34@ has to be calculated. This can be done by solving the potentials at *S*_*brain *_with the scalp and skull compartments omitted. The increase in accuracy comes at a small cost of computational speed. A weighted IPA approach was developed by Fuchs et al. [71]. The IPA approach was extended to multi-sphere models by Gençer and Akahn-Acar [72]. The calculation of the forward problem involves every node on the mesh, making it very computation intesive. Accelerated BEM computes the node potentials on a small subset of nodes corresponding to the electrode positions [73].

To improve the localization accuracy, one can locally refine the mesh. Yvert et al. showed that if the dipole is at 2 cm below the surface, a mesh of 0.5 triangles/cm^2 ^is needed to have acceptable results. However, for shallow dipoles (between 2 mm and 20 mm below the brain surface) a mesh density of 2–6 triangles per cm^2 ^is needed to obtain comparable results. Of course, the area in the mesh that has to be refined, has to be defined.

A main disadvantage using BEM in the EEG forward problem is that in all aforementioned implementations the precision drops when the distance of the source to one of the surfaces becomes comparable to the size of the triangles in the mesh. Kybic et al. presented a new framework based on a theorem that characterizes harmonic functions defined on the complement of a bounded smooth surface [74]. Using this framework, they proposed a symmetric formulation. The main benefit of this approach is that the error increases much less dramatically when the current sources approach a surface where the conductivity is discontinuous. In another paper by the same authors, a fast multipole acceleration was used to overcome the complexity of the symmetric formulation [75]. A recent article of the same authors demonstrates that the framework allows the use of more realistical head models, which don't have to be nested. In nested head models, an inner interface is completely enveloped by an outer interface. Non-nested compartments are compartments that are not part of the brain, but part of the head (such as eyes, sinuses,...) [76].

### The finite element method

Another method to solve Poisson's equation in a realistic head model is the finite element method (FEM). The Galerkin approach [77] is used to equation (7) with boundary conditions (11), (12), (13). First, equation (7) is multiplied with a test function *φ *and then integrated over the volume *G *representing the entire head. Using Green's first identity for integration:

∫G∇φ⋅(σ∇V)dG=∫∂Gφσ∇V⋅dS−∫Gφ(∇σ∇V)dG,
 MathType@MTEF@5@5@+=feaafiart1ev1aaatCvAUfKttLearuWrP9MDH5MBPbIqV92AaeXatLxBI9gBaebbnrfifHhDYfgasaacPC6xNi=xI8qiVKYPFjYdHaVhbbf9v8qqaqFr0xc9vqFj0dXdbba91qpepeI8k8fiI+fsY=rqGqVepae9pg0db9vqaiVgFr0xfr=xfr=xc9adbaqaaeGacaGaaiaabeqaaeqabiWaaaGcbaWaa8qeaeaacqGHhis0iiGacqWFgpGzcqGHflY1cqGGOaakcqWFdpWCcqGHhis0cqWGwbGvcqGGPaqkcqWGKbazcqWGhbWrcqGH9aqpaSqaaiabdEeahbqab0Gaey4kIipakmaapebabaGae8NXdyMae83WdmNaey4bIeTaemOvayLaeyyXICncbeGae4hzaqMae43uamfaleaacqGHciITcqWGhbWraeqaniabgUIiYdGccqGHsisldaWdraqaaiab=z8aMjabcIcaOiabgEGirlab=n8aZjabgEGirlabdAfawjabcMcaPiabdsgaKjabdEeahbWcbaGaem4raCeabeqdcqGHRiI8aOGaeiilaWcaaa@5EC2@

in combination with the boundary conditions (12), yields the 'weak formulation' of the forward problem:

−∫G∇φ⋅(σ∇V)dG=∫GφImdG.
 MathType@MTEF@5@5@+=feaafiart1ev1aaatCvAUfKttLearuWrP9MDH5MBPbIqV92AaeXatLxBI9gBaebbnrfifHhDYfgasaacPC6xNi=xI8qiVKYPFjYdHaVhbbf9v8qqaqFr0xc9vqFj0dXdbba91qpepeI8k8fiI+fsY=rqGqVepae9pg0db9vqaiVgFr0xfr=xfr=xc9adbaqaaeGacaGaaiaabeqaaeqabiWaaaGcbaGaeyOeI0Yaa8qeaeaacqGHhis0iiGacqWFgpGzcqGHflY1cqGGOaakcqWFdpWCcqGHhis0cqWGwbGvcqGGPaqkcqWGKbazcqWGhbWraSqaaiabdEeahbqab0Gaey4kIipakiabg2da9maapebabaGae8NXdyMaemysaK0aaSbaaSqaaiabd2gaTbqabaGccqWGKbazcqWGhbWraSqaaiabdEeahbqab0Gaey4kIipakiabc6caUaaa@4A41@

If (*v*, *w*) = ∫_*G*_*v*(*x*, *y*, *z*)*w*(*x*, *y*, *z*)*dG *and *a*(*u*, *v*) = -(∇*v*, *σ*∇*u*), this can be written as:

*a*(*V*, *φ*) = (*I*_*m*_, *φ*)

**Figure 10:**
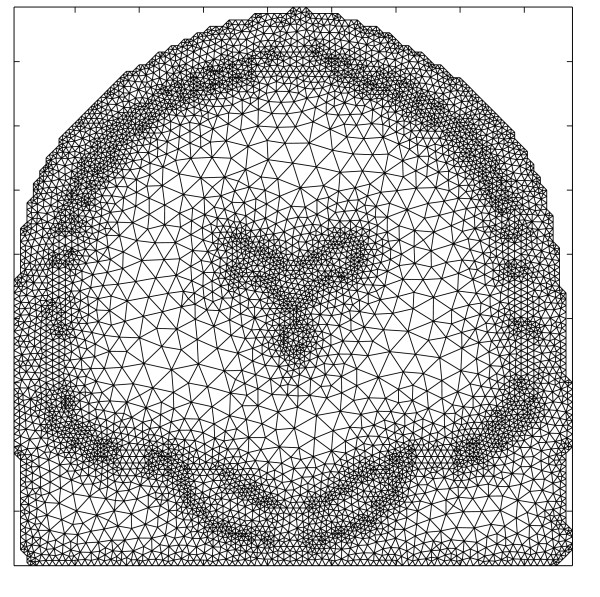
**Example mesh in 2D used in FEM**. A digitization of the 2D coronal slice of the head. The 2D elements are the triangles.

The entire 3D volume conductor is digitized in small elements. Figure 10 illustrates a 2D volume conductor digitized with triangles.

The computational points {Vi}i=1n
 MathType@MTEF@5@5@+=feaafiart1ev1aaatCvAUfKttLearuWrP9MDH5MBPbIqV92AaeXatLxBI9gBaebbnrfifHhDYfgasaacPC6xNi=xH8viVGI8Gi=hEeeu0xXdbba9frFj0xb9qqpG0dXdb9aspeI8k8fiI+fsY=rqGqVepae9pg0db9vqaiVgFr0xfr=xfr=xc9adbaqaaeGacaGaaiaabeqaaeqabiWaaaGcbaGaei4EaSNaemOvay1aaSbaaSqaaiabdMgaPbqabaGccqGG9bqFdaqhaaWcbaGaemyAaKMaeyypa0JaeGymaedabaGaemOBa4gaaaaa@367C@ can be identified with the vertices of the elements (*n *is the number of vertices). The unknown potential *V*(*x*, *y*, *z*) is given by

V(x,y,z)=∑i=1nViφi(x,y,z),
 MathType@MTEF@5@5@+=feaafiart1ev1aaatCvAUfKttLearuWrP9MDH5MBPbIqV92AaeXatLxBI9gBaebbnrfifHhDYfgasaacPC6xNi=xI8qiVKYPFjYdHaVhbbf9v8qqaqFr0xc9vqFj0dXdbba91qpepeI8k8fiI+fsY=rqGqVepae9pg0db9vqaiVgFr0xfr=xfr=xc9adbaqaaeGacaGaaiaabeqaaeqabiWaaaGcbaGaemOvayLaeiikaGIaemiEaGNaeiilaWIaemyEaKNaeiilaWIaemOEaONaeiykaKIaeyypa0ZaaabCaeaacqWGwbGvdaWgaaWcbaGaemyAaKgabeaaiiGakiab=z8aMnaaBaaaleaacqWGPbqAaeqaaOGaeiikaGIaemiEaGNaeiilaWIaemyEaKNaeiilaWIaemOEaONaeiykaKcaleaacqWGPbqAcqGH9aqpcqaIXaqmaeaacqWGUbGBa0GaeyyeIuoakiabcYcaSaaa@4C1B@

where {φi}i=1n
 MathType@MTEF@5@5@+=feaafiart1ev1aaatCvAUfKttLearuWrP9MDH5MBPbIqV92AaeXatLxBI9gBaebbnrfifHhDYfgasaacPC6xNi=xH8viVGI8Gi=hEeeu0xXdbba9frFj0xb9qqpG0dXdb9aspeI8k8fiI+fsY=rqGqVepae9pg0db9vqaiVgFr0xfr=xfr=xc9adbaqaaeGacaGaaiaabeqaaeqabiWaaaGcbaGaei4EaShcciGae8NXdy2aaSbaaSqaaiabdMgaPbqabaGccqGG9bqFdaqhaaWcbaGaemyAaKMaeyypa0JaeGymaedabaGaemOBa4gaaaaa@3707@ denotes a set of test functions also called basis functions. They have a local support, i.e. the area in which they are non-zero is limited to adjacent elements. Moreover, the basis functions span a space of piecewise polynomial functions.

Furthermore, they have the property that they are each equal to unity at the corresponding computational point and equal to zero at all other computational points. Substituting (39) in equation (38) produces *n *equations in *n *unknowns **V **= [*V*_1_...*V*_*n*_]^*T *^∈ ℝ^*n*×1^:

a(∑i=1nViφi,φj)=(Im,φj),
 MathType@MTEF@5@5@+=feaafiart1ev1aaatCvAUfKttLearuWrP9MDH5MBPbIqV92AaeXatLxBI9gBaebbnrfifHhDYfgasaacPC6xNi=xI8qiVKYPFjYdHaVhbbf9v8qqaqFr0xc9vqFj0dXdbba91qpepeI8k8fiI+fsY=rqGqVepae9pg0db9vqaiVgFr0xfr=xfr=xc9adbaqaaeGacaGaaiaabeqaaeqabiWaaaGcbaGaemyyaeMaeiikaGYaaabCaeaacqWGwbGvdaWgaaWcbaGaemyAaKgabeaaiiGakiab=z8aMnaaBaaaleaacqWGPbqAaeqaaOGaeiilaWIae8NXdy2aaSbaaSqaaiabdQgaQbqabaaabaGaemyAaKMaeyypa0JaeGymaedabaGaemOBa4ganiabggHiLdGccqGGPaqkcqGH9aqpcqGGOaakcqWGjbqsdaWgaaWcbaGaemyBa0gabeaakiabcYcaSiab=z8aMnaaBaaaleaacqWGQbGAaeqaaOGaeiykaKIaeiilaWcaaa@4ABC@

∑i=1na(φi,φj)Vi=(Im,φj)
 MathType@MTEF@5@5@+=feaafiart1ev1aaatCvAUfKttLearuWrP9MDH5MBPbIqV92AaeXatLxBI9gBaebbnrfifHhDYfgasaacPC6xNi=xI8qiVKYPFjYdHaVhbbf9v8qqaqFr0xc9vqFj0dXdbba91qpepeI8k8fiI+fsY=rqGqVepae9pg0db9vqaiVgFr0xfr=xfr=xc9adbaqaaeGacaGaaiaabeqaaeqabiWaaaGcbaWaaabCaeaacqWGHbqycqGGOaakiiGacqWFgpGzdaWgaaWcbaGaemyAaKgabeaakiabcYcaSiab=z8aMnaaBaaaleaacqWGQbGAaeqaaaqaaiabdMgaPjabg2da9iabigdaXaqaaiabd6gaUbqdcqGHris5aOGaeiykaKIaemOvay1aaSbaaSqaaiabdMgaPbqabaGccqGH9aqpcqGGOaakcqWGjbqsdaWgaaWcbaGaemyBa0gabeaakiabcYcaSiab=z8aMnaaBaaaleaacqWGQbGAaeqaaOGaeiykaKcaaa@49DC@

Due to the local support of the basis function, each equation consists only of a linear combination of *V*_*i*_'s and its adjacent computational points. Hence the system *A *∈ ℝ^*n*×*n*^, *A*_*ij *_= *a*(*φ*_*i*_, *φ*_*j*_) is sparse. In matrix notation one can obtain:

**A**·**V **= **I**,

with **I **∈ ℝ^*n*×1 ^being the column vector of the source terms obtained by the right hand side of equation (41).

An important consideration in finite-element methods is how to represent a dipole source in the model.

• The obvious direct method is to represent a dipole using a pair of fixed voltage conditions of opposite polarity applied to two adjacent nodes [78].

• Another method is to embed a dipole source in the element basis functions. When the dipole lies along the edge of an element, this approach reduces to the simple idea of using two concentrated sources at either end of that edge [78].

• A third formulation is to separate the field in two parts – one part is a standard field produced by an ideal dipole in an infinite homogeneous domain and the other part is a solution in the closed sourceless domain under boundary conditions that correct the current movement across boundaries between regions of different conductivity [78].

• In the Laplace formulation, a small volume containing the dipole is removed and fixed boundary conditions are applied at all nodes on the surface of the removed volume. This can be interpreted as replacing current sources by an estimate of the equivalent voltage sources [78].

• A fifth formulation is the blurred dipole model, where source and sink monopoles are divided over the neighbouring nodes. In most cases the source and sink monopoles do not coincide with nodes of the FEM-mesh. Therefore a way to represent the dipole is by a summation of monopoles placed at neighbouring nodes [79].

A comparison of the resulting surface potentials using the first four methods with the exact analytical solution using ideal dipoles (with an infinitesimal separation between the two poles, an infinite total current exiting one pole and entering the other, and a finite dipole moment, which is the product of the current and separation) in a 4-layer concentric sphere was made in [78]. It was found that the third formulation gives the best performance for both transverse and radial dipoles (followed by the Laplace formulation for radial dipoles).

A recent innovation [80,81] is to consider current monopoles (point sources/sinks) instead of dipoles. Using the equivalent-current inverse solution (ECS) approach for *p *grid locations, only *p *variables need to be determined in the inverse problem, whereas if a dipole is placed at each of the *p *grid locations, the solution space consists of 3*p *unknown variables because each dipole has 3 directional components. This results in an advantage of using current monopoles instead of current dipoles as demonstrated in [81] where it is shown that the time required to calculate the forward matrix in realistic finite-element head models using the conventional approach may be reduced by one third. It is further shown in [80] that ECS imaging is equivalent to the equivalent-dipole inverse solution (EDS) in that it provides source-sink distribution corresponding to the dipole sources, but additional information is needed to determine the current flows (by combining ECS and EDS estimates).

In general the stiffness matrix is very big, making the computation of the electrode potentials very computationally intensive. To solve equation (42), iterative solvers for large sparse systems are used as given in [82]. Some techniques have been proposed to reduce the computational burden and increase efficiency as will be illustrated in section 5. A freely licensed software package that implements both FEM end BEM is NEUROFEM [79,83-85].

### The finite difference method

#### Isotropic media (iFDM)

**Figure 11:**
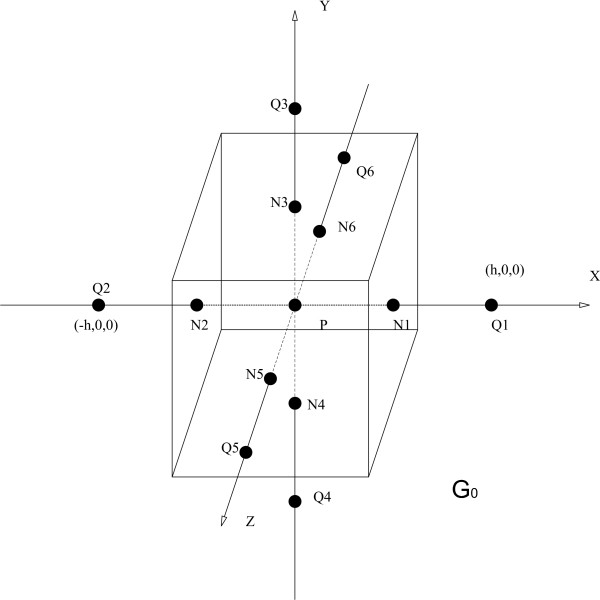
**The computation stencil used in FDM**. A typical node *P *in an FDM grid with its neighbours Q_*i *_(*i *= 1⋯6). The volume *G*_0 _is given by the box.

The differential equation (9) with boundary conditions (11), (12), (13) is transformed into a linear equation utilizing the 'box integration' scheme [86] for the cell-centered iFDM. Consider a typical node *P *in a cubic grid with internode spacing *h*. The six neighbouring nodes are *Q*_*i *_(*i *= 1,...,6) as illustrated in figure 11.

Introducing *α*_*i *_and *α*_0 _as,

αi=2hσ0σiσ0+σiα0=∑i=16αi,
 MathType@MTEF@5@5@+=feaafiart1ev1aaatCvAUfKttLearuWrP9MDH5MBPbIqV92AaeXatLxBI9gBaebbnrfifHhDYfgasaacPC6xNi=xI8qiVKYPFjYdHaVhbbf9v8qqaqFr0xc9vqFj0dXdbba91qpepeI8k8fiI+fsY=rqGqVepae9pg0db9vqaiVgFr0xfr=xfr=xc9adbaqaaeGacaGaaiaabeqaaeqabiWaaaGcbaqbaeaabiWaaaqaaGGaciab=f7aHnaaBaaaleaacqWGPbqAaeqaaaGcbaGaeyypa0dabaGaeGOmaiJaemiAaGwcfa4aaSaaaeaacqWFdpWCdaWgaaqaaiabicdaWaqabaGae83Wdm3aaSbaaeaacqWGPbqAaeqaaaqaaiab=n8aZnaaBaaabaGaeGimaadabeaacqGHRaWkcqWFdpWCdaWgaaqaaiabdMgaPbqabaaaaaGcbaGae8xSde2aaSbaaSqaaiabicdaWaqabaaakeaacqGH9aqpaeaadaaeWbqaaiab=f7aHnaaBaaaleaacqWGPbqAaeqaaaqaaiabdMgaPjabg2da9iabigdaXaqaaiabiAda2aqdcqGHris5aOGaeiilaWcaaaaa@4EA4@

a finite difference approximation of (9) is obtained:

∑i=16αiVQi−α0VP=IP,
 MathType@MTEF@5@5@+=feaafiart1ev1aaatCvAUfKttLearuWrP9MDH5MBPbIqV92AaeXatLxBI9gBaebbnrfifHhDYfgasaacPC6xNi=xI8qiVKYPFjYdHaVhbbf9v8qqaqFr0xc9vqFj0dXdbba91qpepeI8k8fiI+fsY=rqGqVepae9pg0db9vqaiVgFr0xfr=xfr=xc9adbaqaaeGacaGaaiaabeqaaeqabiWaaaGcbaWaaabCaeaaiiGacqWFXoqydaWgaaWcbaGaemyAaKgabeaakiabdAfawnaaBaaaleaacqWGrbqudaWgaaadbaGaemyAaKgabeaaaSqabaaabaGaemyAaKMaeyypa0JaeGymaedabaGaeGOnaydaniabggHiLdGccqGHsislcqWFXoqydaWgaaWcbaGaeGimaadabeaakiabdAfawnaaBaaaleaacqWGqbauaeqaaOGaeyypa0JaemysaK0aaSbaaSqaaiabdcfaqbqabaGccqGGSaalaaa@44A2@

with

IP=∫∫∫G0−Iδ(x−x2)δ(y−y2)δ(z−z2)+Iδ(x−x1)δ(y−y1)δ(z−z1)dxdydz.
 MathType@MTEF@5@5@+=feaafiart1ev1aaatCvAUfKttLearuWrP9MDH5MBPbIqV92AaeXatLxBI9gBaebbnrfifHhDYfgasaacPC6xNi=xI8qiVKYPFjYdHaVhbbf9v8qqaqFr0xc9vqFj0dXdbba91qpepeI8k8fiI+fsY=rqGqVepae9pg0db9vqaiVgFr0xfr=xfr=xc9adbaqaaeGacaGaaiaabeqaaeqabiWaaaGcbaGaemysaK0aaSbaaSqaaiabdcfaqbqabaGccqGH9aqpdaWdbaqaamaapeaabaWaa8qeaeaacqGHsislcqWGjbqsiiGacqWF0oazcqGGOaakcqWG4baEcqGHsislcqWG4baEdaWgaaWcbaGaeGOmaidabeaakiabcMcaPiab=r7aKjabcIcaOiabdMha5jabgkHiTiabdMha5naaBaaaleaacqaIYaGmaeqaaOGaeiykaKIae8hTdqMaeiikaGIaemOEaONaeyOeI0IaemOEaO3aaSbaaSqaaiabikdaYaqabaGccqGGPaqkcqGHRaWkcqWGjbqscqWF0oazcqGGOaakcqWG4baEcqGHsislcqWG4baEdaWgaaWcbaGaeGymaedabeaakiabcMcaPiab=r7aKjabcIcaOiabdMha5jabgkHiTiabdMha5naaBaaaleaacqaIXaqmaeqaaOGaeiykaKIae8hTdqMaeiikaGIaemOEaONaeyOeI0IaemOEaO3aaSbaaSqaaiabigdaXaqabaGccqGGPaqkcqWGKbazcqWG4baEcqWGKbazcqWG5bqEcqWGKbazcqWG6bGEaSqaaiabdEeahnaaBaaameaacqaIWaamaeqaaaWcbeqdcqGHRiI8aaWcbeqab0Gaey4kIipakiabc6caUaWcbeqab0Gaey4kIipaaaa@7769@

For volumes *G*, which contain a current monopole, *I*_*P *_becomes *I *or -*I*. *α*_*i *_has the dimension of Ω^-1 ^and corresponds with the conductance between *P *and *Q*_*i*_. Furthermore, for *I*_*P *_= 0 Kirchoff's law holds at the node *P*. For each node of a cubic grid we obtain a linear equation given by (44). The unknown potentials at the *n *computational points are represented by **V **∈ ℝ^*n*×1^. The source terms represented by **I **∈ ℝ^*n*×*n *^are calculated in each of the *n *cubes utilizing equation (45). Notice that in the linear equation (44) only the neighbouring computational points are included. The system matrix **A **∈ ℝ^*n*×*n *^has at most six off-diagonal elements and is a sparse matrix. In matrix notation one can write:

**A**·**V **= **I**

To solve this large sparse set of equations iterative methods are used. A discussion of the most popular solvers will be discussed in section. More extensive literature on this method can be found in [29,87-92].

#### The finite difference method in anisotropic media (aFDM)

The differential equation (7) with Dirichlet and Neumann boundary conditions can be transformed into a set of linear equations even in the case of anisotropic media. This approach uses a cubic grid in which each cube (or element) has a conductivity tensor. In anisotropic tissues the conductivity tensor can vary between neighbouring elements. There are two ways to have anisotropy. In general, the directions of the anisotropy can be in any direction. Using tensor transformations, the matrix representation of the concuctivity tensor can be deduced. In a more specific case, the directions of the anisotropy are limited along the axes of the coordinate system of the headmodel. In this case, the anisotropy is orthotrophic [93]. In the next paragraphs, the general case as shown in [94] is depicted.

If the local coordinate system coincides with the axes representing the principal directions of the anisotropy, then the conductivity tensor at an element can be written as a diagonal matrix. The diagonal elements represent the conductivities in the orthogonal directions. The matrix representation has to be transformed to the global Cartesian system of the head, the same for all elements. A rotation matrix is then required to transform the principal directions to a conductivity tensor in the Cartesian coordinate system. In the local coordinate system the conductivity tensor at an element *j *can be written as follows:

σj=(σ1i000σ2i000σ3i),
 MathType@MTEF@5@5@+=feaafiart1ev1aaatCvAUfKttLearuWrP9MDH5MBPbIqV92AaeXatLxBI9gBaebbnrfifHhDYfgasaacPC6xNi=xI8qiVKYPFjYdHaVhbbf9v8qqaqFr0xc9vqFj0dXdbba91qpepeI8k8fiI+fsY=rqGqVepae9pg0db9vqaiVgFr0xfr=xfr=xc9adbaqaaeGacaGaaiaabeqaaeqabiWaaaGcbaacciGae83Wdm3aaWbaaSqabeaacqWGQbGAaaGccqGH9aqpdaqadaqaauaabaqadmaaaeaacqWFdpWCdaqhaaWcbaGaeGymaedabaGaemyAaKgaaaGcbaGaeGimaadabaGaeGimaadabaGaeGimaadabaGae83Wdm3aa0baaSqaaiabikdaYaqaaiabdMgaPbaaaOqaaiabicdaWaqaaiabicdaWaqaaiabicdaWaqaaiab=n8aZnaaDaaaleaacqaIZaWmaeaacqWGPbqAaaaaaaGccaGLOaGaayzkaaGaeiilaWcaaa@455E@

where σij
 MathType@MTEF@5@5@+=feaafiart1ev1aaatCvAUfKttLearuWrP9MDH5MBPbIqV92AaeXatLxBI9gBaebbnrfifHhDYfgasaacPC6xNi=xH8viVGI8Gi=hEeeu0xXdbba9frFj0xb9qqpG0dXdb9aspeI8k8fiI+fsY=rqGqVepae9pg0db9vqaiVgFr0xfr=xfr=xc9adbaqaaeGacaGaaiaabeqaaeqabiWaaaGcbaacciGae83Wdm3aa0baaSqaaiabdMgaPbqaaiabdQgaQbaaaaa@3082@ are the conductivities in the principal directions at element *j*, respectively. The matrix representation has to be transformed to a global cartesian coordinate system of the head. Therefore a rotation matrix has to be applied. The matrix representation of the conductivity tensor at element *j *in the cartesian system of the head is then given by σheadj=TjTσjTj
 MathType@MTEF@5@5@+=feaafiart1ev1aaatCvAUfKttLearuWrP9MDH5MBPbIqV92AaeXatLxBI9gBaebbnrfifHhDYfgasaacPC6xNi=xH8viVGI8Gi=hEeeu0xXdbba9frFj0xb9qqpG0dXdb9aspeI8k8fiI+fsY=rqGqVepae9pg0db9vqaiVgFr0xfr=xfr=xc9adbaqaaeGacaGaaiaabeqaaeqabiWaaaGcbaacciGae83Wdm3aa0baaSqaaiabdIgaOjabdwgaLjabdggaHjabdsgaKbqaaiabdQgaQbaakiabg2da9Gqabiab+rfaunaaDaaaleaacqGFQbGAaeaacqWGubavaaGccqWFdpWCdaahaaWcbeqaaiabdQgaQbaakiab+rfaunaaBaaaleaacqGFQbGAaeqaaaaa@3F77@, where **T **is a rotation transfer matrix from the local coordinate system to the global coordinate system [95] and ^*T *^denotes the transpose of a matrix.

A finite difference method which can handle anisotropic properties of tissues was presented by Saleheen et al. [96]. Here, the authors used a transition layer technique [97] to derive a finite difference formulation for the Laplace's equation that is valid everywhere in a piecewise inhomogeneous anisotropic medium, where the conductivity tensor can have a different value in each element of the grid. This formulation is extended to Poisson's equation by Hallez et al. [94].

**Figure 12:**
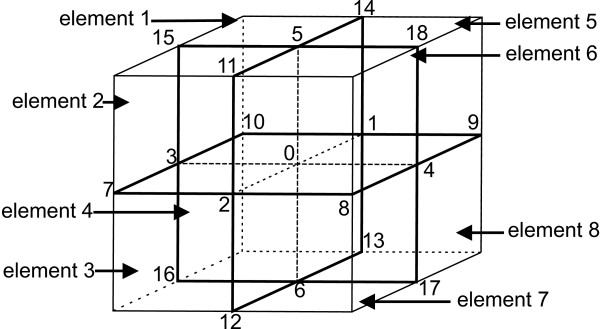
**The computation stencil used in FDM if anistropic conductivities are incorporated**. The potential at node 0 can be written as a linear combination of 18 neighbouring nodes in the FDM scheme. For each node we obtain an equation, which can be put into a linear system *Ax *= *b*.

A typical node **0 **in the grid represents the intersection of eight neighbouring cubic elements, as shown in figure 12. The finite difference formulation of equation 10 at node **0**, derived from Saleheen's method. From the 26 neigbouring nodes at node **0**, the formulation uses the 18 nearest neighbours, with rectilinear distance ≤ 2:

∑i=118aiVi−(∑i=118ai)V0=I,
 MathType@MTEF@5@5@+=feaafiart1ev1aaatCvAUfKttLearuWrP9MDH5MBPbIqV92AaeXatLxBI9gBaebbnrfifHhDYfgasaacPC6xNi=xI8qiVKYPFjYdHaVhbbf9v8qqaqFr0xc9vqFj0dXdbba91qpepeI8k8fiI+fsY=rqGqVepae9pg0db9vqaiVgFr0xfr=xfr=xc9adbaqaaeGacaGaaiaabeqaaeqabiWaaaGcbaWaaabCaeaacqWGHbqydaWgaaWcbaGaemyAaKgabeaakiabdAfawnaaBaaaleaacqWGPbqAaeqaaaqaaiabdMgaPjabg2da9iabigdaXaqaaiabigdaXiabiIda4aqdcqGHris5aOGaeyOeI0YaaeWaaeaadaaeWbqaaiabdggaHnaaBaaaleaacqWGPbqAaeqaaaqaaiabdMgaPjabg2da9iabigdaXaqaaiabigdaXiabiIda4aqdcqGHris5aaGccaGLOaGaayzkaaGaemOvay1aaSbaaSqaaiabicdaWaqabaGccqGH9aqpcqWGjbqscqGGSaalaaa@4B5B@

where *V*_*i *_is the discrete potential value at node *i*. *a*_*i *_are the coefficients depending on the conductivity tensor of the elements and the internode distance. These coefficients are given in [96].

For nodes at the corners of the compartments as illustrated in figure 12, for example node 11, the boundary normal cannot be obviously defined. Therefore, the Neumann boundary equations (12) and (11) contain singularities in spatial derivatives of the conductivities. The method presented in [94] and [96] has an advantage if one wants to enforce such a Neumann boundary condition: the formulation allows a discrete change or discontinuity in conductivity between neighbouring elements and will automatically incorporate the boundary between two different materials. In short, the boundary condition is already implicitly formulated in equation (47).

For each node a linear equation can be written as in equation (47), and for all computational points a set of linear equations is obtained: **A**·**V **= **I**. Due to the large size of the system, iterative methods have to be used.

### Comparing the various numerical methods

The three methods BEM, FEM and FDM can all be used to solve the forward problem of EEG source analysis in a realistic head model. A summary of the comparison between the BEM, FEM and FDM is given below and in table 2.

**Table 2:** A comparison of the different methods for solving Poisson's equation in a realistic head model is presented.

	BEM	FEM	iFDM	aFDM
Position of computational points	surface	volume	volume	volume
Free choice of computational points	yes	yes	no	no
System matrix	full	sparse	sparse	sparse
Solvers	direct	iterative	iterative	iterative
Number of compartments	small	large	large	large
Requires tesselation	yes	yes	no	no
Handles anisotropy	no	yes	no	yes

A first difference between BEM and FEM or FDM is the domain in which the solutions are calculated. In the BEM the solutions are calculated on the boundaries between the homogeneous isotropic compartments while in the FEM and FDM the solution of the forward problem is calculated in the entire volume. Subsequently, the FEM and FDM lead to a larger number of computational points than the BEM. On the other hand, the potential at an arbitrary point can be determined with FEM and FDM by interpolation of computational points in its vicinity, while for the BEM it is necessary to reapply the Barnard formula [62] and numerical integration.

Another important aspect is the computational efficiency. In the BEM, a full matrix (**I **- **C**), represented in equation (31), needs to be inverted. When the scalp potentials need to be known for another dipole, **V**_**0 **_in equation (31) needs to be recalculated and multiplied with the already available (**I **- **C**)^-1^. Hence once the matrix is inverted, only a matrix multiplication is needed to obtain the scalp potentials. This limited computational load is an attractive feature when solving the inverse problem, where a large number of forward evaluations need to be performed.

For the FEM and the FDM, a direct inversion of the large sparse matrices found in (42) and (46) is not possible due to the dimension of the matrices. Typically at least 500,000 computational points are considered which leads to system matrices of 500,000 equations with 500,000 unknowns which cannot be solved in a direct manner with the computers now available. However matrices found in FEM and FDM can be inverted for a given source configuration or right-hand side term, utilizing iterative solvers such as the successive over-relaxation method, the conjugate gradient method [82], or algebraic multigrid methods [98,99] (see next section). A disadvantage of the iterative solvers is that for each source configuration the solver has to be reapplied. The FEM and FDM would be computationally inefficient when for each dipole an iterative solver would need to be used. To overcome this inefficiency the reciprocity theorem is used, as will be explained in section.

When a large number of conducting compartments are introduced, a large number of boundaries need to be sampled for the BEM. This leads to a large full system matrix, thus a lower numerical efficiency. In FEM and FDM modeling, the heterogeneous nature of realistic head models will make the stiffness matrix less sparse and badly conditioned. Moreover, the incorporation of anisotropic conductivities will decrease the sparseness of the stiffness matrix. This can lead to an unstable system or very slow convergence if iterative methods are used. To obtain a fast convergence or a stable system, preconditioning should be used. Preconditioning transforms the system of equations *Ax *= *b *into a preconditioned system *M*^-1^*Ax *= *M*^-1^*b*, which has the same solution as the orignal system. *M *is a preconditioning matrix or a preconditioner and its goal is to reduce the condition number (ratio of the largest eigenvalue to the smallest eigenvalue) of the stiffness matrix towards the optimal value 1. Basic preconditioning can be used in the form of Jacobi, Gauss-Seidel, Successive Over-Relaxation (SOR) and Symmetric Successive Over-Relaxation (SSOR). These are easily implemented [100]. More advanced methods use incomplete LU factorization and polynomial preconditioning [93,100].

For the FDM in contrast with the BEM and FEM, the computational points lie fixed in the cube centers for the isotropic approach and at the cube corners for the anisotropic approach. In the FEM and BEM, the computational points, the vertices of the tetrahedrons and triangles, respectively, can be chosen more freely. Therefore, the FEM can better represent the irregular interfaces between the different compartments than the FDM, for the same amount of nodes. However, the segmented medical images used to obtain the realistic volume conductor model are constructed out of cubic voxels. It is straightforward to generate a structured grid used in FDM from these segmented images. In the FEM and the BEM, additional tessellation algorithms [101] need to be used to obtain the tetrahedron elements and the surface triangles, respectively.

Finally, it is known that the conductivities of some tissues in the human head are anisotropic such as the skull and the white matter tissue. Anisotropy can be introduced in the FEM [102] and in the FDM [96], but not in the BEM.

## Reciprocal approaches

In the literature one finds two approaches to solve the forward problem. In the conventional approach, the transfer-coefficients making up the matrix **G **in equation (17) are obtained by calculating the surface potentials from dipole sources via Poisson's equation. The calculations are made for each dipole position within the head model and the potentials at the electrode positions are recorded.

In the reciprocal approach [103], Helmholtz' principle of reciprocity is used. The electric field that results at the dipole location within the brain due to current injection and withdrawal at the surface electrode sites is first calculated. The forward transfer-coefficients are obtained from the scalar product of this electric field and the dipole moment. Calculations are thus performed for each electrode position rather than for each dipole position. This speeds up the time necessary to do the forward calculations since the number of electrodes is much smaller than the number of dipoles.

### The general idea of reciprocity

**Figure 13:**
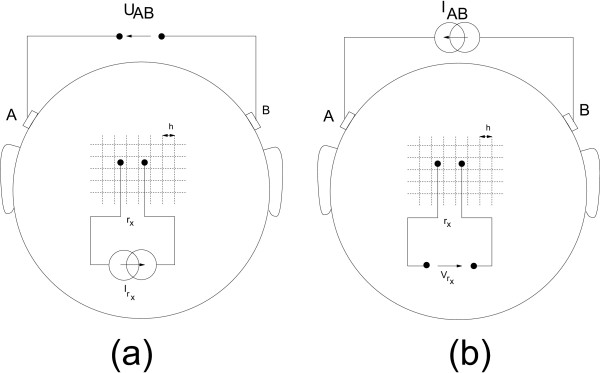
**Reciprocity**. A resistor network where a current source is introduced in the brain and the a potential difference is measured at an electrode pair, and visa versa: (a) a current source Irx
 MathType@MTEF@5@5@+=feaafiart1ev1aaatCvAUfKttLearuWrP9MDH5MBPbIqV92AaeXatLxBI9gBaebbnrfifHhDYfgasaacPC6xNi=xH8viVGI8Gi=hEeeu0xXdbba9frFj0xb9qqpG0dXdb9aspeI8k8fiI+fsY=rqGqVepae9pg0db9vqaiVgFr0xfr=xfr=xc9adbaqaaeGacaGaaiaabeqaaeqabiWaaaGcbaGaemysaK0aaSbaaSqaaiabdkhaYnaaBaaameaacqWG4baEaeqaaaWcbeaaaaa@3038@ is introduced and the potential *U*_*AB *_is measured, and (b) a current source *I*_*AB *_is introduced and a potential Vrx
 MathType@MTEF@5@5@+=feaafiart1ev1aaatCvAUfKttLearuWrP9MDH5MBPbIqV92AaeXatLxBI9gBaebbnrfifHhDYfgasaacPC6xNi=xH8viVGI8Gi=hEeeu0xXdbba9frFj0xb9qqpG0dXdb9aspeI8k8fiI+fsY=rqGqVepae9pg0db9vqaiVgFr0xfr=xfr=xc9adbaqaaeGacaGaaiaabeqaaeqabiWaaaGcbaGaemOvay1aaSbaaSqaaiabdkhaYnaaBaaameaacqWG4baEaeqaaaWcbeaaaaa@3052@ is measured.

Consider a resistor circuit, with two clamps *AB *and *r*_*x *_as illustrated in figure 13. The clamp *AB *represents a pair of scalp electrodes. The clamp *r*_*x *_is located in the brain region.

First a current Irx
 MathType@MTEF@5@5@+=feaafiart1ev1aaatCvAUfKttLearuWrP9MDH5MBPbIqV92AaeXatLxBI9gBaebbnrfifHhDYfgasaacPC6xNi=xH8viVGI8Gi=hEeeu0xXdbba9frFj0xb9qqpG0dXdb9aspeI8k8fiI+fsY=rqGqVepae9pg0db9vqaiVgFr0xfr=xfr=xc9adbaqaaeGacaGaaiaabeqaaeqabiWaaaGcbaGaemysaK0aaSbaaSqaaiabdkhaYnaaBaaameaacqWG4baEaeqaaaWcbeaaaaa@3038@ at clamp *r*_*x *_is introduced. This source will generate a potential *U*_*AB*_(Irx
 MathType@MTEF@5@5@+=feaafiart1ev1aaatCvAUfKttLearuWrP9MDH5MBPbIqV92AaeXatLxBI9gBaebbnrfifHhDYfgasaacPC6xNi=xH8viVGI8Gi=hEeeu0xXdbba9frFj0xb9qqpG0dXdb9aspeI8k8fiI+fsY=rqGqVepae9pg0db9vqaiVgFr0xfr=xfr=xc9adbaqaaeGacaGaaiaabeqaaeqabiWaaaGcbaGaemysaK0aaSbaaSqaaiabdkhaYnaaBaaameaacqWG4baEaeqaaaWcbeaaaaa@3038@) at *AB *as illustrated in figure 13(a). Next, current *I*_*AB *_at *AB *is set. This will give rise to a potential difference Vrx
 MathType@MTEF@5@5@+=feaafiart1ev1aaatCvAUfKttLearuWrP9MDH5MBPbIqV92AaeXatLxBI9gBaebbnrfifHhDYfgasaacPC6xNi=xH8viVGI8Gi=hEeeu0xXdbba9frFj0xb9qqpG0dXdb9aspeI8k8fiI+fsY=rqGqVepae9pg0db9vqaiVgFr0xfr=xfr=xc9adbaqaaeGacaGaaiaabeqaaeqabiWaaaGcbaGaemOvay1aaSbaaSqaaiabdkhaYnaaBaaameaacqWG4baEaeqaaaWcbeaaaaa@3052@(*I*_*AB*_) at *r*_*x *_illustrated in figure 13(b). The reciprocity theorem in circuit analysis states:

UABIAB=VrxIrx.
 MathType@MTEF@5@5@+=feaafiart1ev1aaatCvAUfKttLearuWrP9MDH5MBPbIqV92AaeXatLxBI9gBaebbnrfifHhDYfgasaacPC6xNi=xI8qiVKYPFjYdHaVhbbf9v8qqaqFr0xc9vqFj0dXdbba91qpepeI8k8fiI+fsY=rqGqVepae9pg0db9vqaiVgFr0xfr=xfr=xc9adbaqaaeGacaGaaiaabeqaaeqabiWaaaGcbaGaemyvau1aaSbaaSqaaiabdgeabjabdkeacbqabaGccqWGjbqsdaWgaaWcbaGaemyqaeKaemOqaieabeaakiabg2da9iabdAfawnaaBaaaleaacqWGYbGCdaWgaaadbaGaemiEaGhabeaaaSqabaGccqWGjbqsdaWgaaWcbaGaemOCai3aaSbaaWqaaiabdIha4bqabaaaleqaaOGaeiOla4caaa@3DED@

### Mathematical treatment

A mathematical treatment for a digitized volume conductor model is considered. Consider a digitized volume conductor model with *n *computational points or nodes. At each of the nodes the potential *V*_*i *_with *i *= 1...*n *is calculated for given sources which are the current monopoles *I*_*i *_with *i *= 1...*n*. Poisson's equation can then be transformed to a linear equation at each node, as illustrated for the FEM and FDM in subsections and. This set of linear equations can be written in matrix notation. The system matrix then becomes **A **∈ ℝ^*n*×*n *^and has the following properties: it is sparse, symmetric and regular. One can write:

**A**·**V **= **I**,

with **V **= [*V*_1_...*V*_*n*_]^*T *^∈ ℝ^*n*×1 ^and **I **= [*I*_1_...*I*_*n*_]^*T *^∈ ℝ^*n*×1 ^and with ^*T *^the transpose operator. The desired potential difference *V*_*k *_- *V*_*l *_between nodes *k *and *l *can be obtained for a current source *I*_*f *_at node *f *and a current sink *I*_*g *_at node *g *with *I*_*f *_= -*I*_*g*_. All other sources are zero. Cramer's solution for a linear system then becomes:

Vk=If[(−1)k+f+1Afk−(−1)k+g+1Agk]det⁡A,
 MathType@MTEF@5@5@+=feaafiart1ev1aaatCvAUfKttLearuWrP9MDH5MBPbIqV92AaeXatLxBI9gBaebbnrfifHhDYfgasaacPC6xNi=xI8qiVKYPFjYdHaVhbbf9v8qqaqFr0xc9vqFj0dXdbba91qpepeI8k8fiI+fsY=rqGqVepae9pg0db9vqaiVgFr0xfr=xfr=xc9adbaqaaeGacaGaaiaabeqaaeqabiWaaaGcbaGaemOvay1aaSbaaSqaaiabdUgaRbqabaGccqGH9aqpjuaGdaWcaaqaaiabdMeajnaaBaaabaGaemOzaygabeaacqGGBbWwcqGGOaakcqGHsislcqaIXaqmcqGGPaqkdaahaaqabeaacqWGRbWAcqGHRaWkcqWGMbGzcqGHRaWkcqaIXaqmaaGaemyqae0aaSbaaeaacqWGMbGzcqWGRbWAaeqaaiabgkHiTiabcIcaOiabgkHiTiabigdaXiabcMcaPmaaCaaabeqaaiabdUgaRjabgUcaRiabdEgaNjabgUcaRiabigdaXaaacqWGbbqqdaWgaaqaaiabdEgaNjabdUgaRbqabaGaeiyxa0fabaGagiizaqMaeiyzauMaeiiDaqhcbeGae8xqaeeaaiabcYcaSaaa@5688@

Vl=If[(−1)l+f+1Afl−(−1)l+g+1Agl]det⁡A,
 MathType@MTEF@5@5@+=feaafiart1ev1aaatCvAUfKttLearuWrP9MDH5MBPbIqV92AaeXatLxBI9gBaebbnrfifHhDYfgasaacPC6xNi=xI8qiVKYPFjYdHaVhbbf9v8qqaqFr0xc9vqFj0dXdbba91qpepeI8k8fiI+fsY=rqGqVepae9pg0db9vqaiVgFr0xfr=xfr=xc9adbaqaaeGacaGaaiaabeqaaeqabiWaaaGcbaGaemOvay1aaSbaaSqaaiabdYgaSbqabaGccqGH9aqpjuaGdaWcaaqaaiabdMeajnaaBaaabaGaemOzaygabeaacqGGBbWwcqGGOaakcqGHsislcqaIXaqmcqGGPaqkdaahaaqabeaacqWGSbaBcqGHRaWkcqWGMbGzcqGHRaWkcqaIXaqmaaGaemyqae0aaSbaaeaacqWGMbGzcqWGSbaBaeqaaiabgkHiTiabcIcaOiabgkHiTiabigdaXiabcMcaPmaaCaaabeqaaiabdYgaSjabgUcaRiabdEgaNjabgUcaRiabigdaXaaacqWGbbqqdaWgaaqaaiabdEgaNjabdYgaSbqabaGaeiyxa0fabaGagiizaqMaeiyzauMaeiiDaqhcbeGae8xqaeeaaiabcYcaSaaa@5692@

with A_*∘ _the minor for row * and column ∘.

On the other hand the potential *V*_*f *_and *V*_*g *_for a current source *I*_*k *_and current sink *I*_*l *_with *I*_*k *_= -*I*_*l*_, are:

Vf=Ik[(−1)f+k+1Akf−(−1)f+l+1Alf]det⁡A,
 MathType@MTEF@5@5@+=feaafiart1ev1aaatCvAUfKttLearuWrP9MDH5MBPbIqV92AaeXatLxBI9gBaebbnrfifHhDYfgasaacPC6xNi=xI8qiVKYPFjYdHaVhbbf9v8qqaqFr0xc9vqFj0dXdbba91qpepeI8k8fiI+fsY=rqGqVepae9pg0db9vqaiVgFr0xfr=xfr=xc9adbaqaaeGacaGaaiaabeqaaeqabiWaaaGcbaGaemOvay1aaSbaaSqaaiabdAgaMbqabaGccqGH9aqpjuaGdaWcaaqaaiabdMeajnaaBaaabaGaem4AaSgabeaacqGGBbWwcqGGOaakcqGHsislcqaIXaqmcqGGPaqkdaahaaqabeaacqWGMbGzcqGHRaWkcqWGRbWAcqGHRaWkcqaIXaqmaaGaemyqae0aaSbaaeaacqWGRbWAcqWGMbGzaeqaaiabgkHiTiabcIcaOiabgkHiTiabigdaXiabcMcaPmaaCaaabeqaaiabdAgaMjabgUcaRiabdYgaSjabgUcaRiabigdaXaaacqWGbbqqdaWgaaqaaiabdYgaSjabdAgaMbqabaGaeiyxa0fabaGagiizaqMaeiyzauMaeiiDaqhcbeGae8xqaeeaaiabcYcaSaaa@5688@

Vg=Ik[(−1)g+k+1Akg−(−1)g+l+1Alg]det⁡A.
 MathType@MTEF@5@5@+=feaafiart1ev1aaatCvAUfKttLearuWrP9MDH5MBPbIqV92AaeXatLxBI9gBaebbnrfifHhDYfgasaacPC6xNi=xI8qiVKYPFjYdHaVhbbf9v8qqaqFr0xc9vqFj0dXdbba91qpepeI8k8fiI+fsY=rqGqVepae9pg0db9vqaiVgFr0xfr=xfr=xc9adbaqaaeGacaGaaiaabeqaaeqabiWaaaGcbaGaemOvay1aaSbaaSqaaiabdEgaNbqabaGccqGH9aqpjuaGdaWcaaqaaiabdMeajnaaBaaabaGaem4AaSgabeaacqGGBbWwcqGGOaakcqGHsislcqaIXaqmcqGGPaqkdaahaaqabeaacqWGNbWzcqGHRaWkcqWGRbWAcqGHRaWkcqaIXaqmaaGaemyqae0aaSbaaeaacqWGRbWAcqWGNbWzaeqaaiabgkHiTiabcIcaOiabgkHiTiabigdaXiabcMcaPmaaCaaabeqaaiabdEgaNjabgUcaRiabdYgaSjabgUcaRiabigdaXaaacqWGbbqqdaWgaaqaaiabdYgaSjabdEgaNbqabaGaeiyxa0fabaGagiizaqMaeiyzauMaeiiDaqhcbeGae8xqaeeaaiabc6caUaaa@5696@

Furthermore, A_*∘ _is equal to *A*_∘* _due to the fact that **A **is symmetric. Hence, (eqn.(49) - eqn.(50))/*I*_*f *_equals (eqn.(51) - eqn.(52))/*I*_*k*_. Subsequently the reciprocity theorem is deduced:

*I*_*k*_(*V*_*k *_- *V*_*l*_) = *I*_*f*_(*V*_*f *_- *V*_*g*_).

### Reciprocity for a dipole source with random orientation

Considering equation (48), a dipole can be represented as two current monopoles, a current source and sink, providing Irx
 MathType@MTEF@5@5@+=feaafiart1ev1aaatCvAUfKttLearuWrP9MDH5MBPbIqV92AaeXatLxBI9gBaebbnrfifHhDYfgasaacPC6xNi=xH8viVGI8Gi=hEeeu0xXdbba9frFj0xb9qqpG0dXdb9aspeI8k8fiI+fsY=rqGqVepae9pg0db9vqaiVgFr0xfr=xfr=xc9adbaqaaeGacaGaaiaabeqaaeqabiWaaaGcbaGaemysaK0aaSbaaSqaaiabdkhaYnaaBaaameaacqWG4baEaeqaaaWcbeaaaaa@3038@ and -Irx
 MathType@MTEF@5@5@+=feaafiart1ev1aaatCvAUfKttLearuWrP9MDH5MBPbIqV92AaeXatLxBI9gBaebbnrfifHhDYfgasaacPC6xNi=xH8viVGI8Gi=hEeeu0xXdbba9frFj0xb9qqpG0dXdb9aspeI8k8fiI+fsY=rqGqVepae9pg0db9vqaiVgFr0xfr=xfr=xc9adbaqaaeGacaGaaiaabeqaaeqabiWaaaGcbaGaemysaK0aaSbaaSqaaiabdkhaYnaaBaaameaacqWG4baEaeqaaaWcbeaaaaa@3038@, separated by a distance 2*h*. The dipole is oriented from the negative to the positive current monopole and is assumed to be along the *x*-axis of the resistor network with node spacing *h*. The magnitude of the dipole moment is then 2*h*Irx
 MathType@MTEF@5@5@+=feaafiart1ev1aaatCvAUfKttLearuWrP9MDH5MBPbIqV92AaeXatLxBI9gBaebbnrfifHhDYfgasaacPC6xNi=xH8viVGI8Gi=hEeeu0xXdbba9frFj0xb9qqpG0dXdb9aspeI8k8fiI+fsY=rqGqVepae9pg0db9vqaiVgFr0xfr=xfr=xc9adbaqaaeGacaGaaiaabeqaaeqabiWaaaGcbaGaemysaK0aaSbaaSqaaiabdkhaYnaaBaaameaacqWG4baEaeqaaaWcbeaaaaa@3038@. The centre **r **of the two monopoles can then be seen as the dipole position. The scalp electrodes are located sufficiently far from the sources compared with the distance 2*h *between the sources so that we can assume a dipole field. Equation (48) can be rewritten as:

UAB=VrxIrxIAB.
 MathType@MTEF@5@5@+=feaafiart1ev1aaatCvAUfKttLearuWrP9MDH5MBPbIqV92AaeXatLxBI9gBaebbnrfifHhDYfgasaacPC6xNi=xI8qiVKYPFjYdHaVhbbf9v8qqaqFr0xc9vqFj0dXdbba91qpepeI8k8fiI+fsY=rqGqVepae9pg0db9vqaiVgFr0xfr=xfr=xc9adbaqaaeGacaGaaiaabeqaaeqabiWaaaGcbaGaemyvau1aaSbaaSqaaiabdgeabjabdkeacbqabaGccqGH9aqpjuaGdaWcaaqaaiabdAfawnaaBaaabaGaemOCai3aaSbaaeaacqWG4baEaeqaaaqabaGaemysaK0aaSbaaeaacqWGYbGCdaWgaaqaaiabdIha4bqabaaabeaaaeaacqWGjbqsdaWgaaqaaiabdgeabjabdkeacbqabaaaaOGaeiOla4caaa@3E28@

The forward problem in EEG source analysis gives the potential *U*_*AB *_for a current dipole located at **r **and oriented along the *x*-axis. Rewriting equation(53) with *d*_*x *_= 2*h*Irx
 MathType@MTEF@5@5@+=feaafiart1ev1aaatCvAUfKttLearuWrP9MDH5MBPbIqV92AaeXatLxBI9gBaebbnrfifHhDYfgasaacPC6xNi=xH8viVGI8Gi=hEeeu0xXdbba9frFj0xb9qqpG0dXdb9aspeI8k8fiI+fsY=rqGqVepae9pg0db9vqaiVgFr0xfr=xfr=xc9adbaqaaeGacaGaaiaabeqaaeqabiWaaaGcbaGaemysaK0aaSbaaSqaaiabdkhaYnaaBaaameaacqWG4baEaeqaaaWcbeaaaaa@3038@ and

∂V∂x≈[VIAB(r+hex)−VIAB(r−hex)]2h,
 MathType@MTEF@5@5@+=feaafiart1ev1aaatCvAUfKttLearuWrP9MDH5MBPbIqV92AaeXatLxBI9gBaebbnrfifHhDYfgasaacPC6xNi=xI8qiVKYPFjYdHaVhbbf9v8qqaqFr0xc9vqFj0dXdbba91qpepeI8k8fiI+fsY=rqGqVepae9pg0db9vqaiVgFr0xfr=xfr=xc9adbaqaaeGacaGaaiaabeqaaeqabiWaaaGcbaqcfa4aaSaaaeaacqGHciITcqWGwbGvaeaacqGHciITcqWG4baEaaGccqGHijYUjuaGdaWcaaqaaiabcUfaBjabdAfawnaaBaaabaGaemysaK0aaSbaaeaacqWGbbqqcqWGcbGqaeqaaaqabaGaeiikaGccbeGae8NCaiNaey4kaSIaemiAaGMae8xzau2aaSbaaeaacqWG4baEaeqaaiabcMcaPiabgkHiTiabdAfawnaaBaaabaGaemysaK0aaSbaaeaacqWGbbqqcqWGcbGqaeqaaaqabaGaeiikaGIae8NCaiNaeyOeI0IaemiAaGMae8xzau2aaSbaaeaacqWG4baEaeqaaiabcMcaPiabc2faDbqaaiabikdaYiabdIgaObaakiabcYcaSaaa@551B@

gives:

UAB=dx∂V∂xIAB.
 MathType@MTEF@5@5@+=feaafiart1ev1aaatCvAUfKttLearuWrP9MDH5MBPbIqV92AaeXatLxBI9gBaebbnrfifHhDYfgasaacPC6xNi=xI8qiVKYPFjYdHaVhbbf9v8qqaqFr0xc9vqFj0dXdbba91qpepeI8k8fiI+fsY=rqGqVepae9pg0db9vqaiVgFr0xfr=xfr=xc9adbaqaaeGacaGaaiaabeqaaeqabiWaaaGcbaGaemyvau1aaSbaaSqaaiabdgeabjabdkeacbqabaGccqGH9aqpjuaGdaWcaaqaaiabdsgaKnaaBaaabaGaemiEaGhabeaadaWcaaqaaiabgkGi2kabdAfawbqaaiabgkGi2kabdIha4baaaeaacqWGjbqsdaWgaaqaaiabdgeabjabdkeacbqabaaaaOGaeiOla4caaa@3DFD@

In a similar way, *U*_*AB *_can be calculated for a dipole located at **r **oriented along the *y*-axis and the *z*-axis.

Consider a dipole at position **r **and with dipole components **d **= (*d*_*x*_, *d*_*y*_, *d*_*z*_)^*T *^∈ ℝ^3×1^. The potential *U*_*AB *_reads:

UAB(r,d)=dT⋅∇V(r)IAB,
 MathType@MTEF@5@5@+=feaafiart1ev1aaatCvAUfKttLearuWrP9MDH5MBPbIqV92AaeXatLxBI9gBaebbnrfifHhDYfgasaacPC6xNi=xI8qiVKYPFjYdHaVhbbf9v8qqaqFr0xc9vqFj0dXdbba91qpepeI8k8fiI+fsY=rqGqVepae9pg0db9vqaiVgFr0xfr=xfr=xc9adbaqaaeGacaGaaiaabeqaaeqabiWaaaGcbaGaemyvau1aaSbaaSqaaiabdgeabjabdkeacbqabaGccqGGOaakieqacqWFYbGCcqGGSaalcqWFKbazcqGGPaqkcqGH9aqpdaWcaaqaaiab=rgaKnaaCaaaleqabaGaemivaqfaaOGaeyyXICTaey4bIeTaemOvayLaeiikaGIae8NCaiNaeiykaKcabaGaemysaK0aaSbaaSqaaiabdgeabjabdkeacbqabaaaaOGaeiilaWcaaa@4528@

with ∇*V*(**r**) = (∂*V*(**r**)/∂*x*, ∂*V*(**r**)/∂*y*, ∂*V*(**r**)/∂*z*)^*T *^∈ ℝ^3×1^.

**Figure 14:**
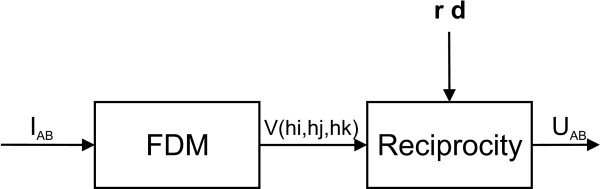
**The consecutive steps when applying reciprocity in conjunction with FDM**. A scheme showing the consecutive steps that have to be followed when applying reciprocity in conjunction with FDM. First a current dipole *I*_*AB *_is set on the electrode pair AB. Using FDM the potential field is calculated in each point *V*(*ih*, *jh*, *kh*). With the dipole parameters and the potential field, the reciprocity theorem can be applied. This results in a potential difference at the electrode pair AB.

The flowchart in figure 14 shows the consecutive steps that are necessary in the reciprocity approach in conjunction with FDM.

**Figure 15:**
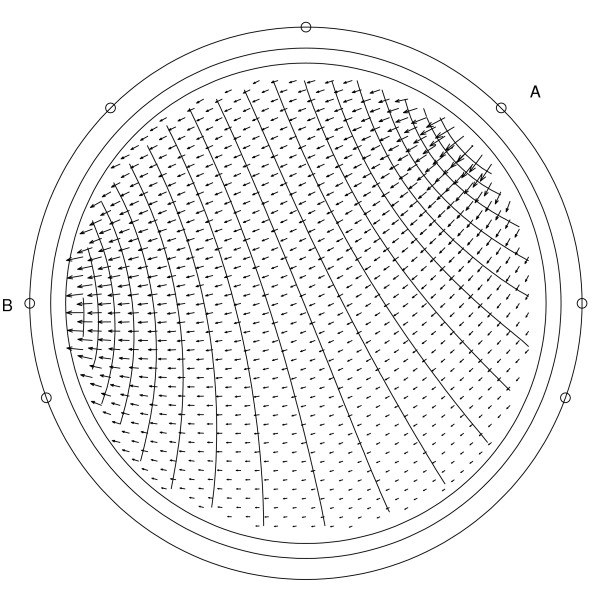
**Lead field between two electrodes**. The current density **J **= *σ*∇*V *and the equipotential lines are illustrated when introducing a current *I*_*AB *_at electrode *A *and removing the same amount at electrode *B*.

• A fictive current *I*_*AB *_of arbitrary value is introduced which enters the head at electrode *A *and leaves the head at electrode *B*.

• Utilizing the FDM the potentials *V*(*hi*, *hj*, *hk*) can be calculated with *h *the internode spacing and *i*, *j*, *k *the node numbers along the Cartesian axes. Figure 15 illustrates the equipotential lines and current density vectors **J **= -*σ*∇*V *in the brain region, with ∇*V *= (∂*V*/∂*x*, ∂*V*/∂*y*, ∂*V*/∂*z*)^*T*^. The partial derivative ∂*V*/∂*x *is approximated by [*V*(*h*(*i *+ 1), *hj*, *hk*) - *V*(*h*(*i *- 1), *hj*, *hk*)]/2*h*. The partial derivatives ∂*V*/∂*y*, ∂*V*/∂*z *are obtained in a similar way.

• *U*_*AB *_the potential difference between the scalp electrodes *A *and *B *generated by the dipole at position **r **and dipole moment **d **is obtained by applying eqn. (55). When **r **does not coincide with a node, then ∇*V*(**r**) is obtained with tri-linear interpolation [104].

By solving only one forward calculation numerically, by introducing current monopoles at electrodes *A *and *B*, and storing the obtained node potentials in a data structure, *U*_*AB *_is obtained for every dipole position and orientation.

If *N *scalp electrodes are used to measure the EEG, *N *- 1 electrode pairs can be found with linear independent potential differences. Therefore *N *- 1 numerical forward calculations are performed and stored in data structures. In addition, the *N *- 1 potential differences at the *N *- 1 electrode pairs are transformed in *N *average referenced potentials at the *N *electrodes.

Reciprocity has been applied in the literature in conjunction with BEM [105], FEM [106] and FDM [29,89,91,94,107].

## Solving large sparse linear systems applied in FEM and FDM

### Properties of the system matrix

If the linear system resulting is rewritten from equations (41), (44) and (47) in algebraic form as **Ax **= **b**, the system matrix **A **= {*a*_*ij*_} has the following properties.

From the coefficients in the linear equations one can see that the coefficient connecting a computational point *V*_*l *_to a neighbouring point *V*_*k *_is identical to the coefficient connecting *V*_*k *_to *V*_*l*_, thus **A **is symmetric. Moreover for FEM, it can be shown that the stiffness matrix *A *is a symmetric positive definite matrix [100].

For FDM in isotropic or anisotropic media, it can be shown from equation (44) that the sum of all entries in a row/column of **A **equals zero (see equations 44 and 47). Therefore, the vector **e **= [1,...,1]^*T *^is an eigenvector with associated eigenvalue 0. The matrix (**A**) of the FDM in both isotropic and anisotropic media has rank *n *- 1, with *n *the number of unknowns, and the eigenspace of the eigenvalue 0 is of dimension one. Note that for a singular problem to have a solution at all, the right-hand side **b **must be consistent, i.e. **b **∈ Range(**A**), Range(**A**) being the range of A and defined as the number of independent vectors in **A**. The kernel of A, Kernel(**A**), is the set of vectors **a **that if multiplied by **A **returns zero. In this case the problem **Ax **= **b **possesses an infinite set of solutions. An iterative method that converges from each initial guess towards an element of this solution set is said to be semi-convergent [100]. In our case, **A **is symmetric, thus Range(**A**) = Kernel(**A**)^⊥ ^where ^⊥ ^stands for the orthogonal complement. Since Kernel(**A**) is spanned by the vector **e **= (1,...,1)^*T *^containing only ones as entries, a vector **v **lies in Range(**A**) if and only if

eTv=∑k=1nvk=0.
 MathType@MTEF@5@5@+=feaafiart1ev1aaatCvAUfKttLearuWrP9MDH5MBPbIqV92AaeXatLxBI9gBaebbnrfifHhDYfgasaacPC6xNi=xI8qiVKYPFjYdHaVhbbf9v8qqaqFr0xc9vqFj0dXdbba91qpepeI8k8fiI+fsY=rqGqVepae9pg0db9vqaiVgFr0xfr=xfr=xc9adbaqaaeGacaGaaiaabeqaaeqabiWaaaGcbaacbeGae8xzau2aaWbaaSqabeaacqWGubavaaGccqWF2bGDcqGH9aqpdaaeWbqaaiabdAha2naaBaaaleaacqWGRbWAaeqaaOGaeyypa0JaeGimaadaleaacqWGRbWAcqGH9aqpcqaIXaqmaeaacqWGUbGBa0GaeyyeIuoakiabc6caUaaa@3E41@

From equations (44) and (47) it is easy to see that the right-hand side of our problem satisfies this condition. The vector, **b**, represent a dipole or a multipole, hence the sum of the elements are zero. Thus the problem **Ax **= **b **possesses infinitely many solutions differing only in an additive constant.

Instead of solving the singular linear system, another possibility is to transform it into a regular one and solve this instead. The regular problem is chosen such that its unique solution belongs to the set of solutions of the original singular system. The easiest approach is to fix the value of a computational point to 0. The special structure of the matrix then allows us to cancel the corresponding row and column in **A **and also the respective entry in the right-hand side vector **b**. This leads to a problem with a regular matrix and its solution obviously solves the initial problem with the particular computational point set zero.

Another important aspect of the matrix **A **is its sparseness. Every matrix row contains a few non-zero off-diagonal elements. This leads to a very small ratio of non-zero to overall entries resulting in a very sparse matrix.

### Iterative solvers

The following methods will be discussed:

• Successive over-relaxation (SOR)

• Conjugate gradients (CG)

• Preconditioned conjugate gradient method (PCG)

• Algebraic multigrid (AMG)

While these methods have been developed for regular linear systems, they can also be applied in our semi-definite case. In the case of a consistent right-hand side, semi-convergence can be guaranteed for SOR and (P)CG, while for AMG theoretical results are more complicated [108]. A summary of each method is given based on [104] for the first three and [109,110] for the last method.

### Successive over-relaxation

The SOR method is a representative of classical stationary methods. It is known to be a non-optimal choice as far as convergence is concerned, but has a very simple structure. Thus it is a good candidate for an optimised implementation.

A linear system of equations **Ax **= **b**,

*a*_*i*1_*x*_1 _+ ⋯ + *a*_*ii*_*x*_*i *_+ ⋯ + *a*_*in*_*x*_*n *_= *b*_*i*_,   *i *= 1,...,*n*,

can be rewritten as

xi=1aii(bi−∑j=1,j≠inaijxj).i=1,...,n,
 MathType@MTEF@5@5@+=feaafiart1ev1aaatCvAUfKttLearuWrP9MDH5MBPbIqV92AaeXatLxBI9gBaebbnrfifHhDYfgasaacPC6xNi=xI8qiVKYPFjYdHaVhbbf9v8qqaqFr0xc9vqFj0dXdbba91qpepeI8k8fiI+fsY=rqGqVepae9pg0db9vqaiVgFr0xfr=xfr=xc9adbaqaaeGacaGaaiaabeqaaeqabiWaaaGcbaqbaeqabeGaaaqaaiabdIha4naaBaaaleaacqWGPbqAaeqaaOGaeyypa0tcfa4aaSaaaeaacqaIXaqmaeaacqWGHbqydaWgaaqaaiabdMgaPjabdMgaPbqabaaaaOWaaeWaaeaacqWGIbGydaWgaaWcbaGaemyAaKgabeaakiabgkHiTmaaqahabaGaemyyae2aaSbaaSqaaiabdMgaPjabdQgaQbqabaGccqWG4baEdaWgaaWcbaGaemOAaOgabeaaaeaacqWGQbGAcqGH9aqpcqaIXaqmcqGGSaalcqWGQbGAcqGHGjsUcqWGPbqAaeaacqWGUbGBa0GaeyyeIuoaaOGaayjkaiaawMcaaiabc6caUaqaaiabdMgaPjabg2da9iabigdaXiabcYcaSiabc6caUiabc6caUiabc6caUiabcYcaSiabd6gaUjabcYcaSaaaaaa@59C5@

Let **x**^(*k*) ^be an approximation to the solution after *k *iterations. The SOR method updates the unknowns in the following fashion. To compute xi(k+1)
 MathType@MTEF@5@5@+=feaafiart1ev1aaatCvAUfKttLearuWrP9MDH5MBPbIqV92AaeXatLxBI9gBaebbnrfifHhDYfgasaacPC6xNi=xH8viVGI8Gi=hEeeu0xXdbba9frFj0xb9qqpG0dXdb9aspeI8k8fiI+fsY=rqGqVepae9pg0db9vqaiVgFr0xfr=xfr=xc9adbaqaaeGacaGaaiaabeqaaeqabiWaaaGcbaGaemiEaG3aa0baaSqaaiabdMgaPbqaaiabcIcaOiabdUgaRjabgUcaRiabigdaXiabcMcaPaaaaaa@33B7@ first an intermediate value

x¯i(k+1)=1aii(bi−∑j=1i−1aijxj(k+1)−∑j=i+1naijxj(k))
 MathType@MTEF@5@5@+=feaafiart1ev1aaatCvAUfKttLearuWrP9MDH5MBPbIqV92AaeXatLxBI9gBaebbnrfifHhDYfgasaacPC6xNi=xI8qiVKYPFjYdHaVhbbf9v8qqaqFr0xc9vqFj0dXdbba91qpepeI8k8fiI+fsY=rqGqVepae9pg0db9vqaiVgFr0xfr=xfr=xc9adbaqaaeGacaGaaiaabeqaaeqabiWaaaGcbaGafmiEaGNbaebadaqhaaWcbaGaemyAaKgabaGaeiikaGIaem4AaSMaey4kaSIaeGymaeJaeiykaKcaaOGaeyypa0tcfa4aaSaaaeaacqaIXaqmaeaacqWGHbqydaWgaaqaaiabdMgaPjabdMgaPbqabaaaaOWaaeWaaeaacqWGIbGydaWgaaWcbaGaemyAaKgabeaakiabgkHiTmaaqahabaGaemyyae2aaSbaaSqaaiabdMgaPjabdQgaQbqabaGccqWG4baEdaqhaaWcbaGaemOAaOgabaGaeiikaGIaem4AaSMaey4kaSIaeGymaeJaeiykaKcaaaqaaiabdQgaQjabg2da9iabigdaXaqaaiabdMgaPjabgkHiTiabigdaXaqdcqGHris5aOGaeyOeI0YaaabCaeaacqWGHbqydaWgaaWcbaGaemyAaKMaemOAaOgabeaakiabdIha4naaDaaaleaacqWGQbGAaeaacqGGOaakcqWGRbWAcqGGPaqkaaaabaGaemOAaOMaeyypa0JaemyAaKMaey4kaSIaeGymaedabaGaemOBa4ganiabggHiLdaakiaawIcacaGLPaaaaaa@6996@

is determined. Here new values of **x**^(*k*+1) ^are used as soon as they are available. The new approximation then becomes

xi(k+1)=ωx¯i(k+1)+(1−ω)xi(k)=xi(k)+ω(x¯i(k+1)−xi(k)).
 MathType@MTEF@5@5@+=feaafiart1ev1aaatCvAUfKttLearuWrP9MDH5MBPbIqV92AaeXatLxBI9gBaebbnrfifHhDYfgasaacPC6xNi=xI8qiVKYPFjYdHaVhbbf9v8qqaqFr0xc9vqFj0dXdbba91qpepeI8k8fiI+fsY=rqGqVepae9pg0db9vqaiVgFr0xfr=xfr=xc9adbaqaaeGacaGaaiaabeqaaeqabiWaaaGcbaGaemiEaG3aa0baaSqaaiabdMgaPbqaaiabcIcaOiabdUgaRjabgUcaRiabigdaXiabcMcaPaaakiabg2da9GGaciab=L8a3jqbdIha4zaaraWaa0baaSqaaiabdMgaPbqaaiabcIcaOiabdUgaRjabgUcaRiabigdaXiabcMcaPaaakiabgUcaRiabcIcaOiabigdaXiabgkHiTiab=L8a3jabcMcaPiabdIha4naaDaaaleaacqWGPbqAaeaacqGGOaakcqWGRbWAcqGGPaqkaaGccqGH9aqpcqWG4baEdaqhaaWcbaGaemyAaKgabaGaeiikaGIaem4AaSMaeiykaKcaaOGaey4kaSIae8xYdC3aaeWaaeaacuWG4baEgaqeamaaDaaaleaacqWGPbqAaeaacqGGOaakcqWGRbWAcqGHRaWkcqaIXaqmcqGGPaqkaaGccqGHsislcqWG4baEdaqhaaWcbaGaemyAaKgabaGaeiikaGIaem4AaSMaeiykaKcaaaGccaGLOaGaayzkaaGaeiOla4caaa@668C@

The over-relaxation parameter *ω *is a weighting parameter used to put more weight onto the correction in order to improve convergence. According to the Young theorem, the optimal value for *ω *can be computed and can be shown to be equal to:

ωopt=21+1−ρ(B)2,
 MathType@MTEF@5@5@+=feaafiart1ev1aaatCvAUfKttLearuWrP9MDH5MBPbIqV92AaeXatLxBI9gBaebbnrfifHhDYfgasaacPC6xNi=xI8qiVKYPFjYdHaVhbbf9v8qqaqFr0xc9vqFj0dXdbba91qpepeI8k8fiI+fsY=rqGqVepae9pg0db9vqaiVgFr0xfr=xfr=xc9adbaqaaeGacaGaaiaabeqaaeqabiWaaaGcbaacciGae8xYdC3aaSbaaSqaaiabd+gaVjabdchaWjabdsha0bqabaGccqGH9aqpjuaGdaWcaaqaaiabikdaYaqaaiabigdaXiabgUcaRmaakaaabaGaeGymaeJaeyOeI0Iae8xWdiNaeiikaGccbeGae4NqaiKaeiykaKYaaWbaaeqabaGaeGOmaidaaaqabaaaaOGaeiilaWcaaa@3F3E@

where *ρ*(**B**) is the spectral radius or the maximum of the absolute eigenvalues of the Jacobi iteration matrix. During the SOR procedure, the *ω *can be altered using this formula to obtain a faster convergence [100].

**Figure 16:**
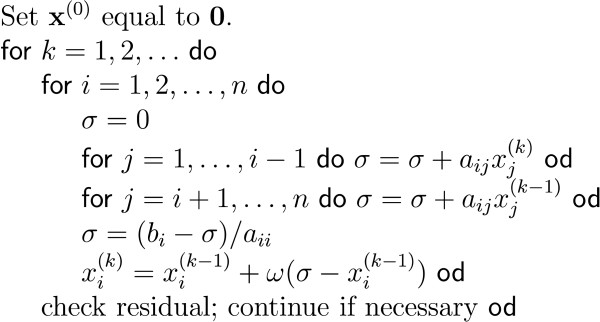
**The SOR method**. Pseudo-code for the successive over-relaxation method. The instructions to be processed in a for-loop are indicated between the do and od.

The pseudocode for the SOR algorithm is given in figure 16.

### Conjugate gradients

The CG method is the typical algorithm and is especially suited for symmetric positive definite matrices, for which it was originally devised. CG is a descendant of the method of steepest descent, that avoids repeated search in the same directions by making search directions orthogonal to each other in the energy (L2) norm associated with the matrix.

In the CG method the iterate **x**^(*k*+1) ^is computed via

**x**^(*k*+1) ^= **x**^(*k*) ^+ *α*^(*k*)^**d**^(*k*)^,

where **d**^(*k*) ^∈ ℝ^*n *^is a search direction and *α*^(*k*) ^is a scalar given by

α(k)=(r(k))Tr(k)(d(k))TAd(k).
 MathType@MTEF@5@5@+=feaafiart1ev1aaatCvAUfKttLearuWrP9MDH5MBPbIqV92AaeXatLxBI9gBaebbnrfifHhDYfgasaacPC6xNi=xI8qiVKYPFjYdHaVhbbf9v8qqaqFr0xc9vqFj0dXdbba91qpepeI8k8fiI+fsY=rqGqVepae9pg0db9vqaiVgFr0xfr=xfr=xc9adbaqaaeGacaGaaiaabeqaaeqabiWaaaGcbaacciGae8xSde2aaWbaaSqabeaacqGGOaakcqWGRbWAcqGGPaqkaaGccqGH9aqpjuaGdaWcaaqaaiabcIcaOGqabiab+jhaYnaaCaaabeqaaiabcIcaOiabdUgaRjabcMcaPaaacqGGPaqkdaahaaqabeaacqWGubavaaGae4NCai3aaWbaaeqabaGaeiikaGIaem4AaSMaeiykaKcaaaqaaiabcIcaOiab+rgaKnaaCaaabeqaaiabcIcaOiabdUgaRjabcMcaPaaacqGGPaqkdaahaaqabeaacqWGubavaaGae4xqaeKae4hzaq2aaWbaaeqabaGaeiikaGIaem4AaSMaeiykaKcaaaaakiabc6caUaaa@4CEF@

The first search direction is just the residual of the initial guess **d**^(0) ^= **r**^(0)^, where the residual is defined by **r**^(*k*) ^= **b **- **Ax**^(*k*)^. The *k*-th search direction is computed from the previous one via

**d**^(*k*) ^= **r**^(*k*) ^+ *β*^(*k*)^**d**^(*k*-1)^,

with

β(k)=(r(k))Tr(k)(r(k−1))Tr(k−1).
 MathType@MTEF@5@5@+=feaafiart1ev1aaatCvAUfKttLearuWrP9MDH5MBPbIqV92AaeXatLxBI9gBaebbnrfifHhDYfgasaacPC6xNi=xI8qiVKYPFjYdHaVhbbf9v8qqaqFr0xc9vqFj0dXdbba91qpepeI8k8fiI+fsY=rqGqVepae9pg0db9vqaiVgFr0xfr=xfr=xc9adbaqaaeGacaGaaiaabeqaaeqabiWaaaGcbaacciGae8NSdi2aaWbaaSqabeaacqGGOaakcqWGRbWAcqGGPaqkaaGccqGH9aqpjuaGdaWcaaqaaiabcIcaOGqabiab+jhaYnaaCaaabeqaaiabcIcaOiabdUgaRjabcMcaPaaacqGGPaqkdaahaaqabeaacqWGubavaaGae4NCai3aaWbaaeqabaGaeiikaGIaem4AaSMaeiykaKcaaaqaaiabcIcaOiab+jhaYnaaCaaabeqaaiabcIcaOiabdUgaRjabgkHiTiabigdaXiabcMcaPaaacqGGPaqkdaahaaqabeaacqWGubavaaGae4NCai3aaWbaaeqabaGaeiikaGIaem4AaSMaeyOeI0IaeGymaeJaeiykaKcaaaaakiabc6caUaaa@4FDD@

**Figure 17:**
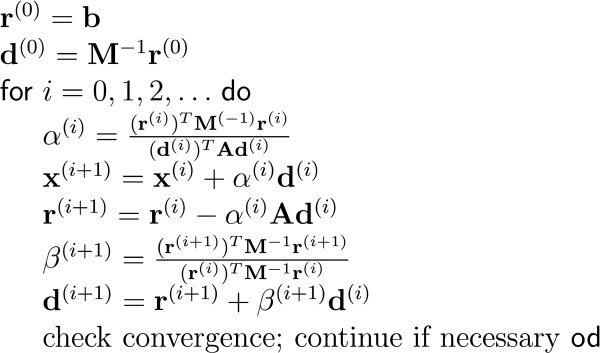
**The (P)CG method**. Pseudo-code for the preconditioned conjugate gradient method. The instructions to be processed in a for-loop are indicated between the do and od.

The pseudocode of the CG method is given in figure 17 with **M **equal to the unit matrix. Let us turn our attention now to the use of symmetric successive over-relaxation (SSOR) as a preconditioner for the CG method.

### Preconditioned conjugate gradients

The convergence of the CG method depends on the condition of the problem matrix. More precisely it is the distribution of the eigenvalues of the matrix that determines the convergence. The distribution of the eigenvalues is also known as the spectrum of a matrix. Loosely speaking, the more eigenvalues lie close together in clusters, the faster the convergence. If the eigenvalues are widely scattered, a situation one can often find for problems with a large number of unknowns, the convergence will be slow. CG is therefore seldom used without preconditioning. By preconditioning, the spectral properties of the linear system should be improved. Instead of solving **Ax **= **b**, one solves the modified system A˜x˜=b˜
 MathType@MTEF@5@5@+=feaafiart1ev1aaatCvAUfKttLearuWrP9MDH5MBPbIqV92AaeXatLxBI9gBaebbnrfifHhDYfgasaacPC6xNi=xH8viVGI8Gi=hEeeu0xXdbba9frFj0xb9qqpG0dXdb9aspeI8k8fiI+fsY=rqGqVepae9pg0db9vqaiVgFr0xfr=xfr=xc9adbaqaaeGacaGaaiaabeqaaeqabiWaaaGcbaacbeGaf8xqaeKbaGaacuWF4baEgaacaiabg2da9iqb=jgaIzaaiaaaaa@30D5@ with A˜
 MathType@MTEF@5@5@+=feaafiart1ev1aaatCvAUfKttLearuWrP9MDH5MBPbIqV92AaeXatLxBI9gBaebbnrfifHhDYfgasaacPC6xNi=xH8viVGI8Gi=hEeeu0xXdbba9frFj0xb9qqpG0dXdb9aspeI8k8fiI+fsY=rqGqVepae9pg0db9vqaiVgFr0xfr=xfr=xc9adbaqaaeGacaGaaiaabeqaaeqabiWaaaGcbaacbeGaf8xqaeKbaGaaaaa@2CF3@ = **E**^-1^**A**(**E**^-1^)^*T*^, x˜
 MathType@MTEF@5@5@+=feaafiart1ev1aaatCvAUfKttLearuWrP9MDH5MBPbIqV92AaeXatLxBI9gBaebbnrfifHhDYfgasaacPC6xNi=xH8viVGI8Gi=hEeeu0xXdbba9frFj0xb9qqpG0dXdb9aspeI8k8fiI+fsY=rqGqVepae9pg0db9vqaiVgFr0xfr=xfr=xc9adbaqaaeGacaGaaiaabeqaaeqabiWaaaGcbaacbeGaf8hEaGNbaGaaaaa@2D61@ = **E**^*T*^**x **and b˜
 MathType@MTEF@5@5@+=feaafiart1ev1aaatCvAUfKttLearuWrP9MDH5MBPbIqV92AaeXatLxBI9gBaebbnrfifHhDYfgasaacPC6xNi=xH8viVGI8Gi=hEeeu0xXdbba9frFj0xb9qqpG0dXdb9aspeI8k8fiI+fsY=rqGqVepae9pg0db9vqaiVgFr0xfr=xfr=xc9adbaqaaeGacaGaaiaabeqaaeqabiWaaaGcbaacbeGaf8NyaiMbaGaaaaa@2D35@ = **E**^-1^**b**. In practice, the matrix A˜
 MathType@MTEF@5@5@+=feaafiart1ev1aaatCvAUfKttLearuWrP9MDH5MBPbIqV92AaeXatLxBI9gBaebbnrfifHhDYfgasaacPC6xNi=xH8viVGI8Gi=hEeeu0xXdbba9frFj0xb9qqpG0dXdb9aspeI8k8fiI+fsY=rqGqVepae9pg0db9vqaiVgFr0xfr=xfr=xc9adbaqaaeGacaGaaiaabeqaaeqabiWaaaGcbaacbeGaf8xqaeKbaGaaaaa@2CF3@ is never explicitly formed. Instead in each step of the PCG algorithm a linear system of the form **Mz **= **r **must be solved with the matrix **M **= **EE**^*T *^and with **r **the residual. Compare the pseudocode of the preconditioned CG in figure 17.

### Algebraic multigrid

**Figure 18:**
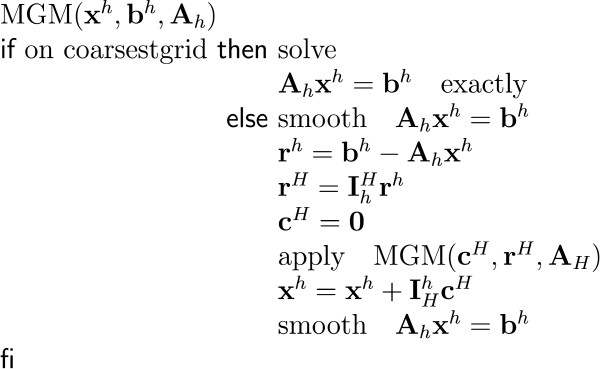
**The Multigrid V-cycle**. Pseudo-code of the Multigrid V-cylce. The instructions to be processed in a for-loop are indicated between the do and od.

The last contestant is an algebraic multigrid method. Algebraic multigrid methods are known to be, in general, very efficient solvers for elliptic boundary value problems. The basic idea is the recursive application of a two-grid method. Here one splits the error into two components. These are typically referred to as rough and smooth, because in traditional applications they represent high- and low-frequency Fourier modes. The rough components are reduced in size on the original (fine) grid by applying a limited number of steps of some iteration scheme, such as Gauß-Seidel or SOR. This process is called smoothing, because in the remaining error the smooth components are dominant. For these a correction is computed on a coarser grid with a larger mesh size. The equation for this correction is then again solved by a two-grid approach, so that a hierarchy of grid levels is obtained. Figure 18 illustrates the pseudocode of one iteration of the algebraic multigrid method. Here IhH
 MathType@MTEF@5@5@+=feaafiart1ev1aaatCvAUfKttLearuWrP9MDH5MBPbIqV92AaeXatLxBI9gBaebbnrfifHhDYfgasaacPC6xNi=xH8viVGI8Gi=hEeeu0xXdbba9frFj0xb9qqpG0dXdb9aspeI8k8fiI+fsY=rqGqVepae9pg0db9vqaiVgFr0xfr=xfr=xc9adbaqaaeGacaGaaiaabeqaaeqabiWaaaGcbaacbeGae8xsaK0aa0baaSqaaiabdIgaObqaaiabdIeaibaaaaa@2F93@ represents the transfer function from a fine grid (*h*) to the next coarser grid (*H*) and IHh
 MathType@MTEF@5@5@+=feaafiart1ev1aaatCvAUfKttLearuWrP9MDH5MBPbIqV92AaeXatLxBI9gBaebbnrfifHhDYfgasaacPC6xNi=xH8viVGI8Gi=hEeeu0xXdbba9frFj0xb9qqpG0dXdb9aspeI8k8fiI+fsY=rqGqVepae9pg0db9vqaiVgFr0xfr=xfr=xc9adbaqaaeGacaGaaiaabeqaaeqabiWaaaGcbaacbeGae8xsaK0aa0baaSqaaiabdIeaibqaaiabdIgaObaaaaa@2F93@ from a coarse grid to the next finer grid. Furthermore c* represents the correction on grid '*' applied to update the solution on the next finer grid.

**Figure 19:**
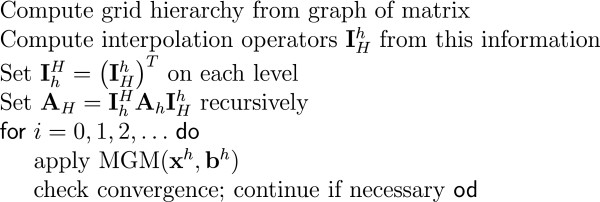
**The AMG method**. Pseudo-code of the algebraic multigrid method. The instructions to be processed in a for-loop are indicated between the do and od.

The difficulty in algebraic multigrid is finding the proper components, that is coarsening strategies to derive a suitable grid hierarchy, operators for transferring functions between different grid levels, and smoothing iterations for the rough components. In the case of complex geometries and/or jumping coefficients this can be quite tedious. Therefore the idea of algebraic multigrid methods, as illustrated in [109,110] is again attracting increased attention. Here a "grid hierarchy" and inter-grid transfer functions are derived automatically from the problem matrix. The pseudocode of the AMG method is given in figure 19.

### Comparing the iterative solvers

For the iFDM, the following conclusions by [111] were drawn from comparing the iterative solvers: (a) the algebraic multigrid-based solver performed the task 1.8–3.5 times faster, platform depending, than the second-best contender, (b) there is no need to introduce a reference potential which forces a unique solution and (c) neither the grid- nor matrix-based implementation of the solvers consistently gives a smaller run time.

Wolters et al. [79] investigated the parallel implementation of iterative solvers. If the Jacobi-CG (Jacobi Preconditioned Conjugate Gradient) solver on a single processor is taken as a reference, a speedup of 75 for a realistically shaped high resolution head model is achieved with the parallel AMG-CG (Algebraic multiGrid preconditioned congjugate gradient) solver on 12 processors. This speedup can be attributed to the algebraic multigrid preconditioning (speedup of 7.5), and to the parallelization on 12 processors (speedup of 10). The required relative solution accuracy was 10^-8^.

## Summary

The aim of this work was to give newcomers in the field of EEG source localization an overview of the state-of-the-art methods applied to solve the forward problem. Multiple references to the work of authors active in this area were provided.

The post-synaptic potentials at apical dendrites of pyramidal cells are suggested to be the generators of the EEG. The extracellular electric currents generated by these cells obey the quasi-static conditions, i.e. all currents behave as if they were stationary at each instance in time. The electrical conductivity of a tissue can be isotropic, identical in all directions (e.g. fat, cerebrospinal fluid), or, anisotropic, not identical in all directions (e.g. white matter and skull). For both cases Poisson's equation with the Neumann and Dirichlet boundary conditions was derived. The active cluster of pyramidal cells was modelled with a current dipole.

Finding the electrode potentials for a given dipole source configuration is solving the so-called forward problem. The first models used were three-shell spherical head models. Analytical solutions exist here to solve the forward problem. To have a more accurate resolution, realistically shaped head models need to be constructed. These models can be obtained by segmenting MR/CT images to extract different conducting compartments associated with certain tissues. White matter anisotropy can be obtained from MR diffusion tensor images.

Various numerical methods can be used to solve the forward problem in a realistically shaped head model. BEM, FEM, iFDM and aFDM were discussed. For BEM the computational points are located on the surfaces between isotropic conducting compartments while for the other methods the computational points are located in the entire volume. Furthermore for BEM and FEM the computational points can be chosen freely. One could, for example, place more points in areas where more irregular shapes occur. An additional tessellation algorithm to position the computational points is then required. For FDM the cubic grid is rigid. This gives the user the opportunity to import directly from 3D medical images where cubical voxels are also used. Note also that for both FEM and aFDM anisotropic compartments can be used.

The reciprocity theorem was introduced to speed up the time necessary to solve the forward problem. The electric field that results at the dipole location within the brain due to current injection and withdrawal at the surface electrode sites is first calculated. The forward transfer-coefficients are obtained from the scalar product of this electric field and the dipole moment. Calculations are thus performed for each electrode position rather than for each dipole position. This speeds up the time necessary to perform the forward calculations since the number of electrodes is much smaller than the number of dipoles.

For FEM and FDM a large linear system is generated with a sparse system matrix and a right-hand side representing the electrical sources. Solving the forward problem is solving this linear system. Direct solvers cannot be used as the number of unknowns, the potentials at the computational points, is too large. Here iterative solvers for large sparse linear systems were utilized. The unknowns are only calculated for a given right-hand side. The successive over-relaxation method, the conjugate gradient method, the preconditioned conjugate gradient method, and the multigrid method were discussed. The last method was the most promising computation-time wise.

## Discussion and new trends

In this section interesting/necessary evolutions with respect to the forward problem in EEG source localization will be discussed. The following topics are raised: (a) a promising way to obtain tissue conductivity by magnetic resonance electromagnetic impedance tomography (MREIT); (b) combined EEG/MEG source localization; (c) incorporating invasive electrodes to overcome the disadvantages of the skull; (d) dipole localization benefits of improving the SNR of the EEG by blind source separation techniques; (e) combining EEG with functional magnetic resonance imaging (fMRI) to yield more accurate localization; (f) the necessity for a grand benchmark study to compare the performance of the different numerical methods on the same dataset; (g) use of advanced numerical approaches of the FEM and/or FDM; (h) numerical approaches for dipole modelling in the forward problem.

(a) One of the main problems in EEG source localization is the uncertainty of the tissue conductivity. Although there are a lot of studies concerning this topic, the actual conductivity is not well established (and may change from person to person with age). In section 3, a distinct set of brain tissues and assigned bulk-conductivity to each of them was explained, thus obtaining a piecewise homogeneous head model. In reality the conductivity within brain tissue is place dependent and, thus, variable. The boundaries of brain tissues are in reality not discrete but a continuum. The technique to measure conductivity at a specific place in the brain is impedance tomography. A recent promising technique, MREIT, utilizes the information in both magnetic as well as electric fields (induced by injection current at the surface electrodes) to build a conductivity profile of the human head in 3D. While EIT is limited by the boundary measurements of current-voltage data, MREIT utilizes the internal magnetic flux density data obtained using a Magnetic Resonance Imaging (MRI) scanner. When a current I is injected into an electrically conducting body through a pair of surface electrodes, an electric current density **J **and a magnetic field density **B **are formed insed the conducting body. The magnetic field density **B **can be measured inside a MR scanner, **J **can be calclulated by **J **= ∇ × **B**/*μ*_0_, *μ*_0 _is the magnetic permeability in the free space. The conductivity image reconstruction problem in MREIT means to find a conductivity map *σ *from the data set [*I*, **J**_*m*_, *V*_*m*_] → *σ*, where **J**_*m *_is the measured current density inside the subject, *V*_*m *_is the measured voltage between the electrodes, and *σ *is the conductivity image to be reconstructed [112]. However, the technique is highly sensitive to noise and there is an open problem on the uniqueness of the reconstructed conductivity image. To incorporate anisotropic conductivity reconstruction one should use in addition diffusion tensor images [113]. This way the conductivity can be measured very locally [114,115]. This could not only help us establish an accurate volume conductor model but could also give us the reciprocal current sources by measurment rather than numerical calculation.

(b) Magnetoencephalography or MEG measures the induction outside the head, generated by neuronal activity. Unlike EEG, MEG is less sensitive for the conductivity of the skull. However, MEG are unable to measure radially oriented dipoles. The MEG equipment consists of superconducting quantum interference devices (SQUIDs) that can measure very low variations of magnetic field differences. A major drawback of MEG is the huge sensitivity to instrumental and environment noise. The use of superconduting elements can minimize the instrumental noise. Noise from high-frequent electromagnetic waves, like radiowaves, can be eleminated using a shielded room. Slow electromagnetic waves, like passing cars, can be minimized by the use of gradiometers. These effort to minimize the noise sources, limit the possibility to conduct a long-term monitoring. Nevertheless, the combined use of EEG and MEG has shown to be beneficial for stimulus-locked brain activations. More recently, EEG and MEG have come to be viewed as complementary rather than competing modalities. The combine EEG/MEG measurement can compensate each one's limitations and can be a very succesful modality. An accurate modelling of the human head can improve the solution of both EEG and MEG [116,117].

(c) Due to the lower conductivity of the skull compared to the surrounding tissue, the potentials generated by a source in the brain are smeared out over the scalp surface, the skull is acting as a spatially low-pass filter. In some epilepsy patients, depth- and grid electrodes are implanted in their brain. While grid electrodes are arranged in an array of 8, 16 or 32 electrodes and measure the electrical activity at the cortical surface of the brain, depth electrodes are implanted in the brain near the presumably active brain structures. These electrodes measure the electrical activity without the shielding effect of the skull. Initial studies have shown that including this information in source localization may improve the accuracy [118]. Although, the brain cannot fully be surrounded by grid electrodes, as the surgery of the patient would be to intensive. As the signal-to-noise ratio of the grid electrodes is larger than scalp electrodes, it is difficult to use both grid electrodes and scalp electrodes at the same time in the dipole estimation problem. One way to circumvent this is to create an a priori distribution of the brain activity using the grid electrodes and then use the scalp electrodes to do a more precise estimation. In the same aspect, a multipole model as a source model for intracranial EEG can become important as two dipoles with opposite orientation travel along the axon very close to each other.

(d) Noise coming from EEG background activity (i.e. other brainwaves than the ones you are interested in), artefacts from extra-cerebral sources (such as eye movements and muscle activation) and instrument quantization noise are inherent to the EEG and limit the accuracy of source localization. Removing the noise from the EEG signal is important and should be investigated. It is not necessary to incorporate anisotropic compartments when knowing that the dipole error due to noise in the EEG is of a higher magnitude. New algorithms to filter noise and artefacts from the EEG are Blind Source Separation (BSS) techniques like Independent Component Analysis (ICA) and Canonical Correlation Analysis (CCA). For more detail on these issues we refer to the literature [119-121].

(e) While the spatial resolution of the EEG is low because of the noise of the EEG signals, other modalities have a high spatial resolution. Functional magnetic resonance imaging measures locally the blood oxygen level in the brain. The fMRI scan highlights the activity present at a specific brain area. This technique has a high spatial resolution, but (unlike the EEG) has a low temporal resolution. Using both modalities, EEG and fMRI, in source localization should improve the accuracy [116,122-124].

(f) The field of forward modelling has grown extensively in the last years. Now more than ever there is need for benchmark studies to compare different numerical techniques. A conceptual benchmarking study has been done by Pruis et al. [125], but lacks a quantitative assessment of the pros and cons of the numerical methods. A benchmarking study can investigate which numerical method is preferable to others, in a specific situation. This needs an extensive and diverse database of head models accessible for researchers.

(g) Making FEM or FDM more accurate can be done in 2 ways: (i) increasing the number of nodes and (ii) increasing the order of an element. Increasing de number of nodes is equivalent to reducing element size and is know as classical (or the h-version) FEM, while increasing the order of an element is known as the p-version of FEM. The generalisation of both variants is the hp-version of FEM (hp-FEM) and is a spectral method where the convergence is achieved by simultaneously refining the mesh and increasing the approximation order. It originated in the late 1980s and is becoming very important due to its potential of unconditional exponential convergence [126,127]. The main idea is to place large high order elements in regions where the potential is smooth, and small elements with low order in regions close to singularities. One could obtain the accuracy of the system but decrease significantly the number of elements (degrees of freedom) and also the computation time [128]. However, the usefullness of hp-FEM in modeling the forward problem in EEG source localization is yet to be established.

(h) In the forward problem, the mathematical dipole is a current source and sink infinitesimally close to each other. This introduces a singularity on the right-hand side of the Poisson equation that has to be treated specifically. Several approaches exist to deal with this singularity. These approaches were summarized and benchmarked in [129]. In this article, it was shown that the potential distribution changes significantly when using different approaches for the dipole model. Further research in this field can improve our understanding in numerical methods to solve the forward problem and give insights towards correct numerical modelling of the dipole.

## Competing interests

The author(s) declare that they have no competing interests.

## Authors' contributions

BV, HH and RG participated in the literature search and were responsible for writing down the manuscript. YdA, WDC, AV, KPC, SGF, JM, SVH and IL participated in the design of the study and helped to draft the manuscript. All authors read and approved the final manuscript.
